# Supplementary material for: Electrosynthesis of buckyballs with fused-ring systems from PCBM and its analogue
Source: Nat Commun. 2023 Dec 5;14:8052. doi: 10.1038/s41467-023-43774-2 (PMC10697967; doi:10.1038/s41467-023-43774-2)
Supplement: Supplementary file 1 — Supplementary Information [file 41467_2023_43774_MOESM1_ESM.pdf]

Supplementary Information

Electrosynthesis of buckyballs with fused-ring systems from PCBM and its analogue

Wei-Feng Wang,[1] Kai-Qing Liu,[1] Chuang Niu,[1] Yun-Shu Wang,[2] Yang-Rong Yao,[3] Zheng-Chun Yin,[1] Muqing Chen,[3,4] Shi-Qi Ye,[1] Shangfeng Yang[3]* & Guan-Wu Wang[1,5]*

[1]Hefei National Research Center for Physical Sciences at the Microscale and Department of Chemistry, University of Science and Technology of China, Hefei, Anhui 230026, P. R. China.
E-mail: gwang@ustc.edu.cn

[2]Hefei No. 1 High School, Hefei, Anhui 230601, P. R. China.

[3]Hefei National Research Center for Physical Sciences at the Microscale, CAS Key Laboratory of Materials for Energy Conversion, and Department of Materials Science and Engineering, University of Science and Technology of China, Hefei, Anhui 230026, P. R. China.
E-mail: sfyang@ustc.edu.cn

[4]School of Environment and Civil Engineering, Dongguan University of Technology, Dongguan, Guangdong 523808, P. R. China.

[5]State Key Laboratory of Applied Organic Chemistry, Lanzhou University, Lanzhou, Gansu 730000, P. R. China.

Table of Contents

1. General Methods S3
2. Experimental Procedures and Spectral Data of **1**–**8** S3
3. Control Experiments S9
4. Cyclic Voltammograms of **1**–**8** and Differential Pulse Voltammograms of **3**–**8** S10
5. UV-vis Spectra of **3**–**8** S17
6. NMR Spectra of **1**–**8** S20
7. NMR Spectra of the Reaction Mixtures under Condition Screening S35
8. NMR Spectrum of the Reaction Mixture under Control Experiment S37
9. Single-Crystal X-Ray Crystallography of **3**, **4**, **7** and **8** S38
10. Calculated Partial Natural Bond Orbital Charge Distributions of **IV-1** and **IV-2** S46
11. Calculated Energies for Optimised **3**, **4**, **3''**, **6**, **6'** and **6''** S46
12. Calculated Partial Natural Bond Orbital Charge Distributions of **VII** S48
13. Calculated Energies for Optimised **VIII**−**XI** S48
14. Calculated Partial Natural Bond Orbital Charge Distributions of **VIII** S49
15. Calculated Energies for Optimised **8**, **8'** and **8''** S49
16. Characteristic Potential Values and Energy Levels of **1**–**8** S50
17. Device Fabrication and Characterization S50
18. References S52

1. General Methods

All electrochemical measurements and reactions were performed under an argon atmosphere at room temperature (~25 °C) using a Shanghai Chenhua CHI620E workstation. Tetra-*n*-butylammonium perchlorate (TBAP) was recrystallised from absolute ethanol and dried in a vacuum at 313 K prior to use. Indium tin oxide (ITO)-coated glass substrates and fluorine-doped tin oxide (FTO)-coated glass substrates were purchased from NSG Group, Japan, with a sheet resistance of 12–14 Ω/sq. PbI_2 (>98%) was obtained from TCL, and MAI (CH_3NH_3I, ≥99.5%) was purchased from Xi'an Polymer Light Technology Corp. Dimethylformamide (DMF, 99.8%), dimethylsulfoxide (DMSO, ≥99.9%) and chlorobenzene (99.9%) were obtained from Sigma-Aldrich. Isopropanol, ethanol, and acetone were all purchased from Sinopharm Chemical Reagent Co., Ltd. PCBM (99.5%) for solar cell devices was obtained from Solenne Bv, Holand. Bathocuproine (BCP) was obtained from Alfa Aesar. Other chemicals were obtained commercially and used without further purification. Controlled potential electrolysis (CPE) was carried out on a potentiostat/galvanostat using an "H"-type cell consisting of two platinum gauze electrodes (15 mm × 30 mm, serving as working and counter electrodes, respectively) separated by a sintered glass frit. A conventional three-electrode cell was used for CV measurements and consisted of a 2-mm diameter platinum disc working electrode, a platinum wire counter electrode and a saturated calomel electrode (SCE). The SCE was separated from the bulk of the solution by a fritted-glass bridge of low porosity that contained the solvent/supporting electrolyte mixture. All operations were performed under an argon atmosphere, in which the O_2 content was determined by a trace oxygen analyser (ZO-2000 Zirconia Oxygen Analyser). Compounds **1** and **2** were synthesised according to the previous literature.[1,2] NMR spectra were recorded on a 400/500/600 MHz NMR spectrometer. ^{1}H NMR chemical shifts were determined relative to TMS or residual $C_2H_2Cl_4$ (δ 5.92 ppm). ^{13}C NMR chemical shifts were determined relative to TMS or residual $C_2H_2Cl_4$ (δ 72.86 ppm). Data for ^{1}H NMR and ^{13}C NMR are reported as follows: chemical shift (δ, ppm), multiplicity (s = singlet, d = doublet, t = triplet, m = multiplet). High-resolution mass spectra (HRMS) were measured with MALDI-TOF in negative mode. Calculations were performed with the Gaussian 09 program[3] at the B3LYP/6-31G(d) level.

2. Experimental Procedures and Spectral Data of 1–8

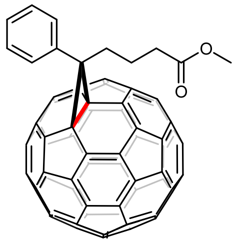

1

Synthesis of [6,6]-phenyl-C_{61}-butyric acid methyl ester (PCBM, 1).

To a solution of methyl (*E*)-5-phenyl-5-(2-tosylhydrazono)pentanoate (169 mg, 0.451 mmol) in 10 mL of anhydrous pyridine was added sodium methoxide (28 mg, 0.521 mmol), and this mixture was stirred at room temperature for 15 min under an argon atmosphere. Then, a solution of C_{60} (250 mg, 0.347 mmol) in 40 mL of 1,2-$C_6H_4Cl_2$ was added, and the mixture was heated at reflux for 48 h. The resulting mixture was purified on a silica gel column (200–300 mesh) with CS_2 to recover the unreacted C_{60}, and then CS_2/CH_2Cl_2 (3:1 v/v) to give compound **1** (98.0 mg, 31%).

^{1}H NMR (400 MHz, $CDCl_3$) δ 7.93 (d, J = 7.2 Hz, 2H), 7.55 (t, J = 7.4 Hz, 2H), 7.47 (t, J = 7.3 Hz, 1H), 3.68 (s, 3H), 2.95–2.87 (m, 2H), 2.53 (t, J = 7.5 Hz, 2H), 2.24–2.13 (m, 2H). The NMR data agreed with those in the literature.[1]

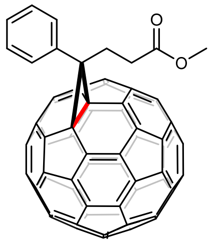

2

Synthesis of [6,6]-phenyl-C_{61}-propionic acid methyl ester (PCPM, 2).

To a solution of methyl (*E*)-4-phenyl-4-(2-tosylhydrazono)butanoate (163 mg, 0.451 mmol) in 10 mL of anhydrous pyridine was added sodium methoxide (28 mg, 0.521 mmol), and this mixture was stirred at room temperature for 15 min under an argon atmosphere. Then, a solution of C_{60} (250 mg, 0.347 mmol) in 40 mL of 1,2-$C_6H_4Cl_2$ was added, and the mixture was heated at reflux for 48 h. The resulting mixture was purified on a silica gel column (200–300 mesh) with CS_2 to recover the unreacted C_{60}, and then CS_2/CH_2Cl_2 (3:1 v/v) to give compound **2** (130.6 mg, 42%).

^{1}H NMR (400 MHz, 1:1 $CS_2/CDCl_3$) δ 7.91 (d, J = 7.2 Hz, 2H), 7.53 (t, J = 7.4 Hz, 2H), 7.46 (t, J = 7.3 Hz, 1H), 3.66 (s, 3H), 3.21 (dd, J = 7.7, 8.3 Hz, 2H), 2.85 (dd, J = 7.7, 8.3 Hz, 2H). The NMR data agreed with those in the literature.[2]

Generation of 1^{2-} and subsequent treatment with CH_2I_2.

Dianionic $\mathbf{1}^{2-}$ was electrochemically obtained from **1** (9.2 mg, 0.010 mmol) by CPE at –1.38 V vs. SCE for ca. 1 h, followed by treatment with CH_2I_2 (0.80 μL, 0.010 mmol) and stirring at 25 °C for 5 h. All operations were performed under an argon atmosphere (15.3 ppm O_2), and the gas flow rate was 2.6 mL s^{-1}. The resulting mixture was purified on a silica gel column (200–300 mesh) with CS_2/CH_2Cl_2 (1:1 v/v) to afford a mixture (3.3 mg) containing products **3** (2.27 mg, 25%), **4** (0.55 mg, 6%) and **5** (0.48 mg, 5%) based on the integrals of the methoxy group in the ^{1}H NMR spectrum (Supplementary Fig. 52). The mixture was further separated by HPLC on a Cosmosil Buckyprep column (10 mm × 250 mm) using toluene/isopropanol (7:3 v/v) as the eluent with a flow rate of 3 mL min^{-1} to obtain pure products **3**, **4** and **5**.

Generation of 1^{2-} and subsequent treatment with TFA.
Dianionic 1^{2-} was electrochemically obtained from **1** (9.2 mg, 0.010 mmol) by CPE at –1.38 V vs. SCE for ca. 1 h and then stirred at 25 °C for 5 h, followed by treatment with trifluoroacetic acid (TFA, 0.76 μL, 0.010 mmol) for 10 min. All operations were performed under an argon atmosphere, in which the O_2 content was determined to be 15.3 ppm by a trace oxygen analyser, and the gas flow rate was 2.6 mL s^{-1}. The resulting mixture was purified on a silica gel column (200–300 mesh) with CS_2/CH_2Cl_2 (1:1 v/v) to afford a mixture (5.3 mg) containing products **3** (3.63 mg, 39%), **4** (1.11 mg, 12%) and **5** (0.56 mg, 6%) based on the integrals of the methoxy group in the ^{1}H NMR spectrum (Supplementary Fig. 53). The mixture was further separated by high-performance liquid chromatography (HPLC) on a Cosmosil Buckyprep column (10 mm × 250 mm) using toluene/isopropanol (7:3 v/v) as the eluent at a flow rate of 3 mL min^{-1} to afford pure products **3**, **4** and **5**.

Dianionic 1^{2-} was obtained by electroreduction from **1** (8.9 mg, 0.010 mmol) at –1.38 V vs. SCE by CPE and the electrolysis time lasted for about 1 h. The reaction mixture was immediately treated with TFA (0.76 μL, 0.010 mmol) for 10 min. All operations were performed under an argon atmosphere (15.3 ppm O_2), and the gas flow rate was 2.6 mL s^{-1}. The resulting mixture was purified on a silica gel column (200–300 mesh) with CS_2/CH_2Cl_2 (1:1 v/v) to afford a mixture (7.0 mg) containing products **3** (2.79 mg, 31%), **4** (0.55 mg, 6%) and **5** (0.32 mg, 4%) along with recovered **1** (3.34 mg, 38%) based on the integrals in the ^{1}H NMR spectrum (Supplementary Fig. 54). The mixture was further separated by HPLC on a Cosmosil Buckyprep column (10 mm × 250 mm) using toluene/isopropanol (7:3 v/v) as the eluent with a flow rate of 3 mL min^{-1} to obtain pure products **3**, **4** and **5**.

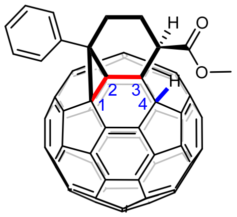

3

^{1}H NMR (400 MHz, $C_2D_2Cl_4$) δ 7.52 (d, J = 7.1 Hz, 2H), 7.35 (t, J = 7.3 Hz, 2H), 7.29 (t, J = 7.3 Hz, 1H), 6.52 (s, 1H), 4.12 (t, J = 9.7 Hz, 1H), 3.95 (s, 3H), 3.72–3.62 (m, 1H), 3.10–2.99 (m, 2H), 2.90–2.75 (m, 1H); ^{13}C NMR (101 MHz, $C_2D_2Cl_4$, all 1C unless indicated) δ 173.55 (*C*=O), 151.35, 150.67, 149.71, 149.32, 148.89, 148.14, 147.29, 147.02 (2C), 146.93, 146.03 (2C), 145.65, 145.34, 145.30, 145.05, 144.51, 144.48 (2C), 144.41, 144.35, 144.27, 143.72 (2C), 143.50, 143.36, 143.27, 143.12, 143.11, 143.03, 142.99 (2C), 142.92, 142.46, 142.31, 142.28, 142.15, 141.99, 141.91, 141.87, 141.73 (2C), 141.43, 141.34, 141.08, 140.90, 140.85, 140.74, 140.58, 139.44, 139.24, 138.67, 137.90, 137.43, 137.18 (aryl *C*), 136.83, 135.07, 129.27 (2C, aryl *C*), 127.76 (2C, aryl *C*), 126.72 (aryl *C*), 63.51 (sp^3-*C* of C_{60}), 63.41 (sp^3-*C* of C_{60}), 53.39 (sp^3-*C* of C_{60}), 51.73 (sp^3-*C* of C_{60}), 51.72, 51.12, 46.56, 27.75, 19.98; FT-IR v/cm^{-1} (KBr) 2946, 2919, 1733, 1513, 1461, 1431, 1255, 1202, 1169, 1022, 763, 697, 581, 570,

526; UV-vis ($CHCl_3$) λ_{max} nm (log ε) 258 (5.01), 325 (4.48), 436 (3.71), 639 (2.80), 704 (2.63); MALDI-TOF MS *m*/*z* calcd for $C_{72}H_{14}O_2$ $[M]^-$ 910.0999, found 910.0997.

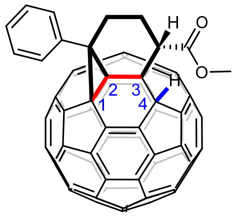

4

^{1}H NMR (500 MHz, $C_2D_2Cl_4$) δ 7.89 (d, *J* = 7.4 Hz, 2H), 7.37 (t, *J* = 7.6 Hz, 2H), 7.29 (t, *J* = 7.3 Hz, 1H), 5.95 (s, 1H), 4.44 (dd, *J* = 9.7, 6.6 Hz, 1H), 3.60 (s, 3H), 3.48 (dt, *J* = 14.5, 5.3 Hz, 1H), 3.09–3.01 (m, 1H), 2.95–2.80 (m, 2H); ^{13}C NMR (126 MHz, $C_2D_2Cl_4$, all 1C unless indicated) δ 173.89 (*C*=O), 150.24, 149.59, 149.40, 148.92, 148.91, 147.27, 147.22, 146.89, 146.80, 146.63, 146.35, 146.04, 145.95, 145.50, 145.43, 145.26, 145.01, 144.56, 144.42, 144.34, 144.27, 144.20, 143.66, 143.63, 143.45, 143.28 (2C), 143.16, 143.06, 143.02, 142.91, 142.84 (2C), 142.45, 142.42, 142.40, 142.39, 142.07, 142.00, 141.89, 141.83, 141.76, 141.36 (2C), 141.11, 140.81, 140.50, 140.47 (2C), 139.52, 139.43, 137.87, 137.67, 136.96 (aryl *C*), 136.33, 136.14, 134.85, 130.04 (2C, aryl *C*), 127.50 (2C, aryl *C*), 126.58 (aryl *C*), 64.44 (sp^3-*C* of C_{60}), 62.23 (sp^3-*C* of C_{60}), 61.46 (sp^3-*C* of C_{60}), 53.91, 51.50 (sp^3-*C* of C_{60}), 51.30, 47.67, 28.47, 20.57; FT-IR *v*/cm^{-1} (KBr) 2924, 1736, 1495, 1460, 1446, 1434, 1261, 1197, 1170, 976, 946, 810, 761, 746, 700, 579, 570, 525; UV-vis ($CHCl_3$) λ_{max} nm (log ε) 260 (5.06), 325 (4.60), 432 (3.75), 638 (2.61), 697 (2.33); MALDI-TOF MS *m*/*z* calcd for $C_{72}H_{12}O_2$ $[M]^-$ 910.0999, found 910.0991.

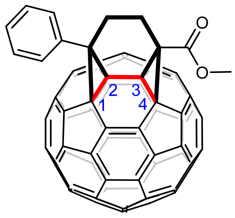

5

^{1}H NMR (400 MHz, $C_2D_2Cl_4$) δ 7.64 (d, *J* = 7.2 Hz, 2H), 7.37 (t, *J* = 7.5 Hz, 2H), 7.30 (t, *J* = 7.3 Hz, 1H), 3.89 (s, 3H), 3.57–3.45 (m, 1H), 3.41–3.26 (m, 2H), 3.00–2.87 (m, 1H); ^{13}C NMR (101 MHz, $C_2D_2Cl_4$, all 1C unless indicated) δ 167.59 (*C*=O), 150.86, 149.40, 147.81, 147.11, 146.72, 146.02, 145.98, 145.05, 144.73, 144.57 (2C), 144.51, 144.26, 144.17 (3C), 144.12, 144.00, 143.91, 143.89, 143.87, 143.80, 143.56, 143.43, 143.36, 143.25, 142.89, 142.88, 142.82, 142.69, 142.60, 142.51 (2C), 142.43 (2C), 142.28, 142.24, 142.05, 141.85, 141.67, 141.64, 141.23, 141.11 (2C), 141.06, 140.61 (3C), 140.54, 140.15 (2C), 139.33, 139.16, 138.51, 138.24 (aryl *C*), 137.22, 136.83, 129.72 (2C, aryl *C*), 127.56 (2C, aryl *C*), 126.72 (aryl *C*), 69.32 (sp^3-*C* of C_{60}), 67.77 (sp^3-*C* of C_{60}), 57.50 (sp^3-*C* of C_{60}), 56.76 (sp^3-*C* of C_{60}), 52.29, 45.40, 39.80, 29.90, 23.29; FT-IR *v*/cm^{-1} (KBr) 2923, 2852, 1729, 1511, 1460, 1432, 1262, 1096, 1022, 803, 757, 698, 570, 527; UV-vis ($CHCl_3$) λ_{max} nm (log ε) 260 (5.13), 329 (4.60), 404 (3.77), 432 (3.62), 467 (3.43), 484 (3.39), 557 (3.05), 590 (2.89), 642 (2.74), 693 (2.57);

MALDI-TOF MS *m/z* calcd for $C_{72}H_{12}O_2$ [M]$^-$ 908.0843, found 908.0847.

Generation of $2^{2–}$ and subsequent treatment without or with TFA.

Dianionic **2**$^{2–}$ was electrochemically obtained from **2** (9.2 mg, 0.010 mmol) by CPE at –1.34 V vs. SCE for ca. 1 h and then stirred at 25 °C for 3 h. All operations were performed under an argon atmosphere (15.3 ppm O_2), and the gas flow rate was 2.6 mL s$^{–1}$. The resulting mixture was filtered and then purified on a silica gel column (300–400 mesh) with CS_2 as the eluent to give products **6** (1.0 mg, 11%) and **7** (1.3 mg, 14%).

Dianionic **2**$^{2–}$ was electrochemically obtained from **2** (8.8 mg, 0.010 mmol) by CPE at –1.34 V vs. SCE for ca. 1 h, followed by immediate treatment with TFA (0.76 µL, 0.010 mmol) for 10 min. All operations were performed under an argon atmosphere (15.3 ppm O_2), and the gas flow rate was 2.6 mL s$^{–1}$. The resulting mixture was filtered and then purified on a silica gel column (300–400 mesh) with CS_2 as the eluent to give product **6** (2.8 mg, 32%).

Dianionic **2**$^{2–}$ was electrochemically obtained from **2** (8.9 mg, 0.010 mmol) by CPE at –1.34 V vs. SCE for ca. 1 h and then stirred at 25 °C for 3 h, followed by treatment with TFA (0.76 µL, 0.010 mmol) for 10 min. All operations were performed under an argon atmosphere (15.3 ppm O_2), and the gas flow rate was 2.6 mL s$^{–1}$. The resulting mixture was purified on a silica gel column (200–300 mesh) with CS_2/CH_2Cl_2 (1:1 v/v) to afford a mixture containing products **6** and **8**, which was further separated by HPLC on a Cosmosil Buckyprep column (10 mm × 250 mm) using toluene as the eluent with a flow rate of 3 mL min$^{–1}$. From the ^{1}H NMR spectrum of the mixture (3.1 mg), **6** (1.87 mg, 21%) and **8** (1.23 mg, 14%) were afforded as amorphous brown solids (Supplementary Fig. 55).

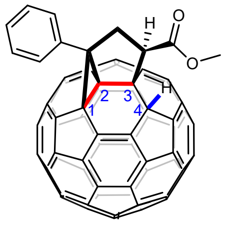

6

^{1}H NMR (500 MHz, 1:1 $CS_2/CDCl_3$) δ 7.67 (d, J = 7.4 Hz, 2H), 7.42 (t, J = 7.4 Hz, 2H), 7.36 (t, J = 7.3 Hz, 1H), 5.99 (s, 1H), 5.01 (dd, J = 11.9, 6.0 Hz, 1H), 4.37 (dd, J = 14.5, 11.9 Hz, 1H), 4.11 (s, 3H), 3.63 (dd, J = 14.5, 6.0 Hz, 1H); ^{13}C NMR (126 MHz, 1:1 $CS_2/CDCl_3$, all 1C unless indicated) δ 171.32 (*C*=O), 152.05, 151.49, 151.44, 150.89, 149.67, 148.52, 148.09, 148.00, 147.72, 147.15, 147.06, 147.02, 146.70, 146.53, 146.28, 145.84 (3C), 145.52, 145.43, 145.39, 145.10, 144.83, 144.79 (2C), 144.76, 144.51, 144.46, 144.28, 144.25, 144.15, 143.65, 143.56, 143.52, 143.51, 143.32, 143.29, 143.20, 142.95 (2C), 142.91, 142.86, 142.55, 142.39, 142.28, 142.12, 141.88, 141.81 (2C), 141.79, 141.45, 141.15, 141.07, 140.32, 137.78 (aryl *C*), 136.60, 136.17, 130.23 (2C, aryl *C*), 128.81 (2C, aryl *C*), 128.10 (aryl *C*), 71.98 (sp^3-*C* of C_{60}), 67.60 (sp^3-*C* of C_{60}), 65.96 (sp^3-*C* of C_{60}), 63.33 (sp^3-*C* of C_{60}), 56.15, 52.83, 52.49, 43.16; FT-IR ν/cm^{-1} (KBr) 2922, 2853, 1732, 1512, 1431, 1262, 1211, 1151, 761, 696,

568, 525; UV-vis ($CHCl_3$) λ_{max} nm (log ε) 259 (5.06), 326 (4.52), 437 (3.74), 631 (2.70), 695 (2.49); MALDI-TOF MS *m*/*z* calcd for $C_{71}H_{12}O_2$ $[M]^-$ 896.0843, found 896.0839.

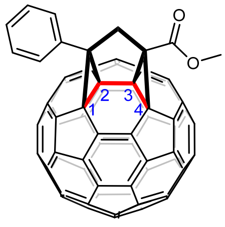

7

^{1}H NMR (500 MHz, $C_2D_2Cl_4$) δ 7.63 (d, *J* = 7.4 Hz, 2H), 7.42 (t, *J* = 7.3 Hz, 2H), 7.35 (t, *J* = 7.1 Hz, 1H), 3.82 (s, 3H), 3.67 (d, *J* = 14.8 Hz, 1H), 3.19 (d, *J* = 14.8 Hz, 1H); ^{13}C NMR (126 MHz, $C_2D_2Cl_4$, all 1C unless indicated) δ 167.46 (*C*=O), 150.53, 144.70, 144.65, 144.33, 144.31, 144.11, 144.05, 144.00, 143.92, 143.84, 143.80, 143.79, 143.68, 143.58, 143.30, 143.28, 143.18, 143.15, 143.12, 143.02, 142.93, 142.92, 142.79, 142.59, 142.53, 142.31 (4C), 142.25 (2C), 142.13, 141.88, 141.85, 141.79, 141.70 (2C), 141.68, 141.65, 141.45, 141.27, 141.22 (2C), 141.16, 141.05 (2C), 140.93, 140.61, 140.43, 140.41, 139.79, 139.42 (2C), 139.30 (2C), 138.49, 136.92 (aryl *C*), 128.16 (2C, aryl *C*), 127.55 (aryl *C*), 126.70 (2C, aryl *C*), 95.87 (sp^3-*C* of C_{60}), 91.90 (sp^3-*C* of C_{60}), 89.89 (sp^3-*C* of C_{60}), 88.95 (sp^3-*C* of C_{60}), 54.41, 52.31, 52.14, 48.64; FT-IR ν/cm^{-1} (KBr) 2946, 2850, 1732, 1512, 1431, 1262, 1212, 1151, 761, 727, 696, 568, 525; UV-vis ($CHCl_3$) λ_{max} nm (log ε) 261 (5.01), 332 (4.47), 437 (3.56), 459 (3.34), 671 (2.69); MALDI-TOF MS *m*/*z* calcd for $C_{71}H_{10}O_2$ $[M]^-$ 894.0686, found 894.0675.

Synthesis of product 8.

Dianionic $\mathbf{2}^{2.5-}$ was electrochemically obtained from **2** (8.8 mg, 0.010 mmol) by CPE at −1.34 V vs. SCE for ca. 1.5 h then stirred at 25 °C for 5 h, followed by treatment with TFA (3.8 μL, 0.050 mmol) for 10 min. All operations were performed under an argon atmosphere (15.3 ppm O_2), and the gas flow rate was 2.6 mL s^{-1}. The resulting mixture was filtered and then purified on a silica gel column (300–400 mesh) with CS_2 as the eluent to give product **8** (3.8 mg, 43%).

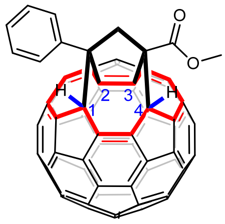

8

^{1}H NMR (500 MHz, 1:1 $CS_2/C_2D_2Cl_4$) δ 8.06 (d, *J* = 7.4 Hz, 2H), 7.54 (t, *J* = 7.8 Hz, 2H), 7.41 (t, *J* = 7.4 Hz, 1H), 6.92 (d, *J* = 2.4 Hz, 1H), 6.87 (d, *J* = 2.4 Hz, 1H), 4.37 (d, *J* = 13.4 Hz, 1H), 4.00 (s, 3H), 3.83 (d, *J* = 13.4 Hz, 1H); ^{13}C NMR (151 MHz, 1:1 $CS_2/C_2D_2Cl_4$, all 1C unless indicated) δ 171.89 (*C*=O), 151.26, 150.93, 149.50, 148.04 (2C), 147.05 (2C), 146.65 (2C), 146.37, 146.33, 146.22, 145.46, 145.43, 144.91, 144.88, 144.22 (2C), 144.12, 144.08, 144.02 (2C), 143.97 (3C), 143.14, 142.71, 142.69, 142.60,

142.24, 142.14, 142.10, 142.04, 141.82, 141.80, 141.78, 141.67 (2C), 141.32, 141.25, 141.13, 141.06, 140.81, 140.06, 138.68, 138.60, 138.48 (2C), 137.49, 137.31, 137.17, 136.08, 135.94, 135.80, 135.71, 135.69, 134.42, 134.26, 132.89, 128.29 (2C, aryl *C*), 126.83 (aryl *C*), 125.52 (2C, aryl *C*), 65.90 (sp^3-*C* of C_{60}), 59.79 (sp^3-*C* of C_{60}), 54.98, 53.44, 53.20, 52.25; FT-IR ν/cm^{-1} (KBr) 2922, 2850, 1732, 1512, 1431, 1262, 1211, 1151, 761, 696, 568, 525; UV-vis ($CHCl_3$) λ_{max} nm (log ε) 257 (5.02), 330 (4.47), 381 (4.11), 436 (3.81), 530 (3.26), 604 (2.97), 670 (2.55); MALDI-TOF MS *m*/*z* calcd for $C_{71}H_{12}O_2$ $[M]^-$ 896.0843, found 896.0852.

3. Control Experiments

Electrochemical conversion of 7 to 8:
Dianionic $\mathbf{7}^{2-}$ was electrochemically obtained from **7** (9.0 mg, 0.010 mmol) by CPE at –1.13 V vs. SCE for ca. 2 h, followed by treatment with TFA (3.8 μL, 0.050 mmol) for 10 min. All operations were performed under an argon atmosphere (15.3 ppm O_2), and the gas flow rate was 2.6 mL s^{-1}. The resulting mixture was purified on a silica gel column (200–300 mesh) with CS_2/CH_2Cl_2 (1:1 v/v) to afford a mixture of products **7** and **8**, which was further separated by HPLC on a Cosmosil Buckyprep column (10 mm × 250 mm) using toluene as the eluent with a flow rate of 3 mL min^{-1}. From the ^{1}H NMR spectrum of the mixture (7.5 mg), **8** (6.51 mg, 72%) was afforded as an amorphous brown solid (Supplementary Fig. 56).

Synthesis of Compound 8 under an argon atmosphere (3.8 ppm O_2).
Dianionic $\mathbf{2}^{2.5-}$ was electrochemically obtained from **2** (9.2 mg, 0.010 mmol) by CPE at –1.34 V vs. SCE for ca. 1 h and then stirred at 25 °C for 5 h, followed by treatment with TFA (3.8 μL, 0.050 mmol) for 10 min. All operations were performed under an argon atmosphere (3.8 ppm O_2), and the gas flow rate was 2.6 mL s^{-1}. The resulting mixture was filtered and then purified on a silica gel column (300–400 mesh) with CS_2 as the eluent to give product **8** (3.6 mg, 39%).

Synthesis of Compound 8 under an argon atmosphere (27.0 ppm O_2).
Dianionic $\mathbf{2}^{2.5-}$ was electrochemically obtained from **2** (8.9 mg, 0.010 mmol) by CPE at –1.34 V vs. SCE for ca. 1 h and then stirred at 25 °C for 5 h, followed by treatment with TFA (3.8 μL, 0.050 mmol) for 10 min. All operations were performed under an argon atmosphere (27.0 ppm O_2), and the gas flow rate was 2.6 mL s^{-1}. The resulting mixture was filtered and then purified on a silica gel column (300–400 mesh) with CS_2 as the eluent to give product **8** (2.7 mg, 30%).

Synthesis of Compound 8 with a gas flow rate of 1.3 mL s^{-1}.
Dianionic $\mathbf{2}^{2.5-}$ was electrochemically obtained from **2** (9.0 mg, 0.010 mmol) by CPE at –1.34 V vs. SCE for ca. 1 h and then stirred at 25 °C for 5 h, followed by treatment with TFA (3.8 μL, 0.050 mmol) for 10 min. All operations were performed under an argon atmosphere (15.3 ppm O_2), and the gas flow rate was 1.3 mL s^{-1}. The resulting mixture was filtered and then purified on a silica gel column (300–400 mesh) with CS_2 as the eluent to give product **8** (2.9 mg, 32%).

4. Cyclic Voltammograms of 1–8 and Differential Pulse Voltammograms of 3–8

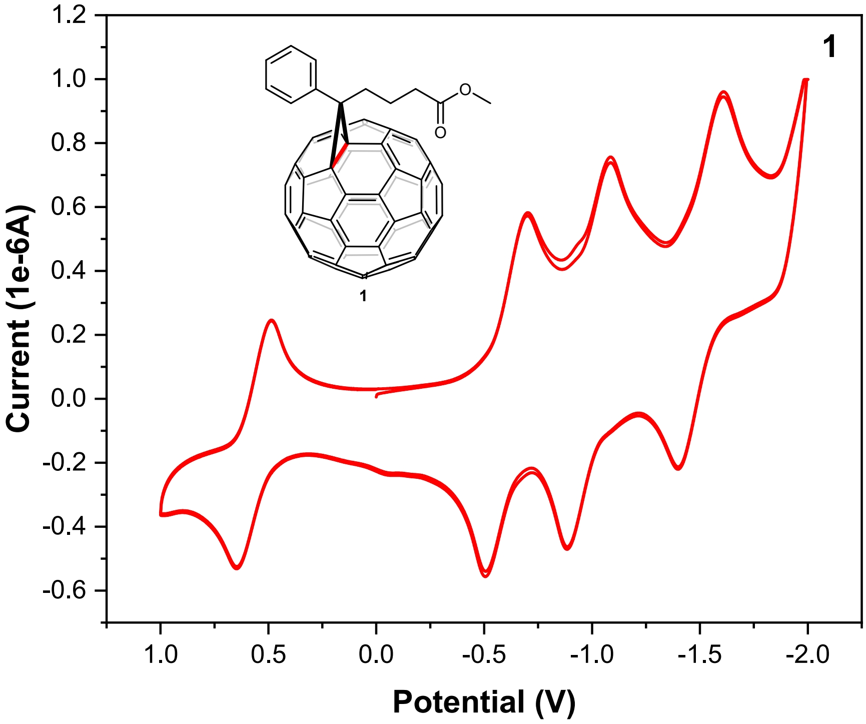

Supplementary Fig. 1. Cyclic voltammogram of compound 1. Votage versus ferrocene/ferrocenium; reference electrode: saturated calomel electrode; working electrode: Pt disc; auxiliary electrode: Pt wire; scanning rate: 50 mV s^{-1}.

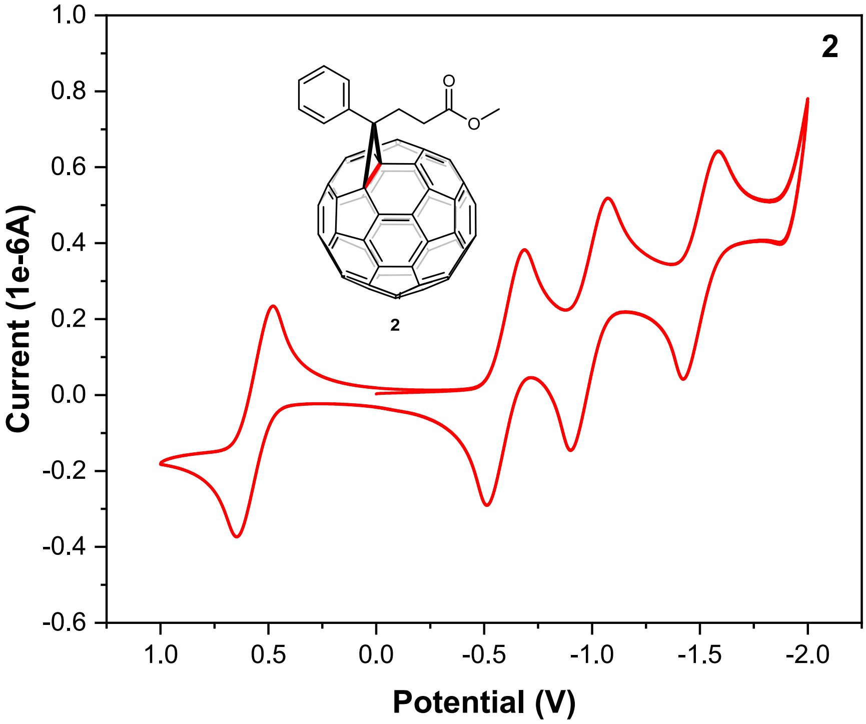

Supplementary Fig. 2. Cyclic voltammogram of compound 2. Votage versus ferrocene/ferrocenium; reference electrode: saturated calomel electrode; working electrode: Pt disc; auxiliary electrode: Pt wire; scanning rate: 50 mV s^{-1}.

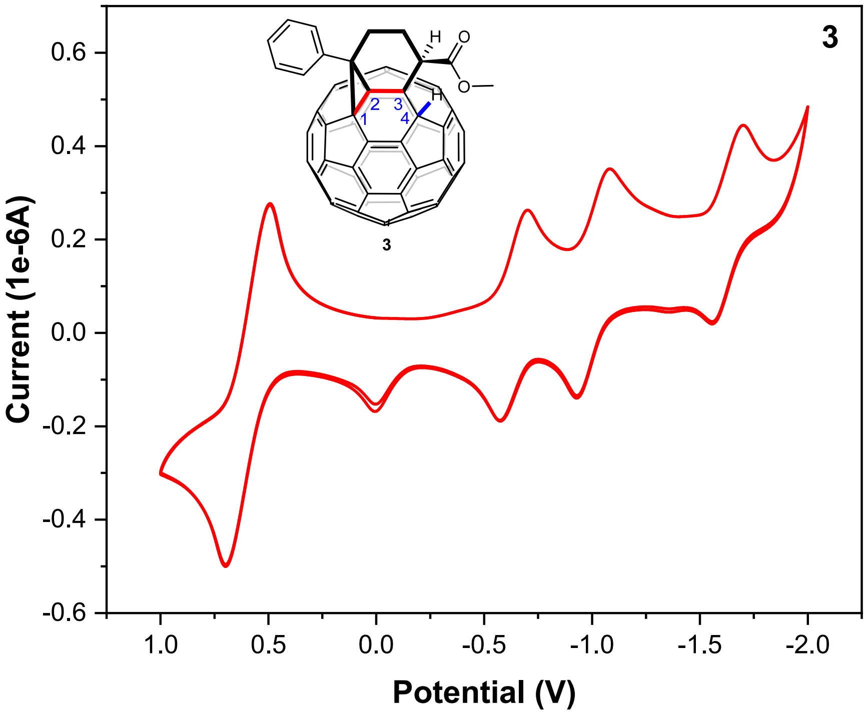

Supplementary Fig. 3. Cyclic voltammogram of compound 3. Votage versus ferrocene/ferrocenium; reference electrode: saturated calomel electrode; working electrode: Pt disc; auxiliary electrode: Pt wire; scanning rate: 50 mV s^{-1}.

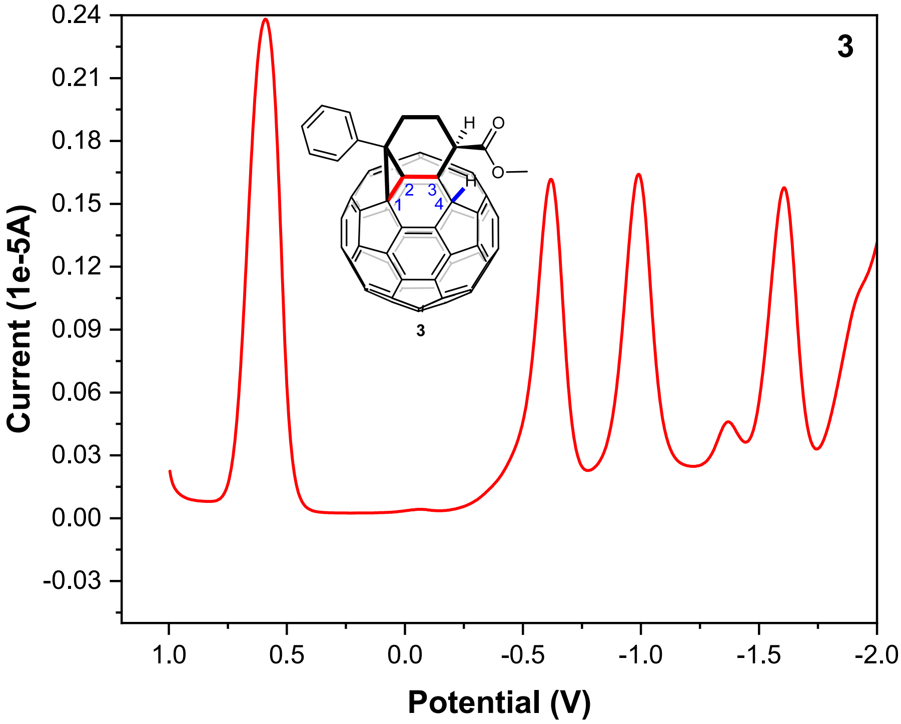

Supplementary Fig. 4. Differential pulse voltammogram of compound 3. Votage versus ferrocene/ferrocenium; reference electrode: saturated calomel electrode; working electrode: Pt disc; auxiliary electrode: Pt wire; scanning rate: 50 mV s^{-1}.

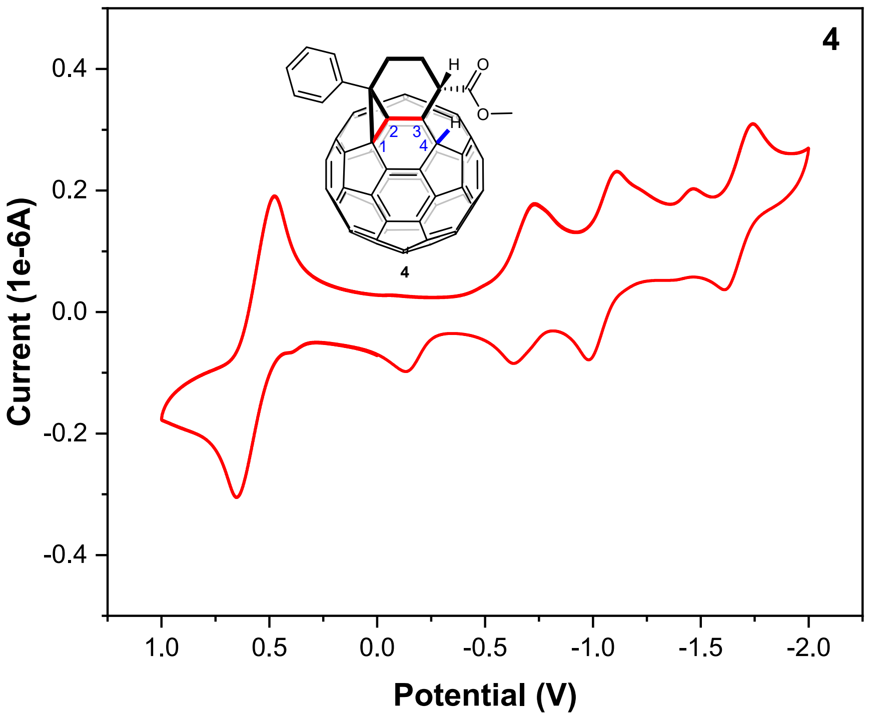

Supplementary Fig. 5. Cyclic voltammogram of compound 4. Votage versus ferrocene/ferrocenium; reference electrode: saturated calomel electrode; working electrode: Pt disc; auxiliary electrode: Pt wire; scanning rate: 50 mV s^{-1}.

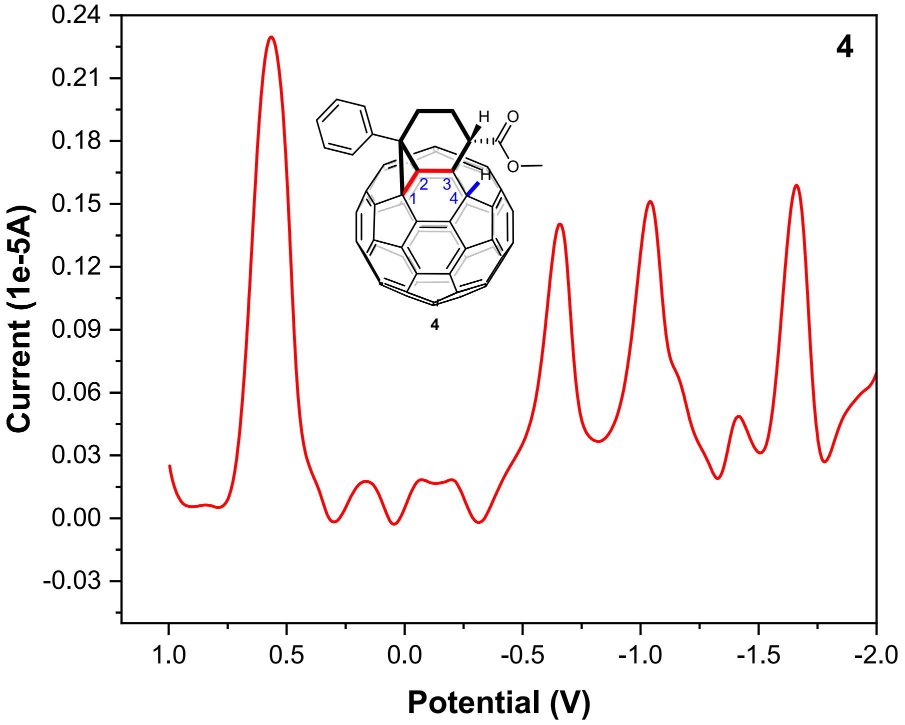

Supplementary Fig. 6. Differential pulse voltammogram of compound 4. Votage versus ferrocene/ferrocenium; reference electrode: saturated calomel electrode; working electrode: Pt disc; auxiliary electrode: Pt wire; scanning rate: 50 mV s^{-1}.

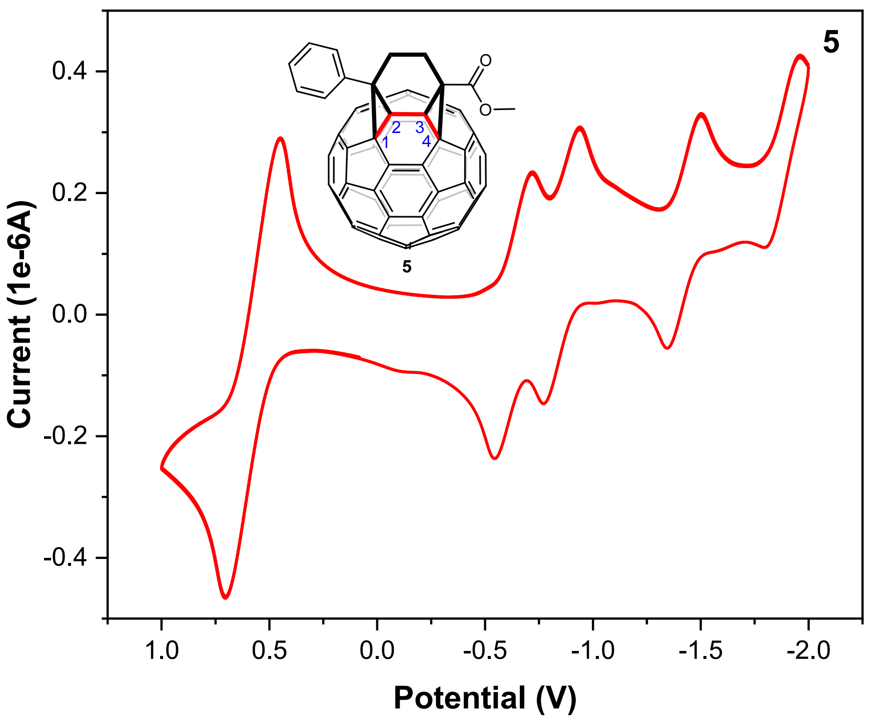

Supplementary Fig. 7. Cyclic voltammogram of compound 5. Votage versus ferrocene/ferrocenium; reference electrode: saturated calomel electrode; working electrode: Pt disc; auxiliary electrode: Pt wire; scanning rate: 50 mV s^{-1}.

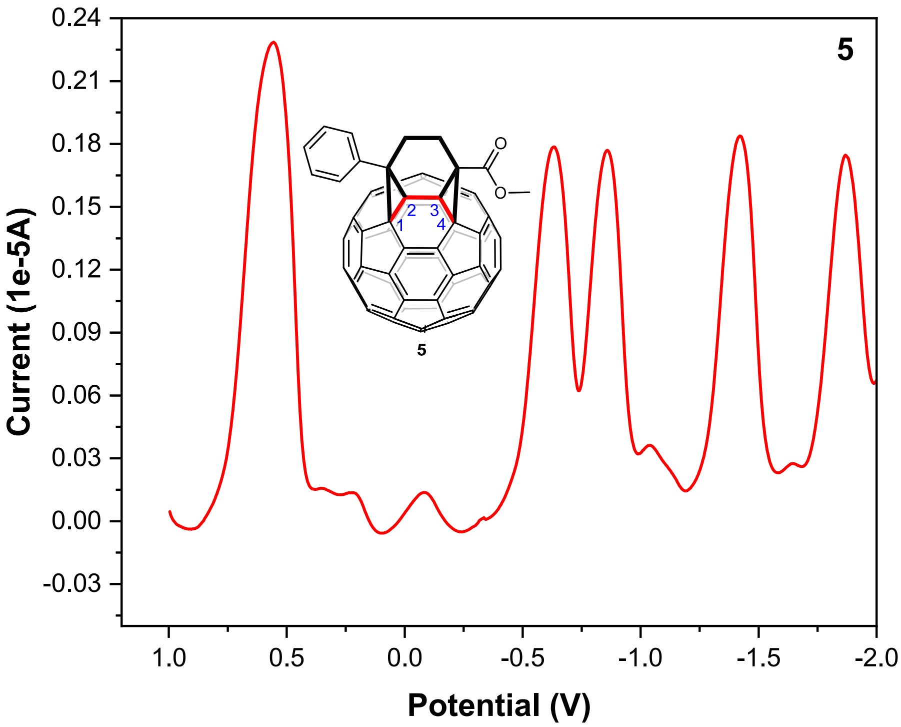

Supplementary Fig. 8. Differential pulse voltammogram of compound 5. Votage versus ferrocene/ferrocenium; reference electrode: saturated calomel electrode; working electrode: Pt disc; auxiliary electrode: Pt wire; scanning rate: 50 mV s^{-1}.

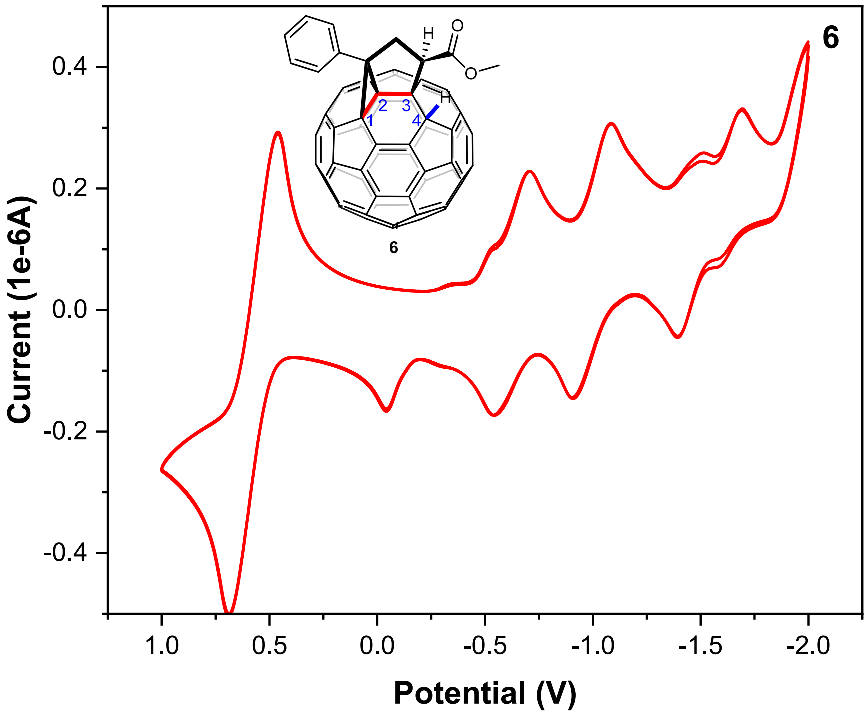

Supplementary Fig. 9. Cyclic voltammogram of compound 6. Votage versus ferrocene/ferrocenium; reference electrode: saturated calomel electrode; working electrode: Pt disc; auxiliary electrode: Pt wire; scanning rate: 50 mV s^{-1}.

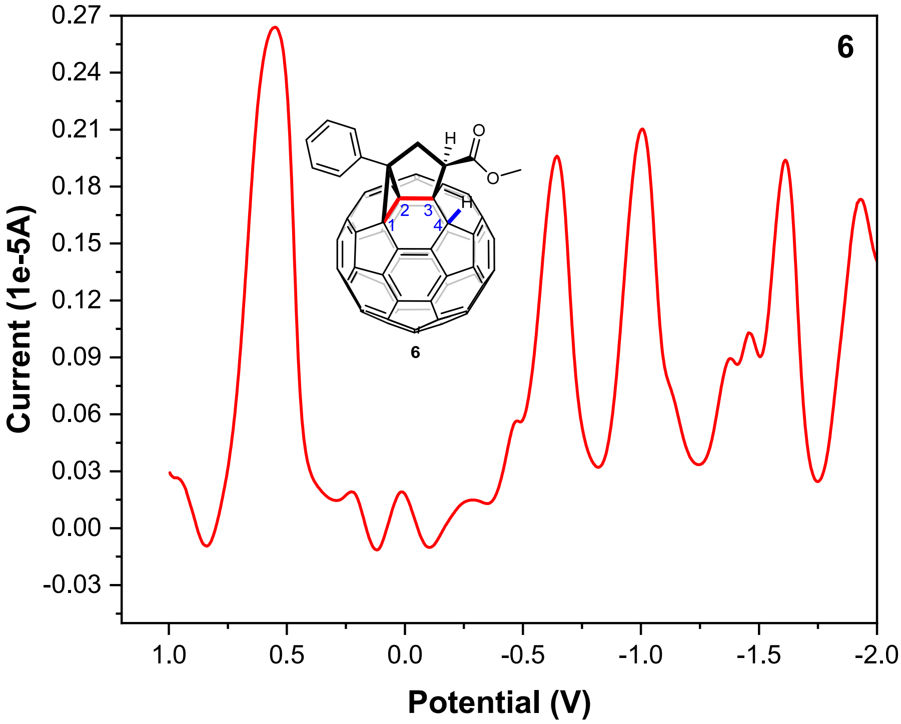

Supplementary Fig. 10. Differential pulse voltammogram of compound 6. Votage versus ferrocene/ferrocenium; reference electrode: saturated calomel electrode; working electrode: Pt disc; auxiliary electrode: Pt wire; scanning rate: 50 mV s^{-1}.

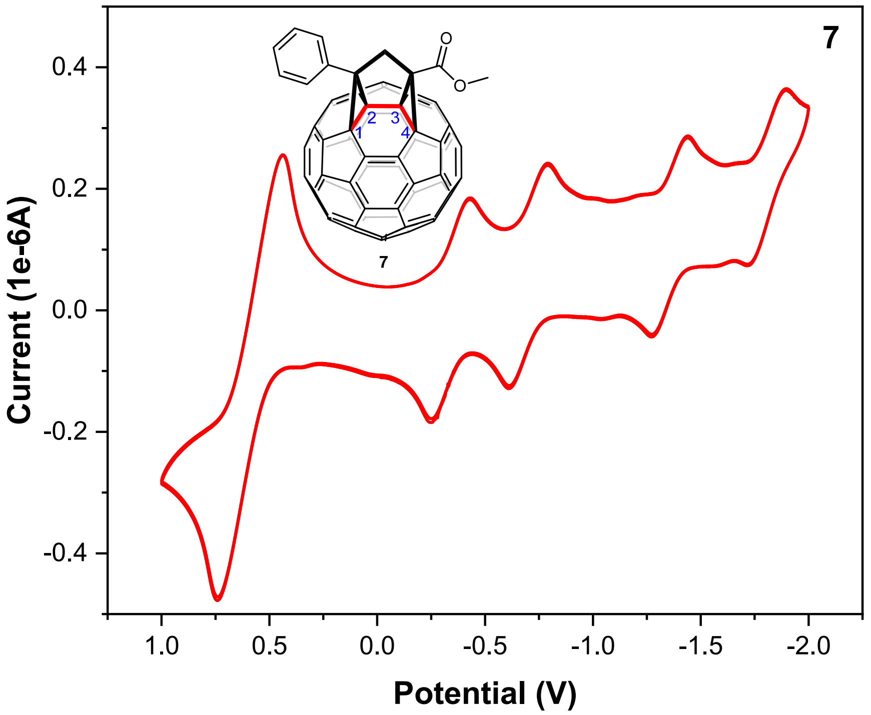

Supplementary Fig. 11. Cyclic voltammogram of compound 7. Votage versus ferrocene/ferrocenium; reference electrode: saturated calomel electrode; working electrode: Pt disc; auxiliary electrode: Pt wire; scanning rate: 50 mV s^{-1}.

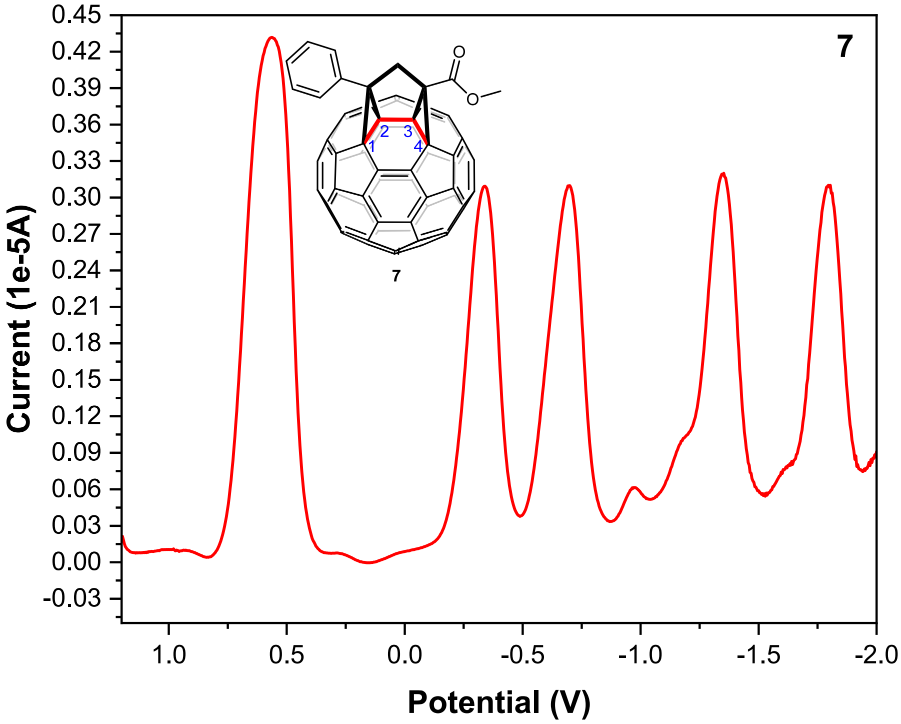

Supplementary Fig. 12. Differential pulse voltammogram of compound 7. Votage versus ferrocene/ferrocenium; reference electrode: saturated calomel electrode; working electrode: Pt disc; auxiliary electrode: Pt wire; scanning rate: 50 mV s^{-1}.

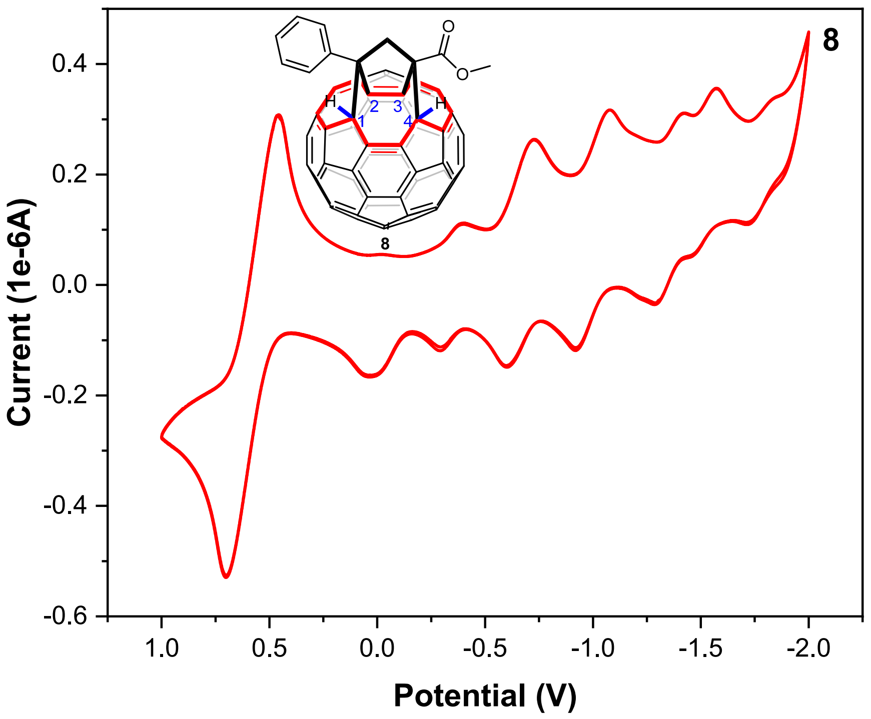

Supplementary Fig. 13. Cyclic voltammogram of compound 8. Votage versus ferrocene/ferrocenium; reference electrode: saturated calomel electrode; working electrode: Pt disc; auxiliary electrode: Pt wire; scanning rate: 50 mV s^{-1}.

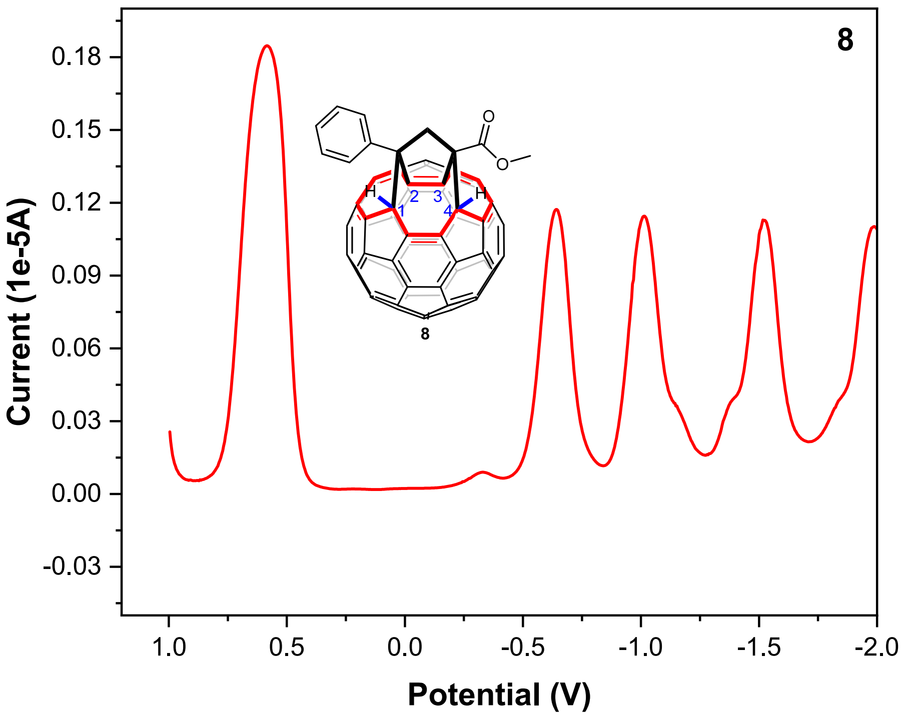

Supplementary Fig. 14. Differential pulse voltammogram of compound 8. Votage versus ferrocene/ferrocenium; reference electrode: saturated calomel electrode; working electrode: Pt disc; auxiliary electrode: Pt wire; scanning rate: 50 mV s^{-1}.

5. UV-vis Spectra of 3–8

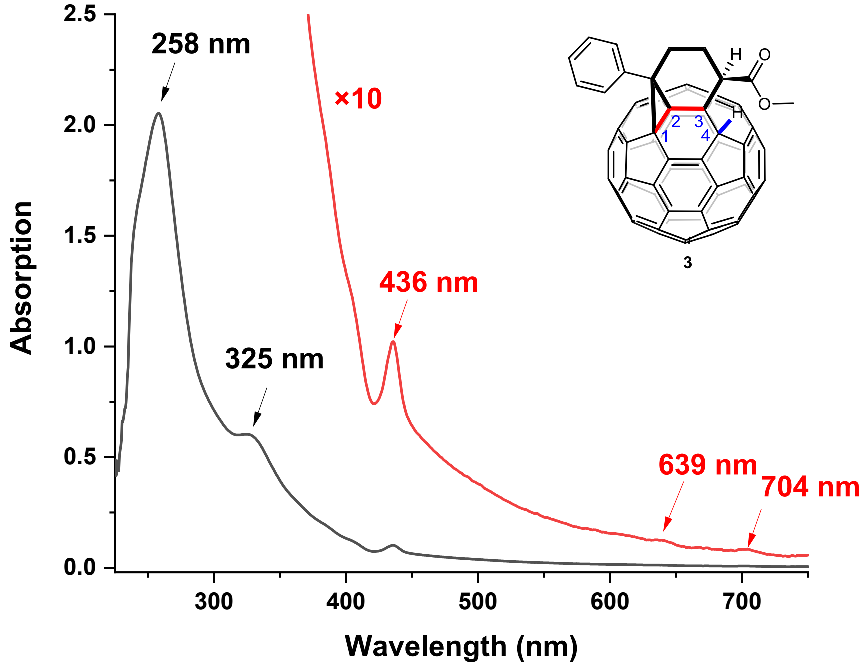

Supplementary Fig. 15. UV-vis absorption of compound 3 in $CHCl_3$.

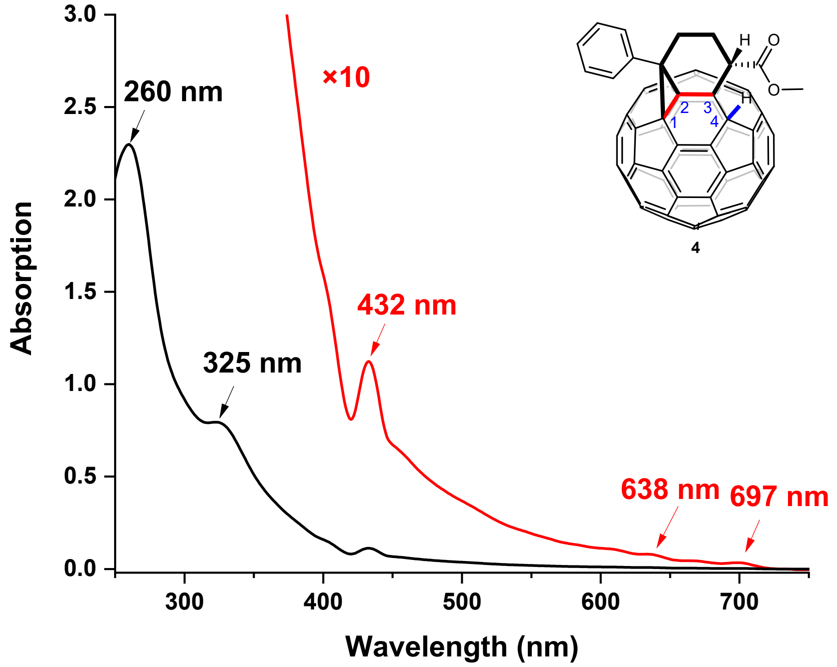

Supplementary Fig. 16. UV-vis absorption of compound 4 in $CHCl_3$.

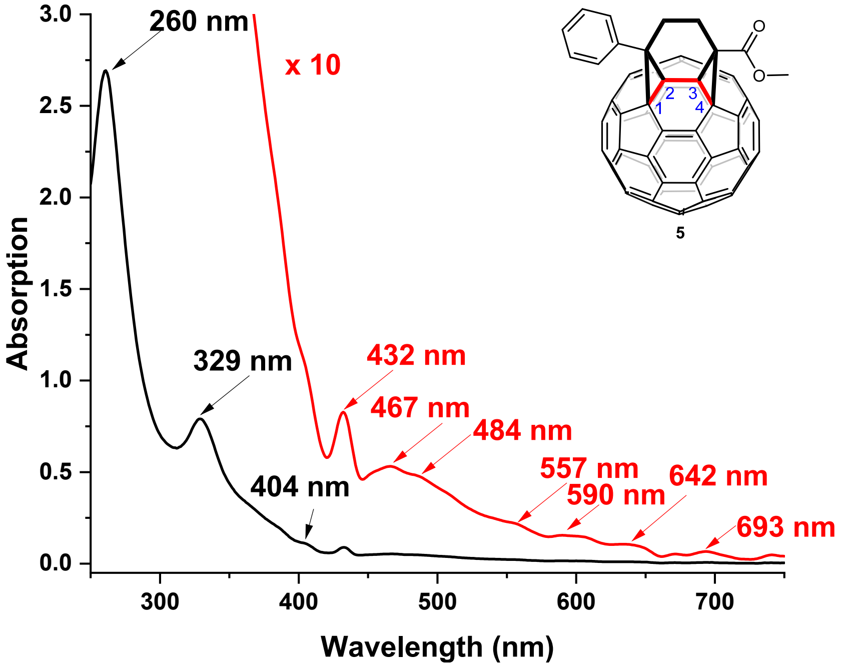

Supplementary Fig. 17. UV-vis absorption of compound 5 in $CHCl_3$.

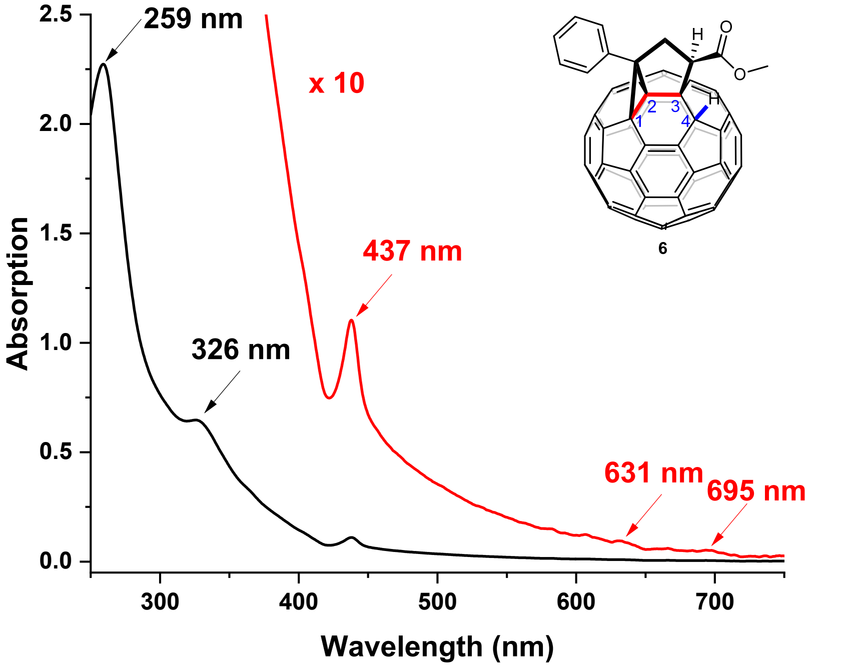

Supplementary Fig. 18. UV-vis absorption of compound 6 in $CHCl_3$.

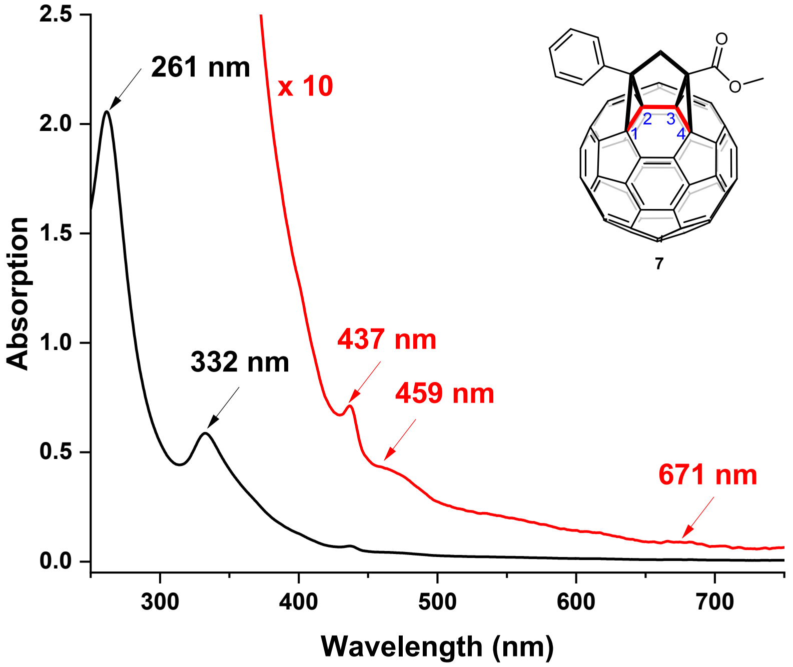

Supplementary Fig. 19. UV-vis absorption of compound 7 in $CHCl_3$.

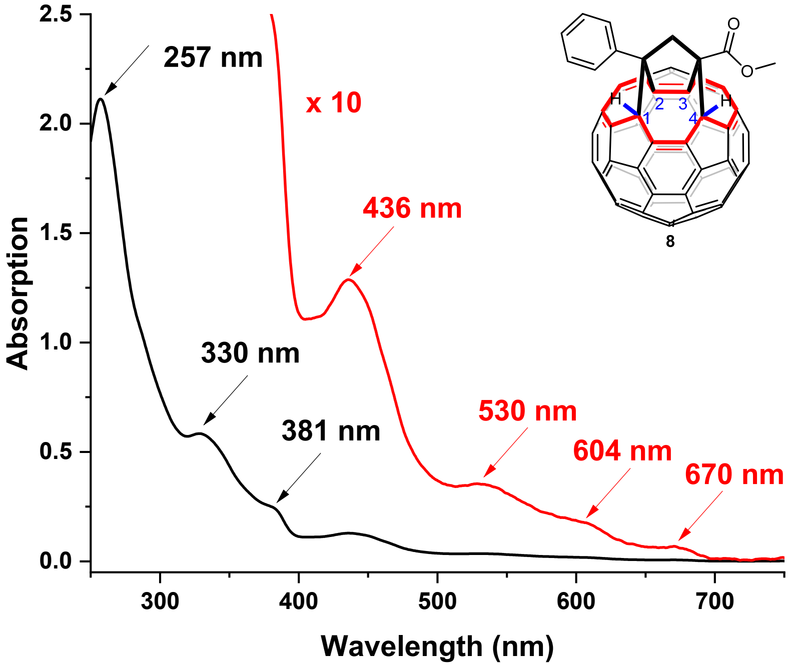

Supplementary Fig. 20. UV-vis absorption of compound 8 in $CHCl_3$.

6. NMR Spectra of 1–8

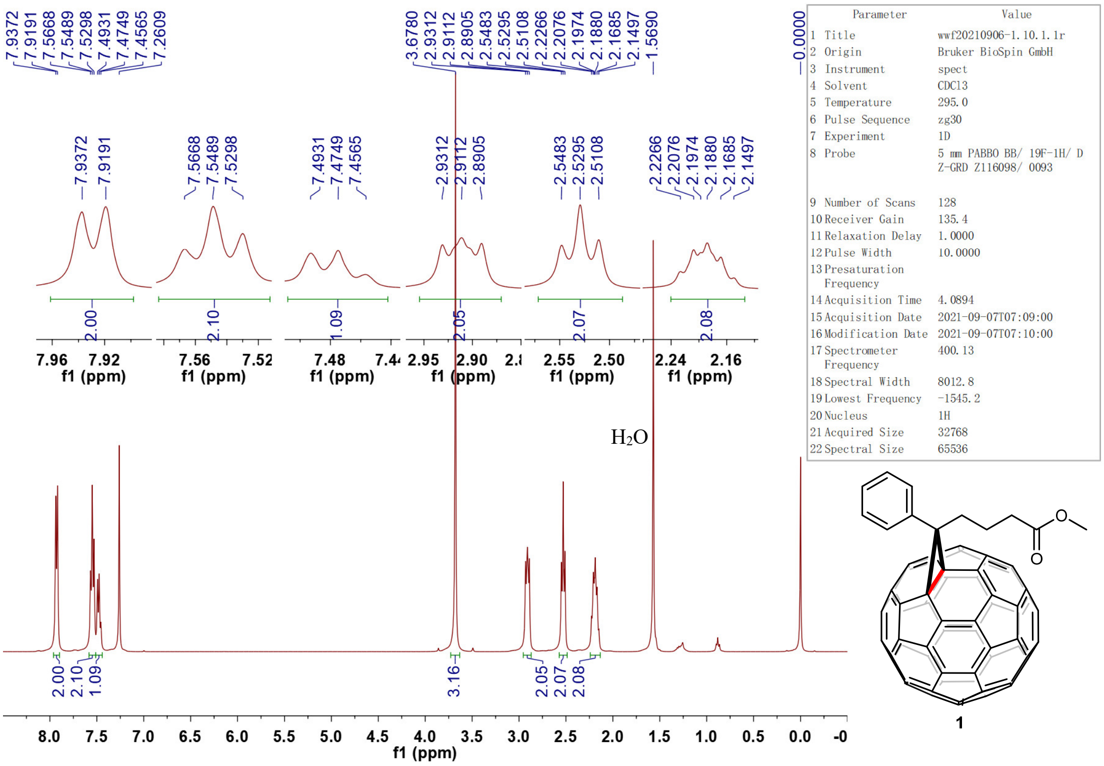

Supplementary Fig. 21. ^{1}H NMR (400 MHz, $CDCl_3$) of 1.

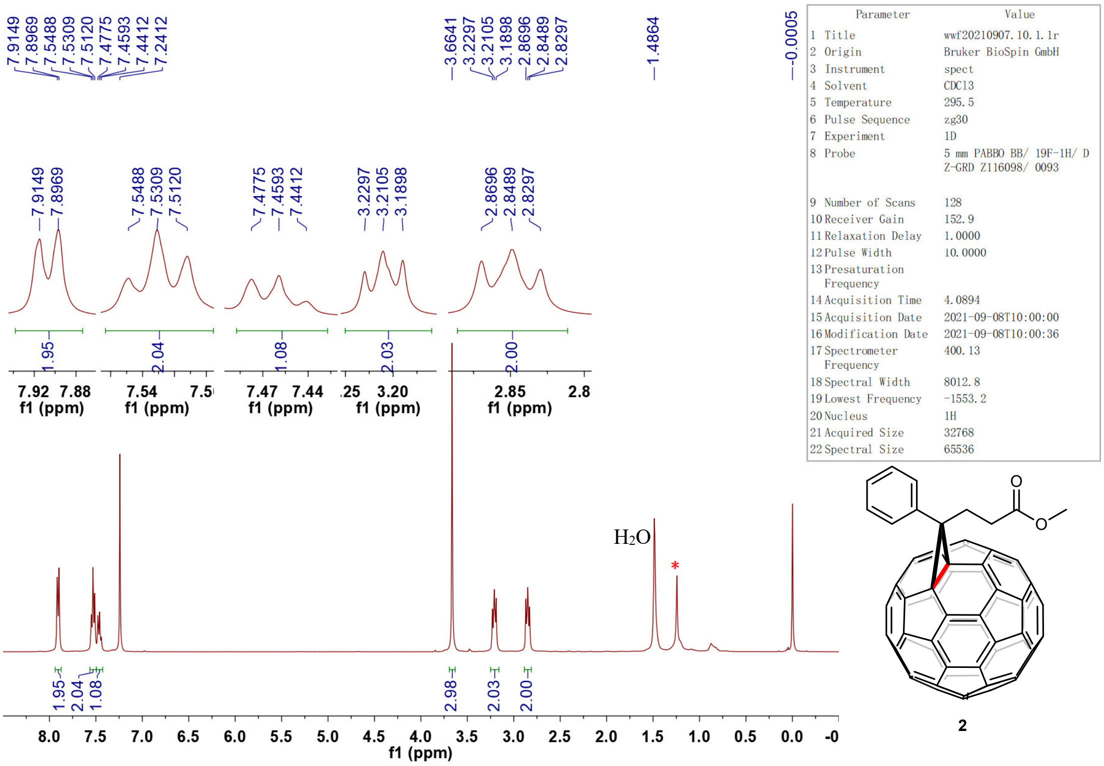

Supplementary Fig. 22. ^{1}H NMR (400 MHz, 1:1 $CS_2/CDCl_3$) of 2. The symbol * represents the peak of petroleum ether.

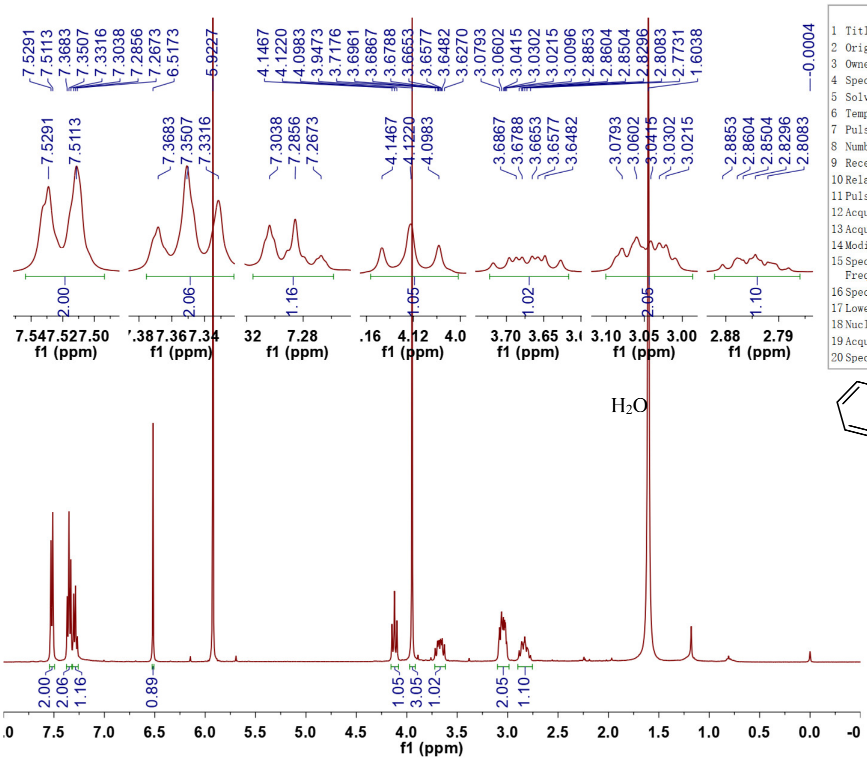

Parameter	Value
1 Title	wwf20200816
2 Origin	Bruker BioSpin GmbH
3 Owner	nmrsu
4 Spectrometer	spect
5 Solvent	CDCl2CDCl2
6 Temperature	296.5
7 Pulse Sequence	zg30
8 Number of Scans	128
9 Receiver Gain	153
10 Relaxation Delay	1.0000
11 Pulse Width	10.0000
12 Acquisition Time	4.0894
13 Acquisition Date	2020-08-16T07:42:22
14 Modification Date	2020-08-16T07:42:24
15 Spectrometer Frequency	400.13
16 Spectral Width	8012.8
17 Lowest Frequency	-1312.2
18 Nucleus	1H
19 Acquired Size	32768
20 Spectral Size	65536

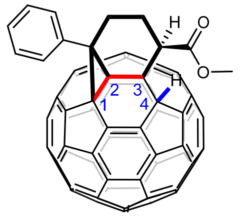

3

Supplementary Fig. 23. ^{1}H NMR (400 MHz, $C_2D_2Cl_4$) of 3.

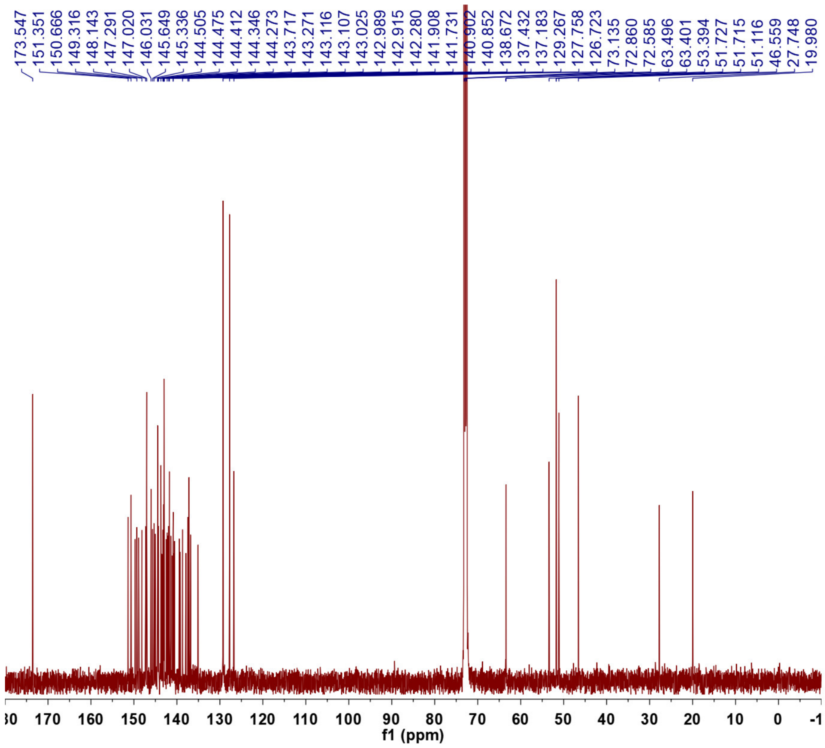

Parameter	Value
1 Title	wwf20200816
2 Origin	Bruker BioSpin GmbH
3 Owner	nmrsu
4 Spectrometer	spect
5 Solvent	CDCl2CDCl2
6 Temperature	297.1
7 Pulse Sequence	zgpg30
8 Number of Scans	12000
9 Receiver Gain	212
10 Relaxation Delay	2.0000
11 Pulse Width	10.0000
12 Acquisition Time	1.3631
13 Acquisition Date	2020-08-16T19:08:40
14 Modification Date	2020-08-16T19:08:42
15 Spectrometer Frequency	100.61
16 Spectral Width	24038.5
17 Lowest Frequency	-2009.9
18 Nucleus	13C
19 Acquired Size	32768
20 Spectral Size	32768

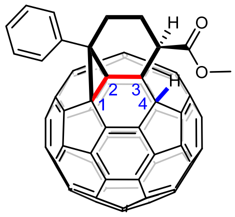

3

Supplementary Fig. 24. ^{13}C NMR (101 MHz, $C_2D_2Cl_4$) of 3.

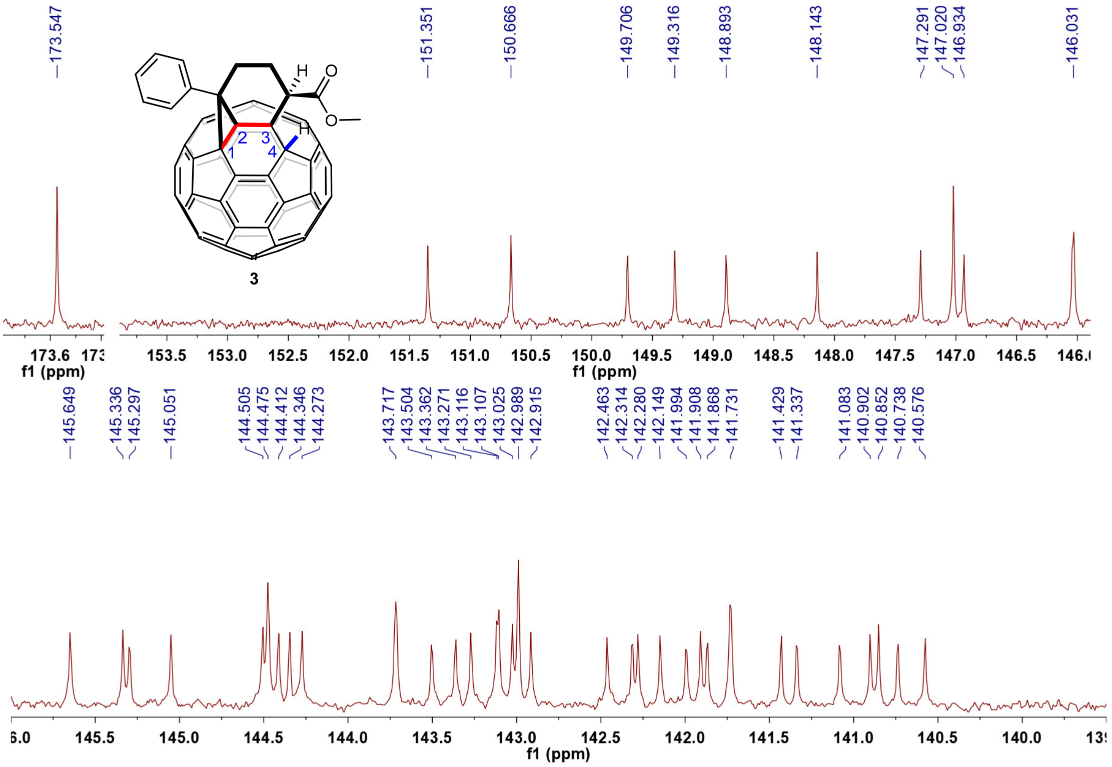

Supplementary Fig. 25. Expanded ^{13}C NMR (101 MHz, $C_2D_2Cl_4$) of 3.

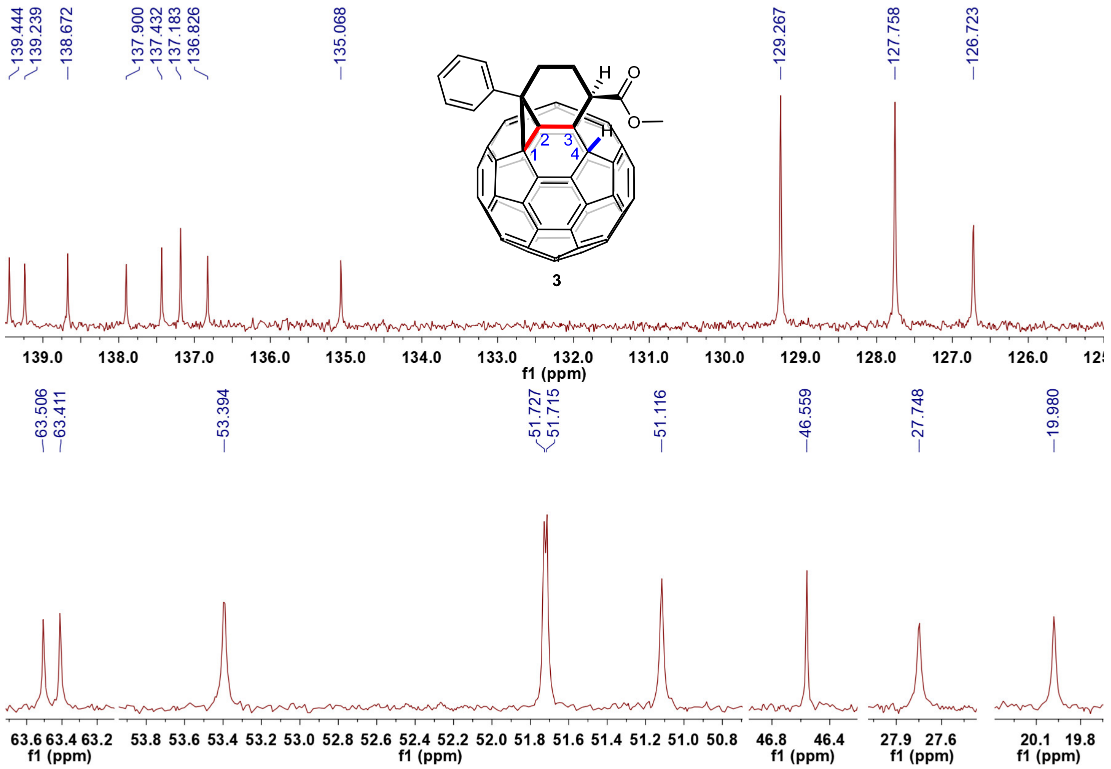

Supplementary Fig. 26. Expanded ^{13}C NMR (101 MHz, $C_2D_2Cl_4$) of 3.

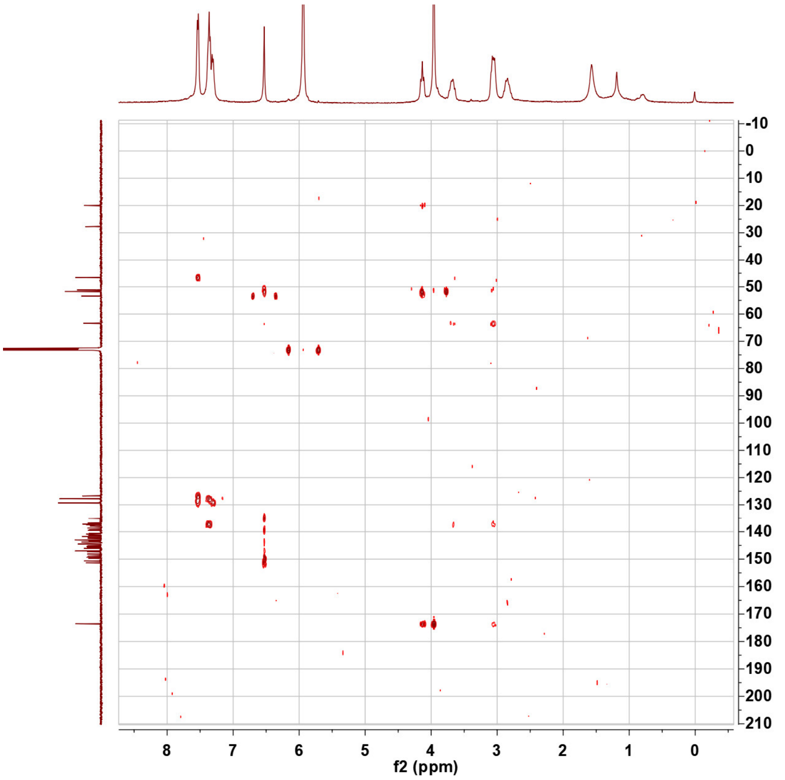

	Parameter	Value
1	Title	wwf20230221-ePCBM. 16.1.2rr
2	Origin	Bruker BioSpin GmbH
3	Instrument	spect
4	Solvent	CDCl2CDCl2
5	Temperature	291.8
6	Pulse Sequence	hmbcgpndqf
7	Experiment	HMBC
8	Probe	5 mm PABBO BB/ 19F-1H/ D Z-GRD Z116098/ 0092
9	Number of Scans	16
10	Receiver Gain	211.9
11	Relaxation Delay	1.3353
12	Pulse Width	10.0000
13	Presaturation Frequency	
14	Acquisition Time	0.5489
15	Acquisition Date	2023-02-22T06:11:00
16	Modification Date	2023-02-22T07:17:52
17	Spectrometer Frequency	(400.13, 100.61)
18	Spectral Width	(3731.3, 22321.4)
19	Lowest Frequency	(-243.3, -1188.0)
20	Nucleus	(1H, 13C)
21	Acquired Size	(2048, 128)
22	Spectral Size	(1024, 1024)

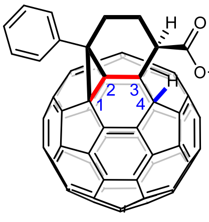

3

Supplementary Fig. 27. HMBC (400/101 MHz, $C_2D_2Cl_4$) of 3.

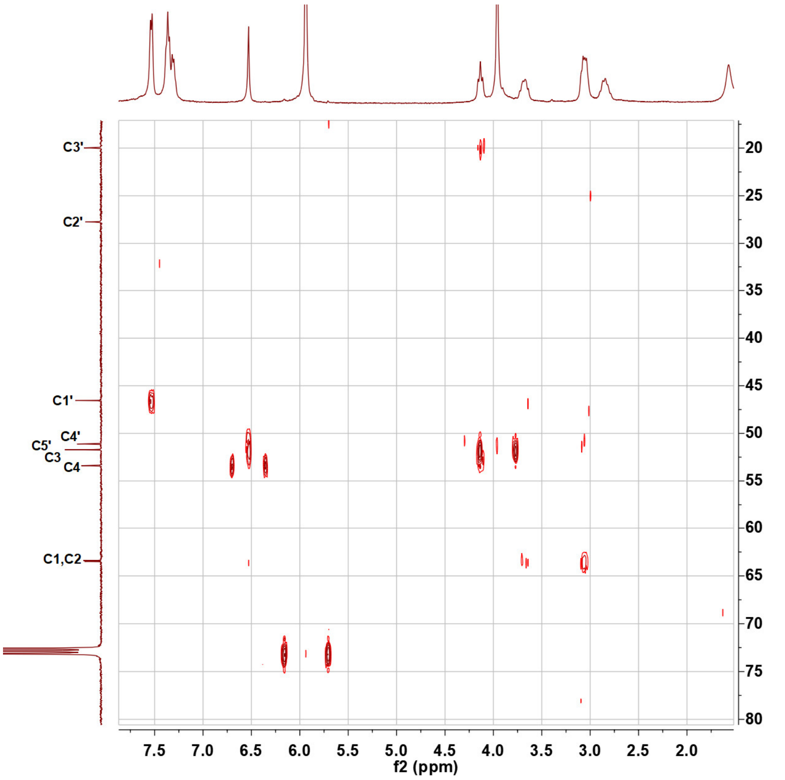

	Parameter	Value
1	Title	wwf20230221-ePCBM. 16.1.2rr
2	Origin	Bruker BioSpin GmbH
3	Instrument	spect
4	Solvent	CDCl2CDCl2
5	Temperature	291.8
6	Pulse Sequence	hmbcgpndqf
7	Experiment	HMBC
8	Probe	5 mm PABBO BB/ 19F-1H/ D Z-GRD Z116098/ 0092
9	Number of Scans	16
10	Receiver Gain	211.9
11	Relaxation Delay	1.3353
12	Pulse Width	10.0000
13	Presaturation Frequency	
14	Acquisition Time	0.5489
15	Acquisition Date	2023-02-22T06:11:00
16	Modification Date	2023-02-22T07:17:52
17	Spectrometer Frequency	(400.13, 100.61)
18	Spectral Width	(3731.3, 22321.4)
19	Lowest Frequency	(-243.3, -1188.0)
20	Nucleus	(1H, 13C)
21	Acquired Size	(2048, 128)
22	Spectral Size	(1024, 1024)

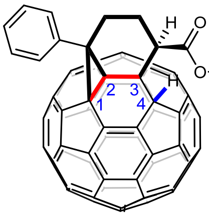

3

Supplementary Fig. 28. Expanded HMBC (400/101 MHz, $C_2D_2Cl_4$) of 3.

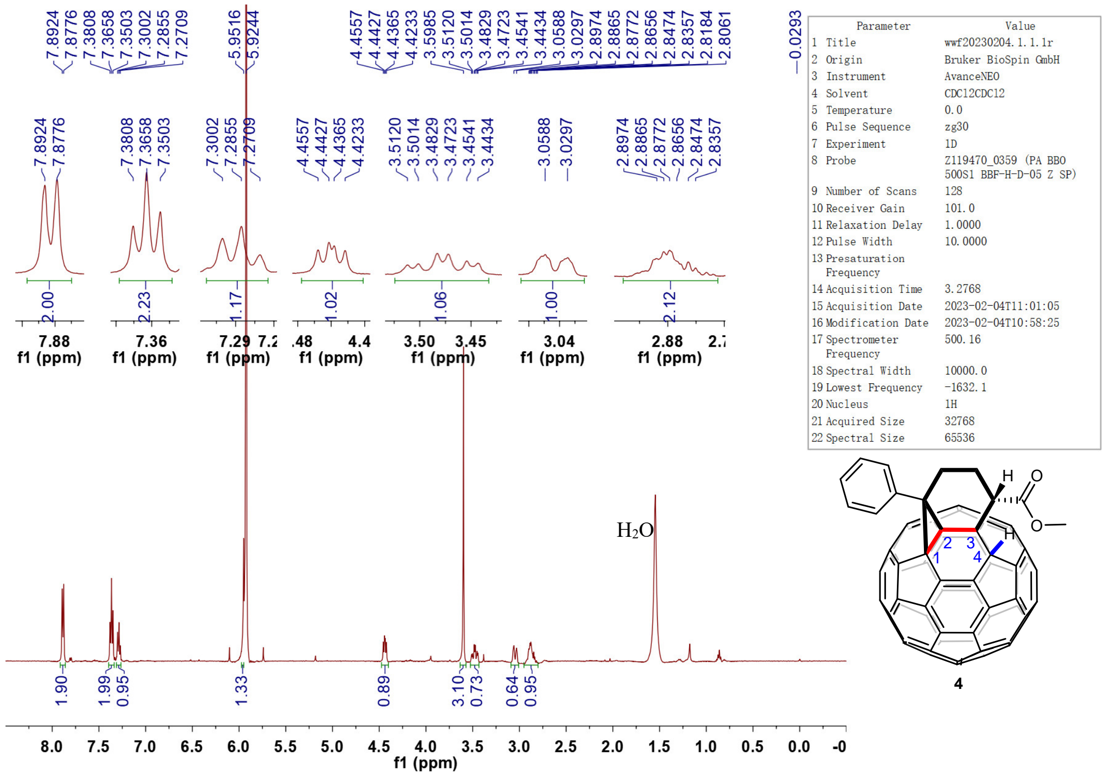

Supplementary Fig. 29. ^{1}H NMR (500 MHz, $C_2D_2Cl_4$) of 4.

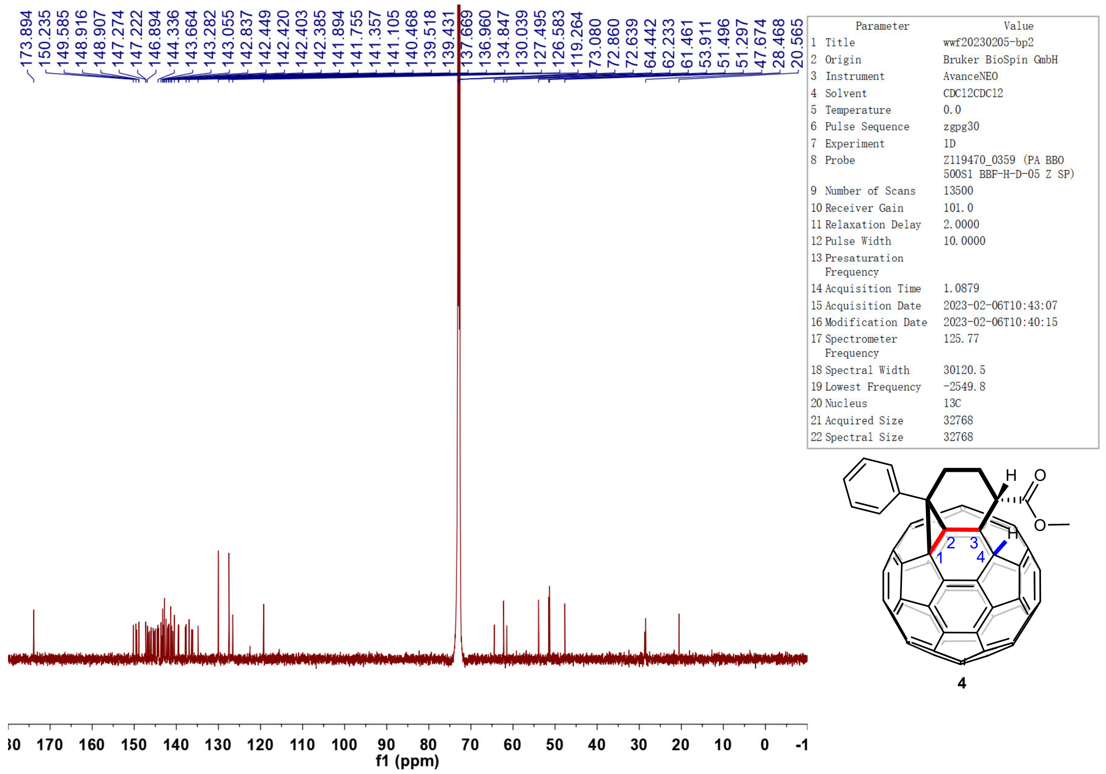

Supplementary Fig. 30. ^{13}C NMR (126 MHz, $C_2D_2Cl_4$) of 4.

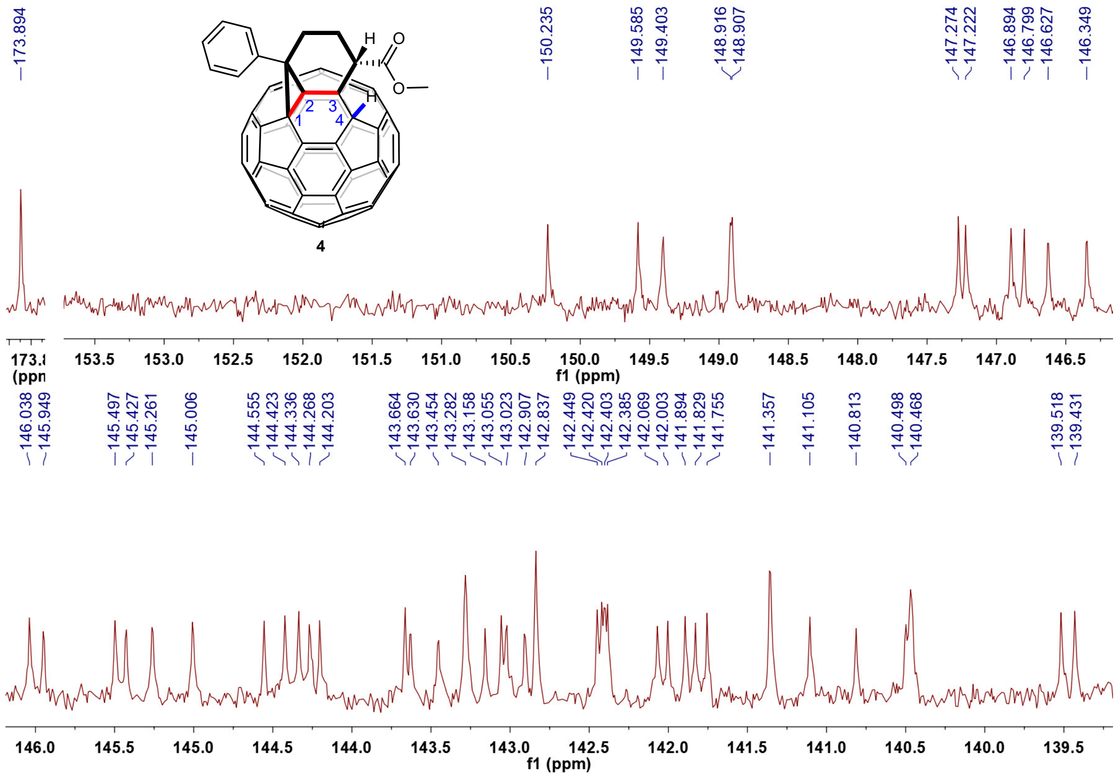

Supplementary Fig. 31. Expanded ^{13}C NMR (126 MHz, $C_2D_2Cl_4$) of 4.

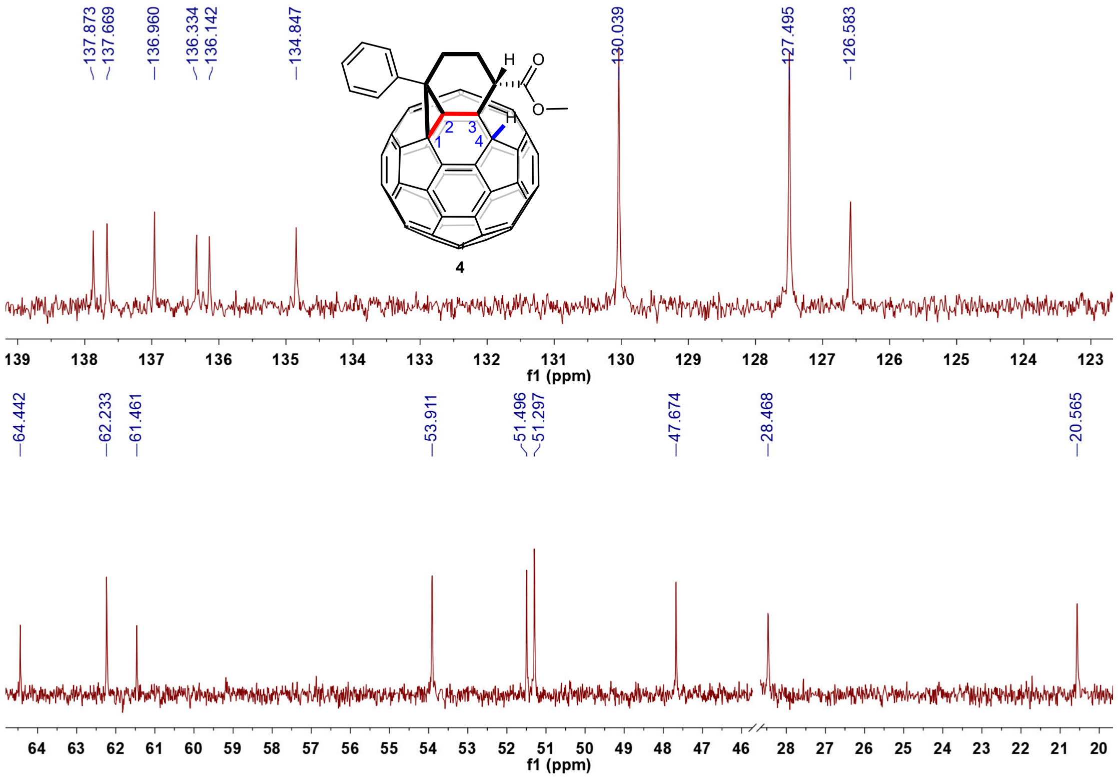

Supplementary Fig. 32. Expanded ^{13}C NMR (126 MHz, $C_2D_2Cl_4$) of 4.

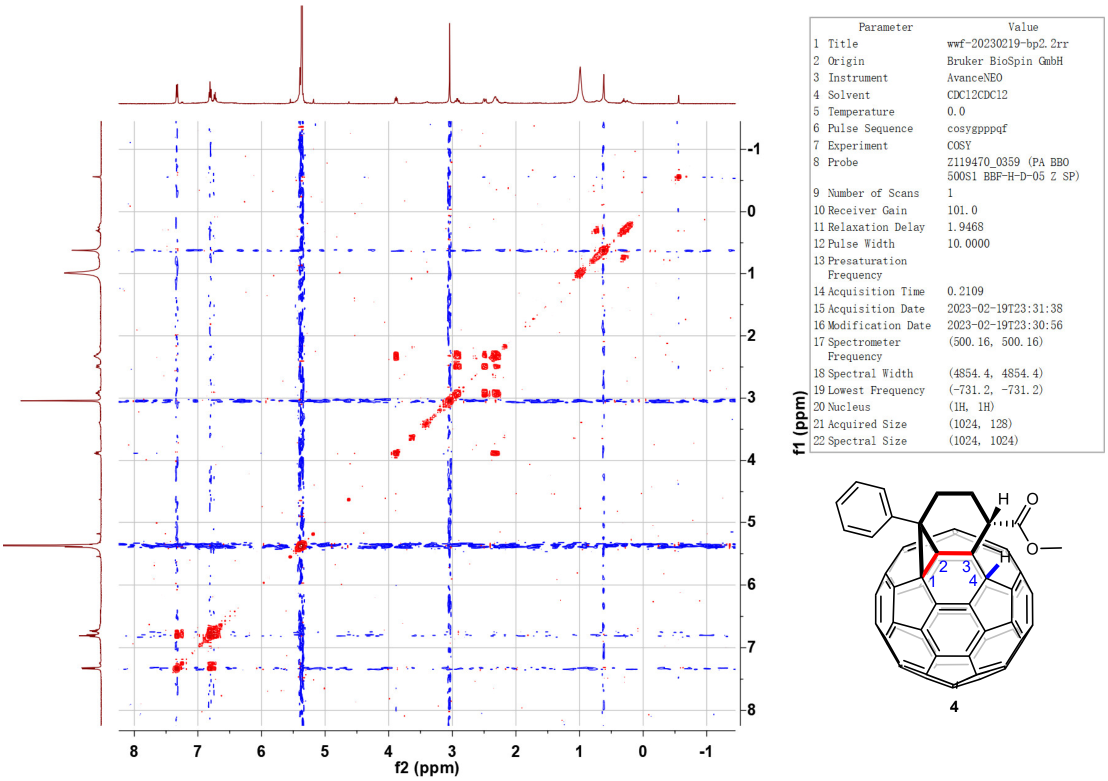

Parameter	Value
1 Title	wwf-20230219-bp2.2rr
2 Origin	Bruker BioSpin GmbH
3 Instrument	AvanceNEO
4 Solvent	CDCl2CDCl2
5 Temperature	0.0
6 Pulse Sequence	cosygpppqf
7 Experiment	COSY
8 Probe	Z119470_0359 (PA BBO 500S1 BBF-H-D-05 Z SP)
9 Number of Scans	1
10 Receiver Gain	101.0
11 Relaxation Delay	1.9468
12 Pulse Width	10.0000
13 Presaturation Frequency	
14 Acquisition Time	0.2109
15 Acquisition Date	2023-02-19T23:31:38
16 Modification Date	2023-02-19T23:30:56
17 Spectrometer Frequency	(500.16, 500.16)
18 Spectral Width	(4854.4, 4854.4)
19 Lowest Frequency	(-731.2, -731.2)
20 Nucleus	(1H, 1H)
21 Acquired Size	(1024, 128)
22 Spectral Size	(1024, 1024)

4

Supplementary Fig. 33. ^{1}H,^{1}H-COSY (500 MHz, $C_2D_2Cl_4$) of 4.

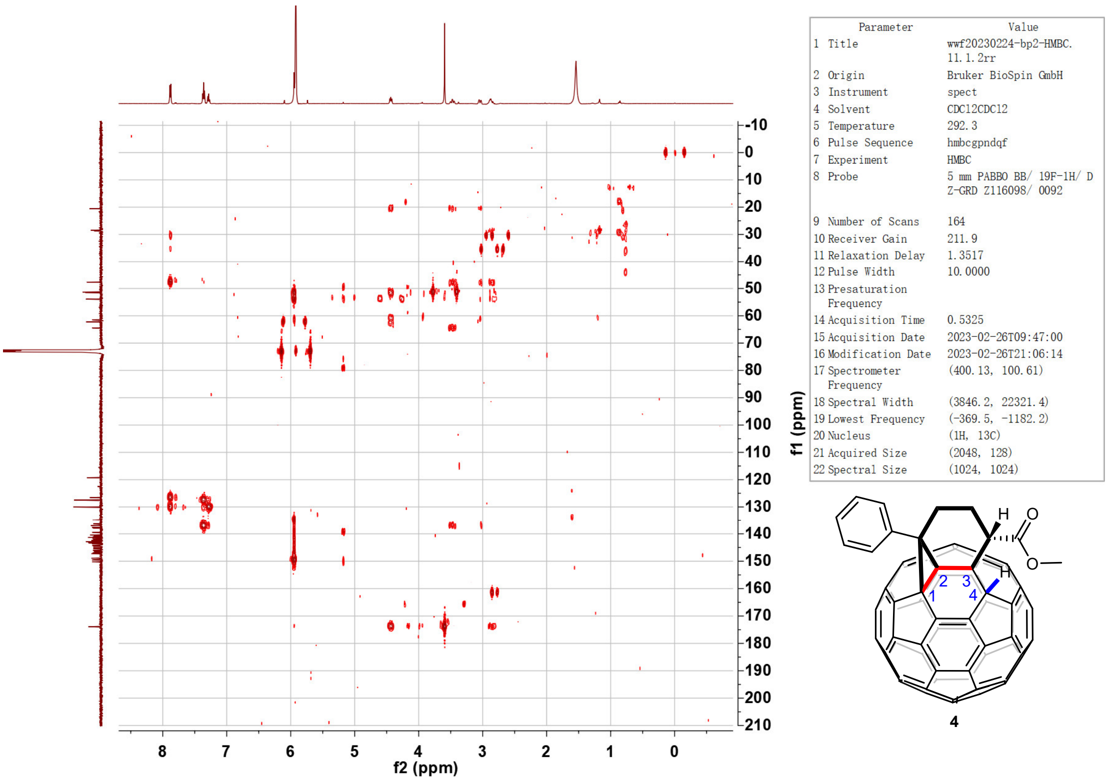

Parameter	Value
1 Title	wwf20230224-bp2-HMBC.11.1.2rr
2 Origin	Bruker BioSpin GmbH
3 Instrument	spect
4 Solvent	CDCl2CDCl2
5 Temperature	292.3
6 Pulse Sequence	hmbcgpndqf
7 Experiment	HMBC
8 Probe	5 mm PABBO BB/ 19F-1H/ DZ-GRD Z116098/ 0092
9 Number of Scans	164
10 Receiver Gain	211.9
11 Relaxation Delay	1.3517
12 Pulse Width	10.0000
13 Presaturation Frequency	
14 Acquisition Time	0.5325
15 Acquisition Date	2023-02-26T09:47:00
16 Modification Date	2023-02-26T21:06:14
17 Spectrometer Frequency	(400.13, 100.61)
18 Spectral Width	(3846.2, 22321.4)
19 Lowest Frequency	(-369.5, -1182.2)
20 Nucleus	(1H, 13C)
21 Acquired Size	(2048, 128)
22 Spectral Size	(1024, 1024)

4

Supplementary Fig. 34. HMBC (400/101 MHz, $C_2D_2Cl_4$) of 4.

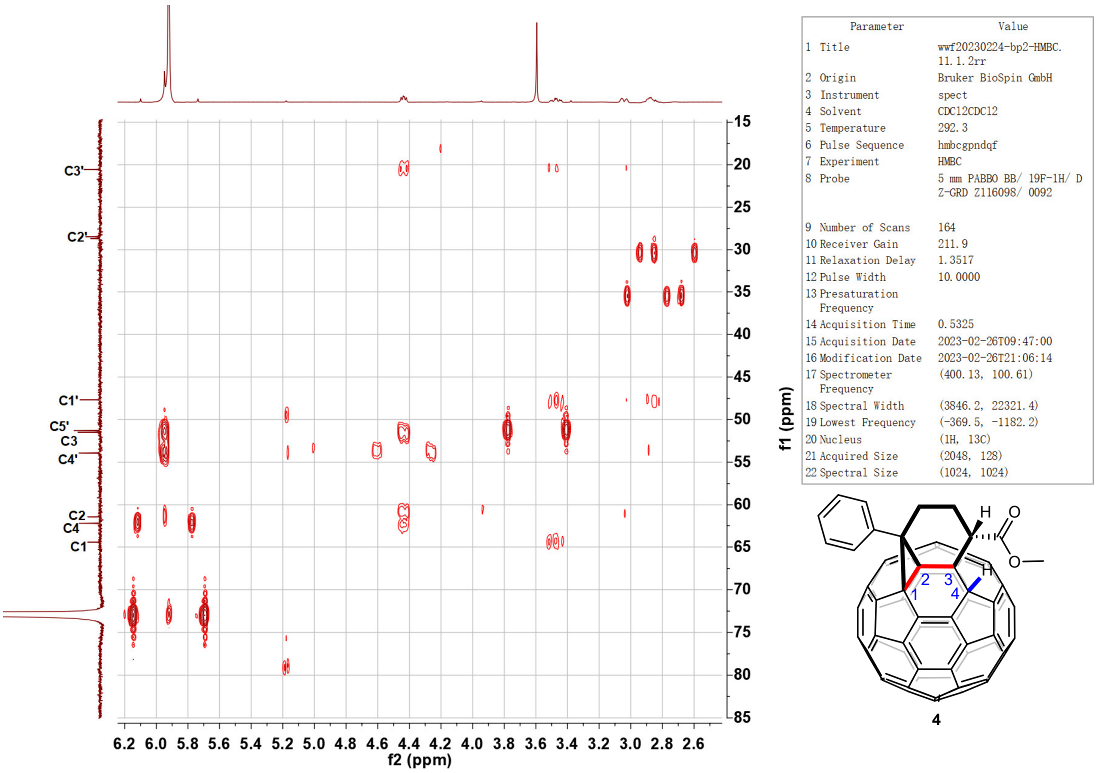

Supplementary Fig. 35. Expanded HMBC (400/101 MHz, $C_2D_2Cl_4$) of 4.

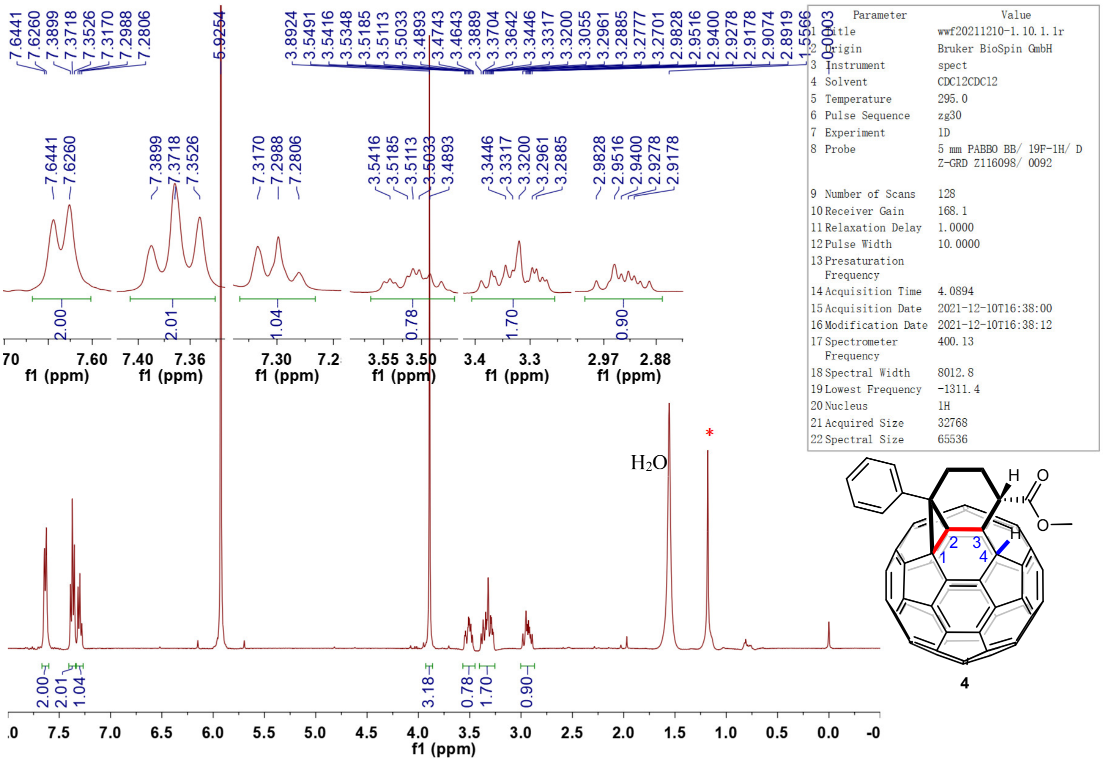

Supplementary Fig. 36. ^{1}H NMR (400 MHz, $C_2D_2Cl_4$) of 5. The symbol * represents the peak of petroleum ether.

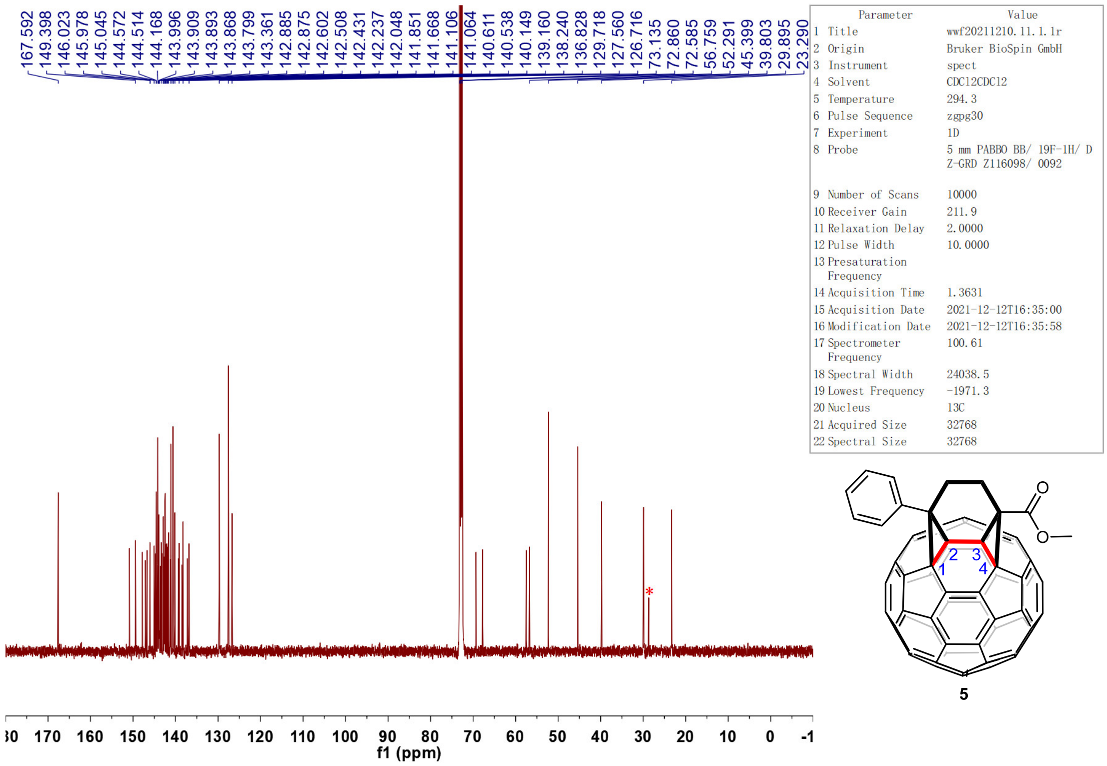

Supplementary Fig. 37. ^{13}C NMR (101 MHz, $C_2D_2Cl_4$) of 5. The symbol * represents the peak of petroleum ether.

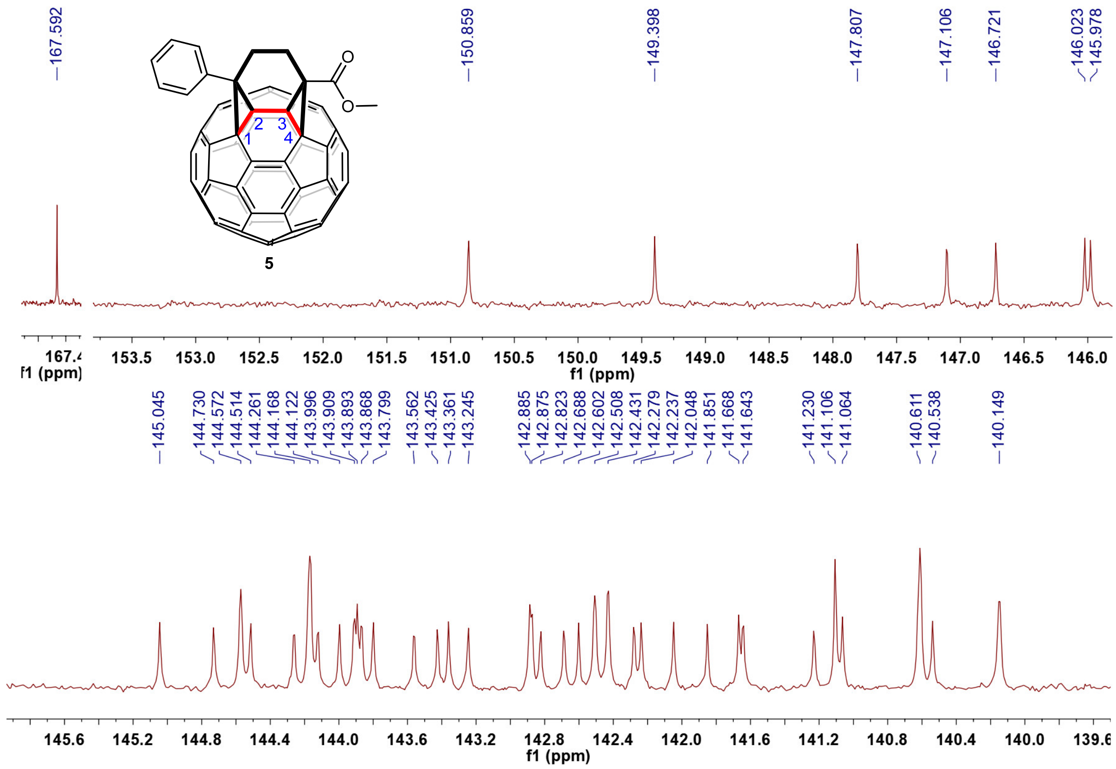

Supplementary Fig. 38. Expanded ^{13}C NMR (101 MHz, $C_2D_2Cl_4$) of 5.

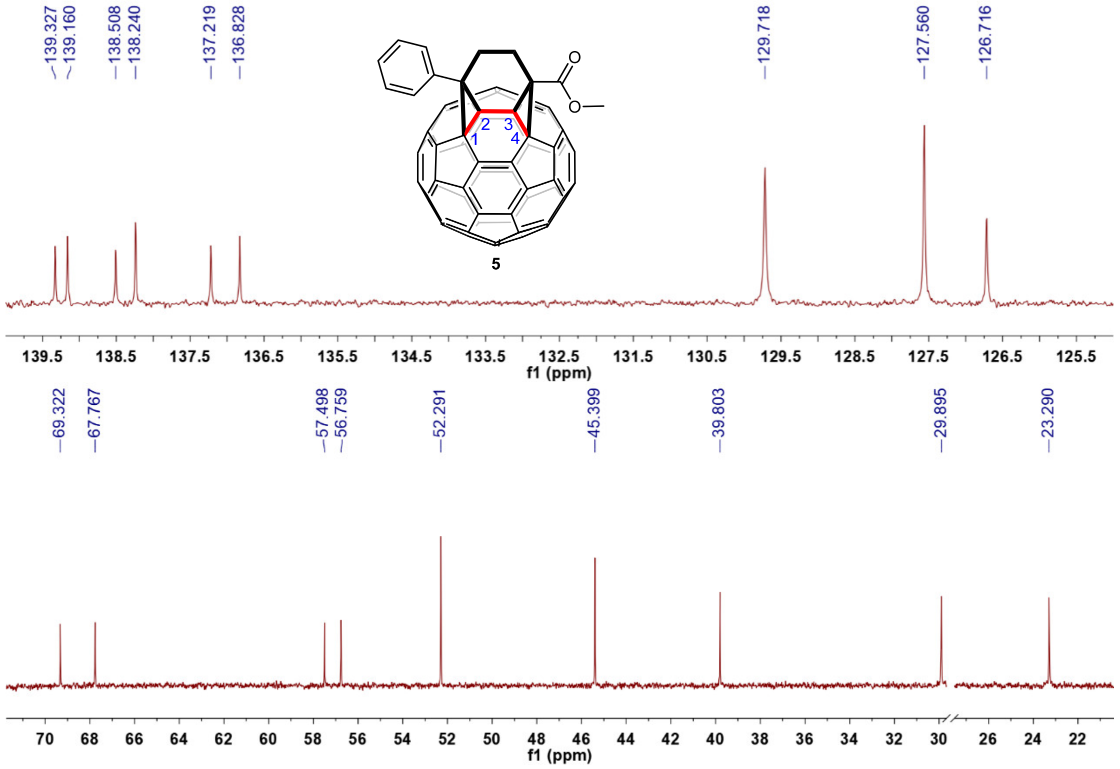

Supplementary Fig. 39. Expanded ^{13}C NMR (101 MHz, $C_2D_2Cl_4$) of 5.

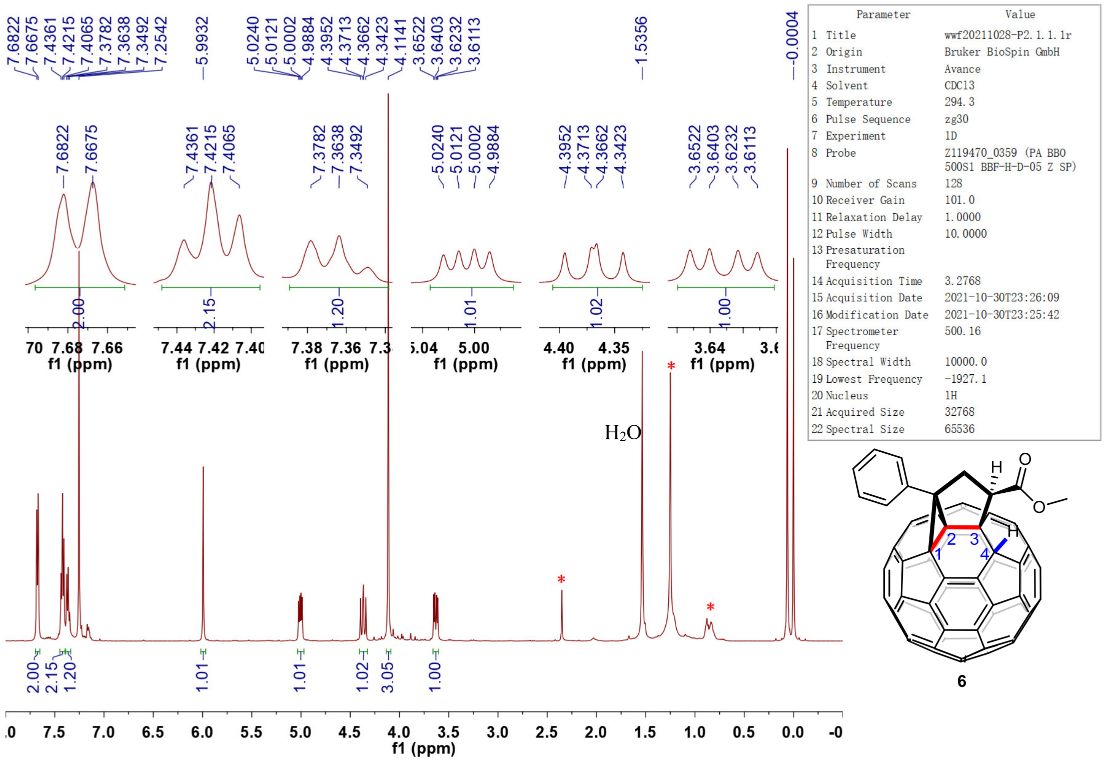

Supplementary Fig. 40. ^{1}H NMR (500 MHz, 1:1 $CS_2/CDCl_3$) of 6. The symbol * represents the peak of methanol and petroleum ether.

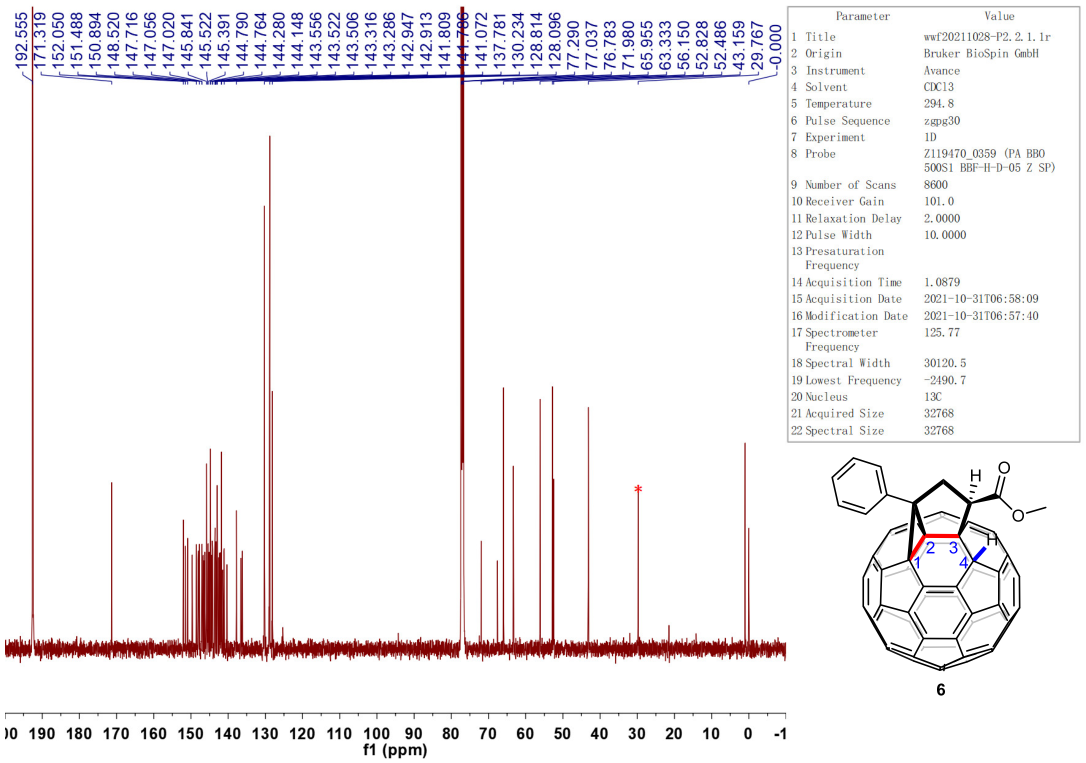

Supplementary Fig. 41. ^{13}C NMR (126 MHz, 1:1 $CS_2/CDCl_3$) of 6. The symbol * represents the peak of petroleum ether.

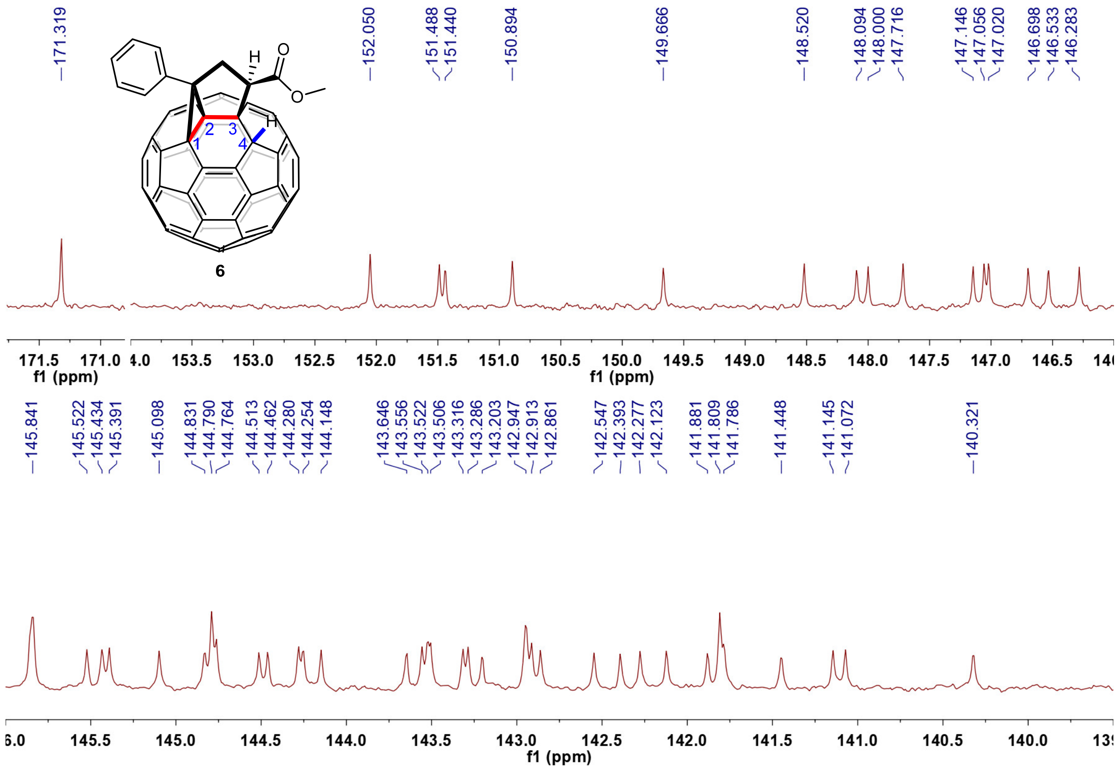

Supplementary Fig. 42. Expanded ^{13}C NMR (126 MHz, 1:1 $CS_2/CDCl_3$) of 6.

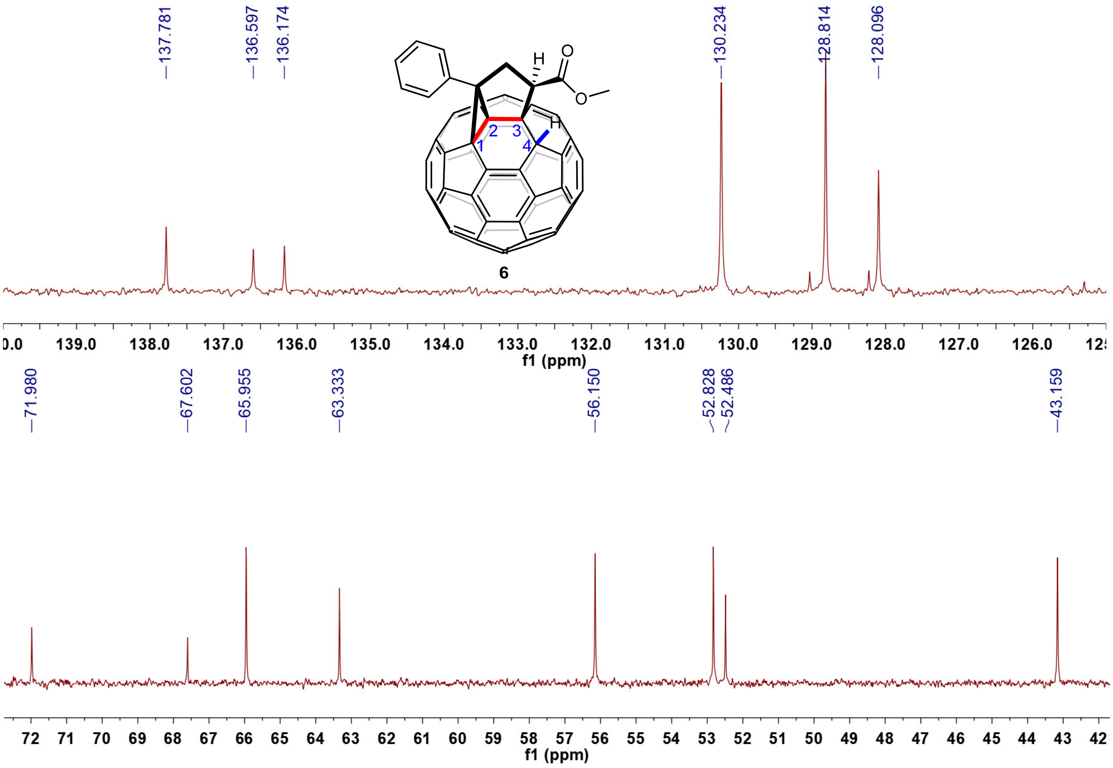

Supplementary Fig. 43. Expanded ^{13}C NMR (126 MHz, 1:1 $CS_2/CDCl_3$) of 6.

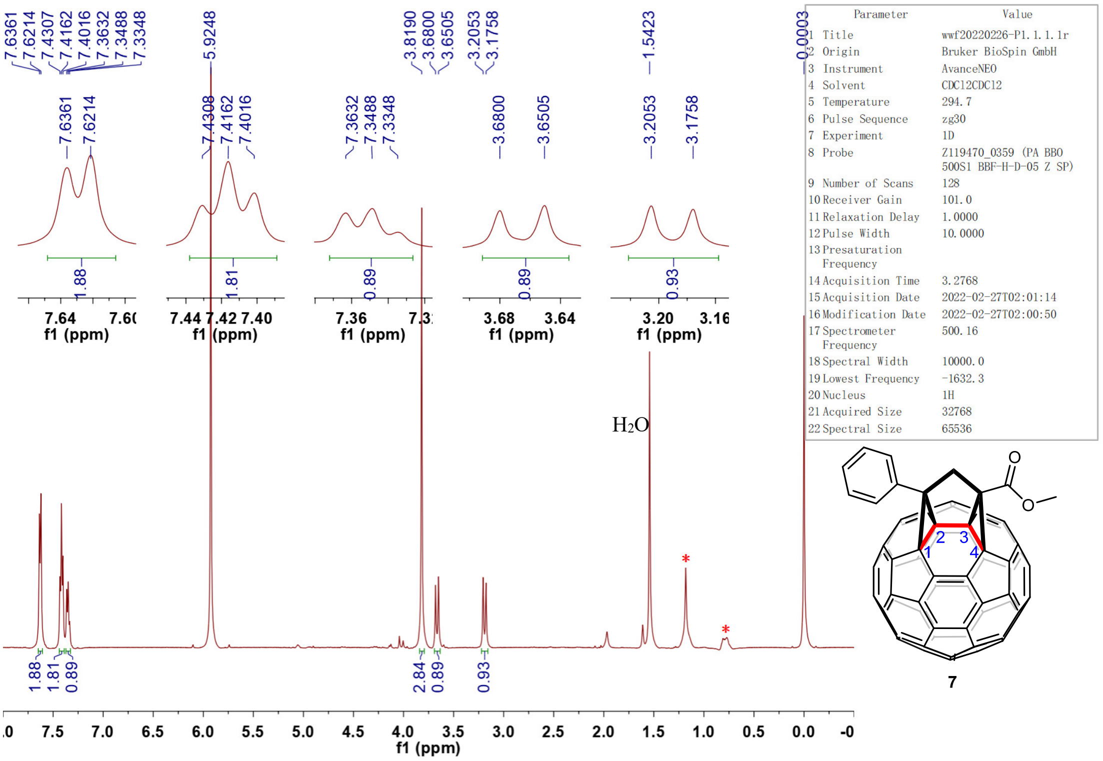

Supplementary Fig. 44. ^{1}H NMR (500 MHz, $C_2D_2Cl_4$) of 7. The symbol * represents the peak of petroleum ether.

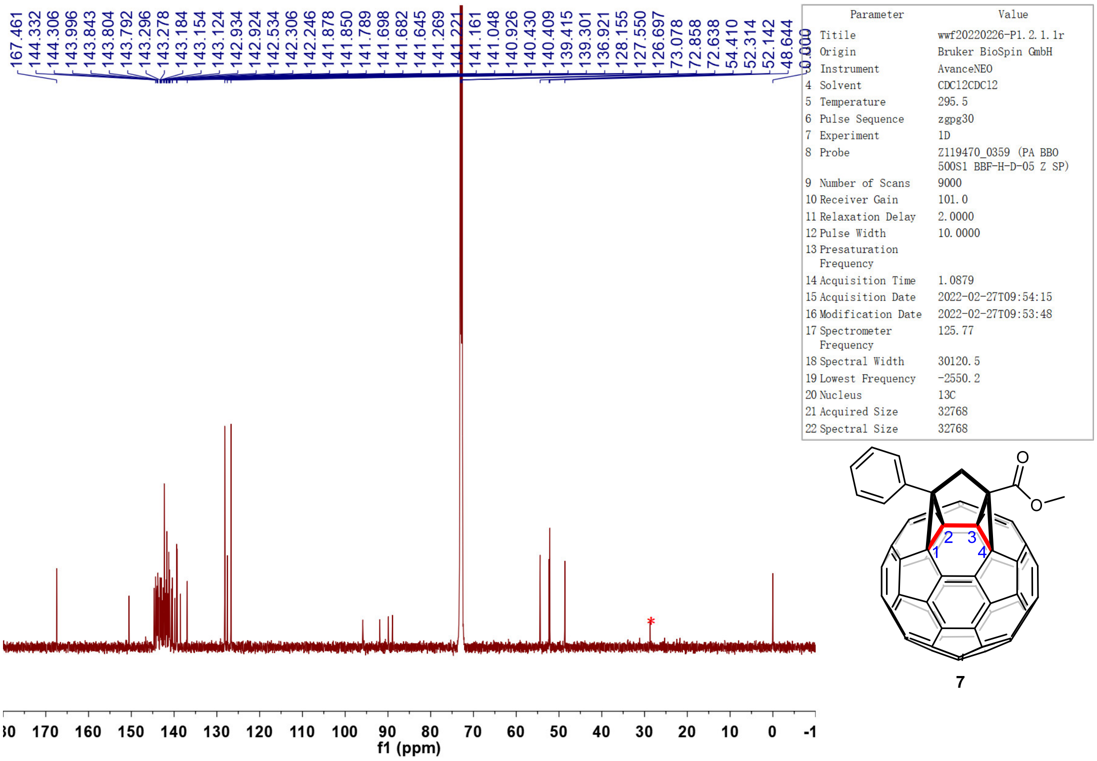

Supplementary Fig. 45. ^{13}C NMR (126 MHz, $C_2D_2Cl_4$) of 7. The symbol * represents the peak of petroleum ether.

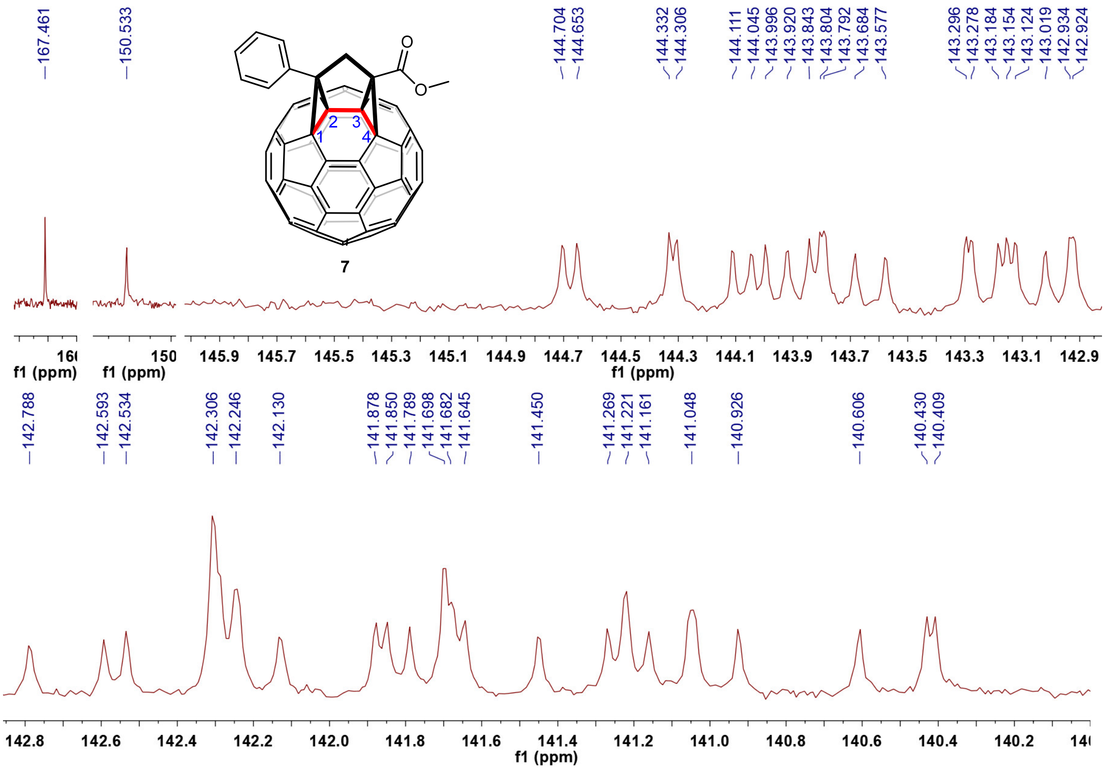

Supplementary Fig. 46. Expanded ^{13}C NMR (126 MHz, $C_2D_2Cl_4$) of 7.

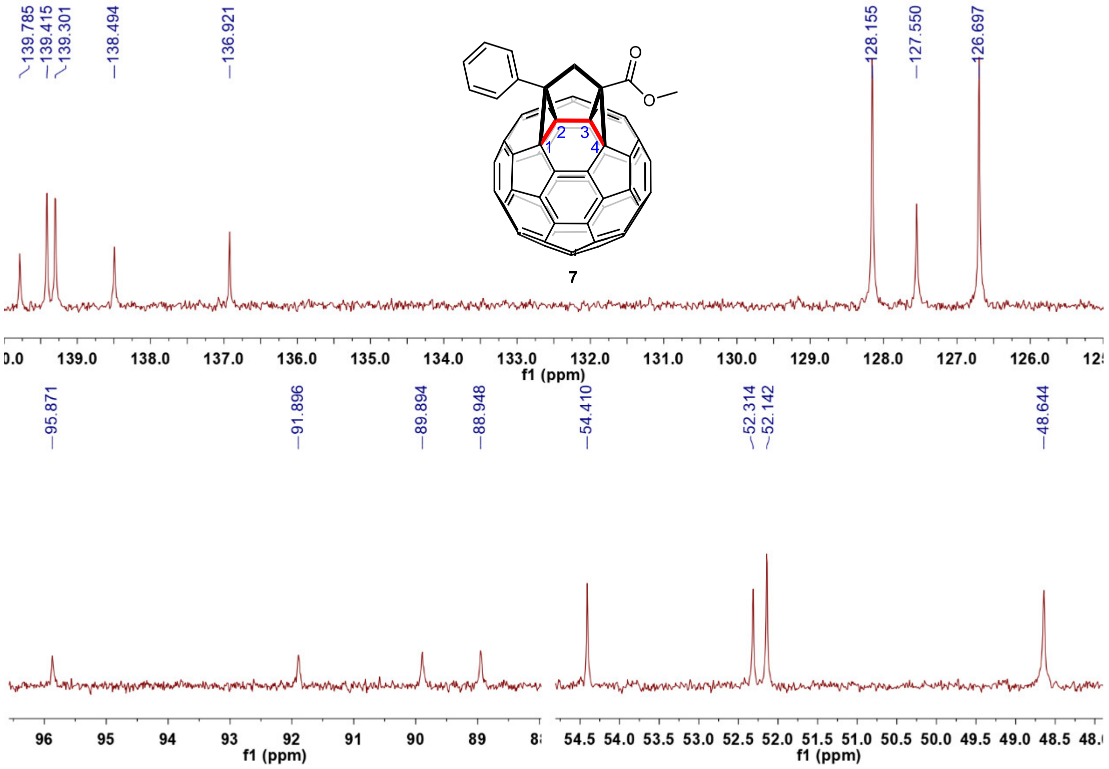

Supplementary Fig. 47. Expanded ^{13}C NMR (126 MHz, $C_2D_2Cl_4$) of 7.

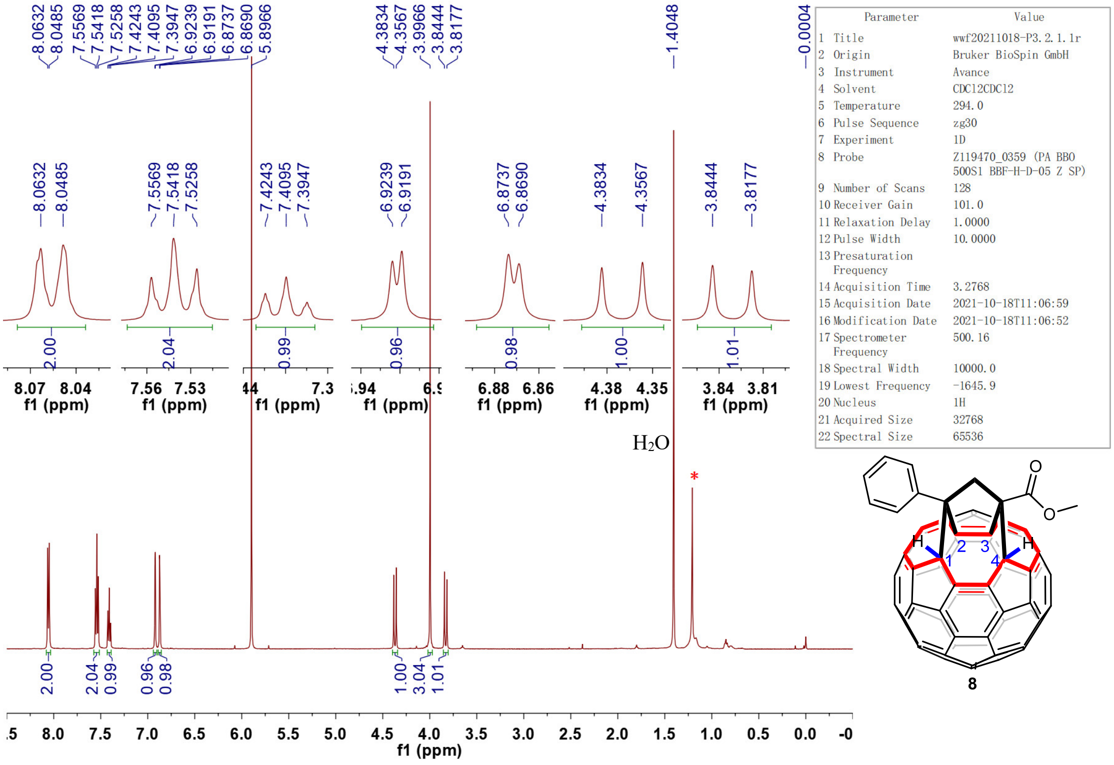

Supplementary Fig. 48. ^{1}H NMR (500 MHz, 1:1 CS_2/$C_2D_2Cl_4$) of 8. The symbol * represents the peak of petroleum ether.

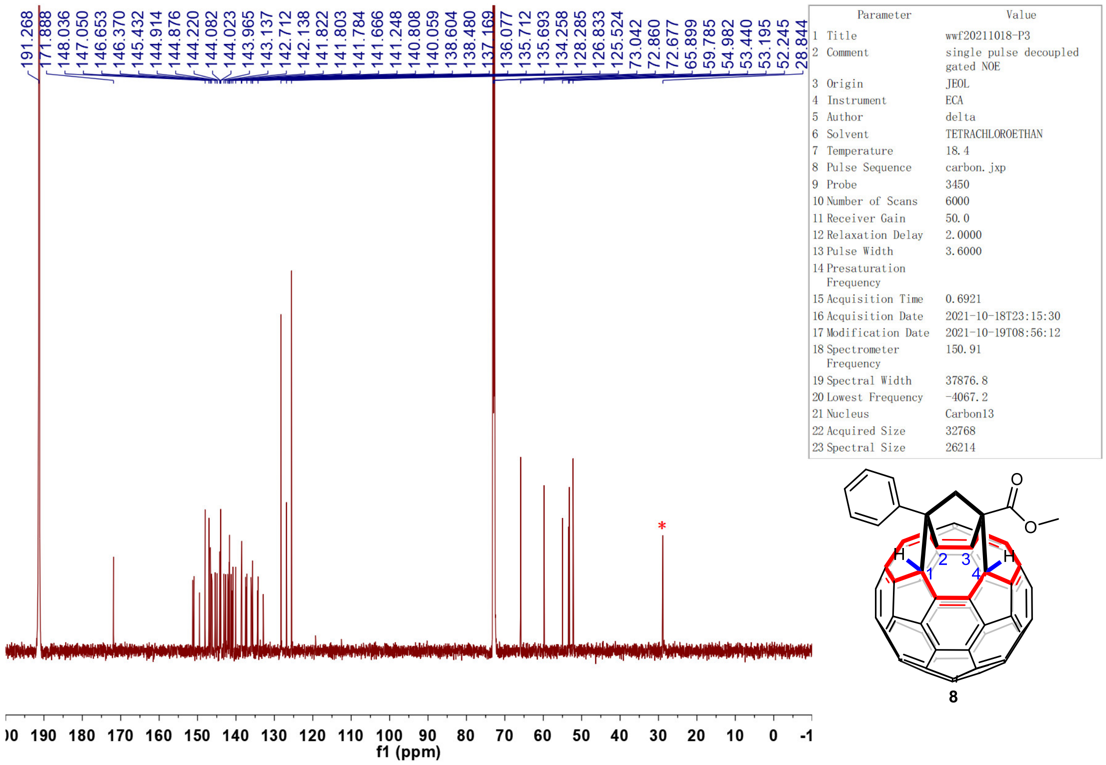

Parameter		Value
1	Title	wwf20211018-P3
2	Comment	single pulse decoupled gated NOE
3	Origin	JEOL
4	Instrument	ECA
5	Author	delta
6	Solvent	TETRACHLOROETHAN
7	Temperature	18.4
8	Pulse Sequence	carbon.jxp
9	Probe	3450
10	Number of Scans	6000
11	Receiver Gain	50.0
12	Relaxation Delay	2.0000
13	Pulse Width	3.6000
14	Presaturation Frequency	
15	Acquisition Time	0.6921
16	Acquisition Date	2021-10-18T23:15:30
17	Modification Date	2021-10-19T08:56:12
18	Spectrometer Frequency	150.91
19	Spectral Width	37876.8
20	Lowest Frequency	-4067.2
21	Nucleus	Carbon13
22	Acquired Size	32768
23	Spectral Size	26214

Supplementary Fig. 49. ^{13}C NMR (151 MHz, 1:1 $CS_2/C_2D_2Cl_4$) of 8. The symbol * represents the peak of petroleum ether.

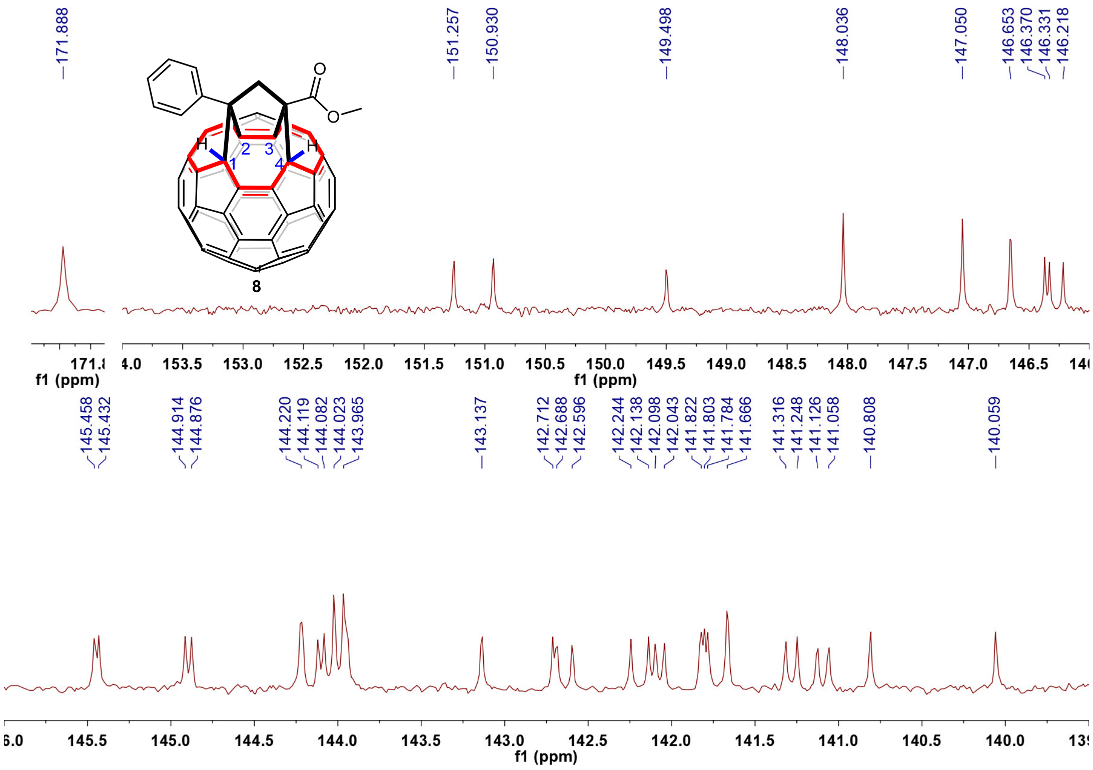

Supplementary Fig. 50. Expanded ^{13}C NMR (151 MHz, 1:1 $CS_2/C_2D_2Cl_4$) of 8.

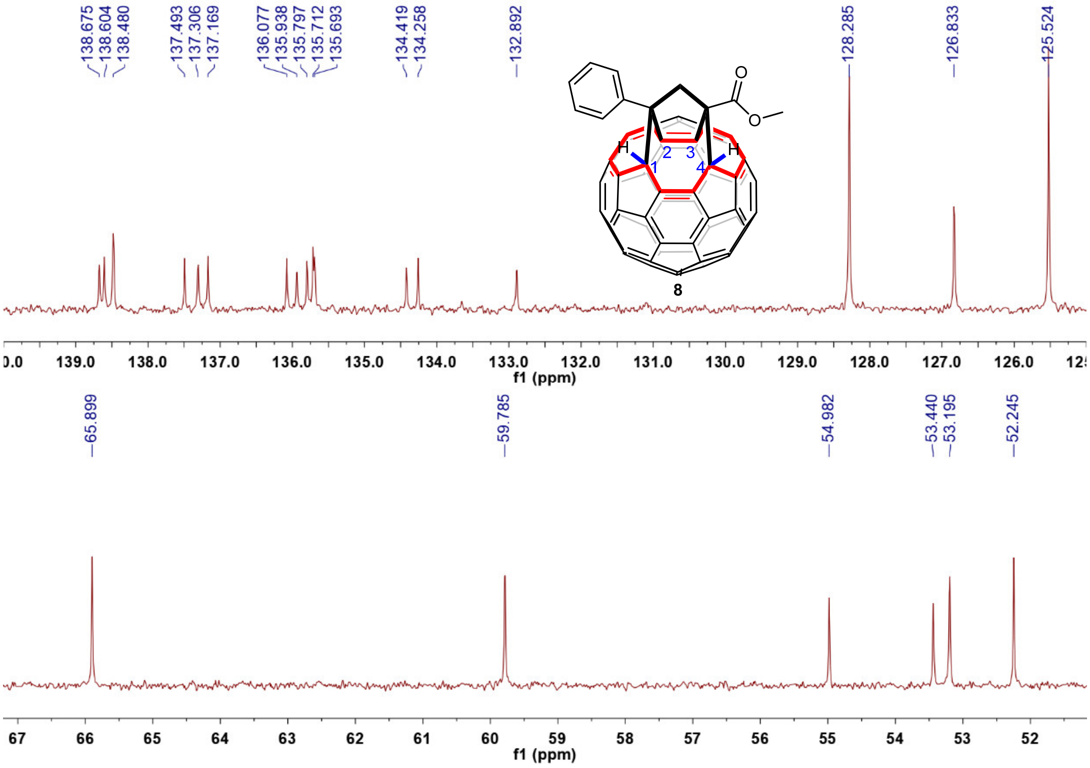

Supplementary Fig. 51. Expanded ^{13}C NMR (151 MHz, 1:1 CS_2/$C_2D_2Cl_4$) of 8.

7. NMR Spectra of the Reaction Mixtures under Condition Screening

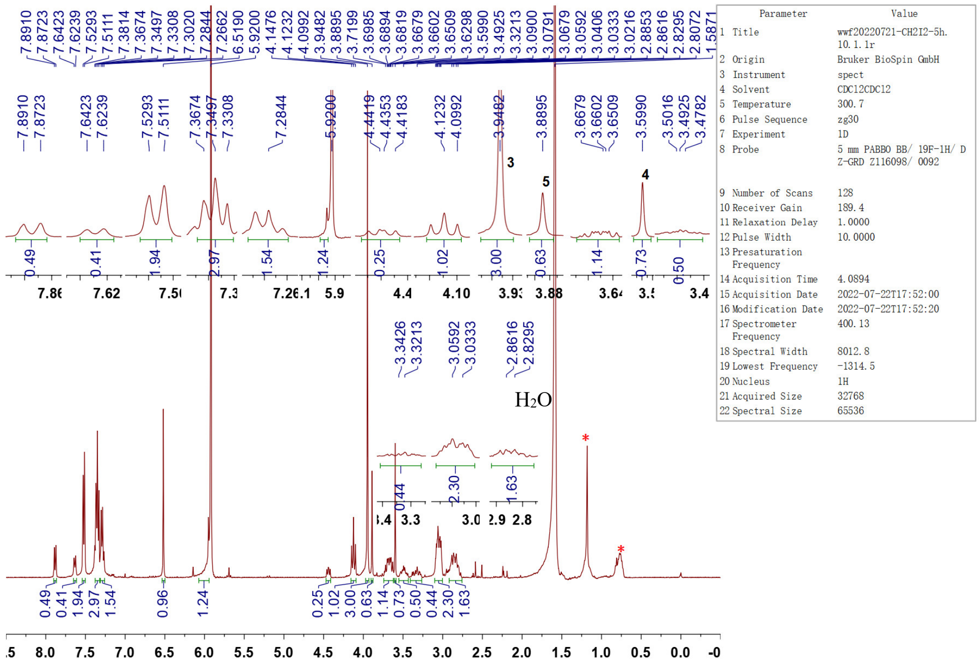

Supplementary Fig. 52. ^{1}H NMR (400 MHz, $C_2D_2Cl_4$) of the mixture containing 3, 4 and 5 from the generation of 1^{2-} followed by treatment with CH_2I_2 for 5 h. The ratio of **3**:**4**:**5** calculated from the integrals based on the methoxy group was 68.8:16.7:14.5. The symbol * represents the peak of petroleum ether.

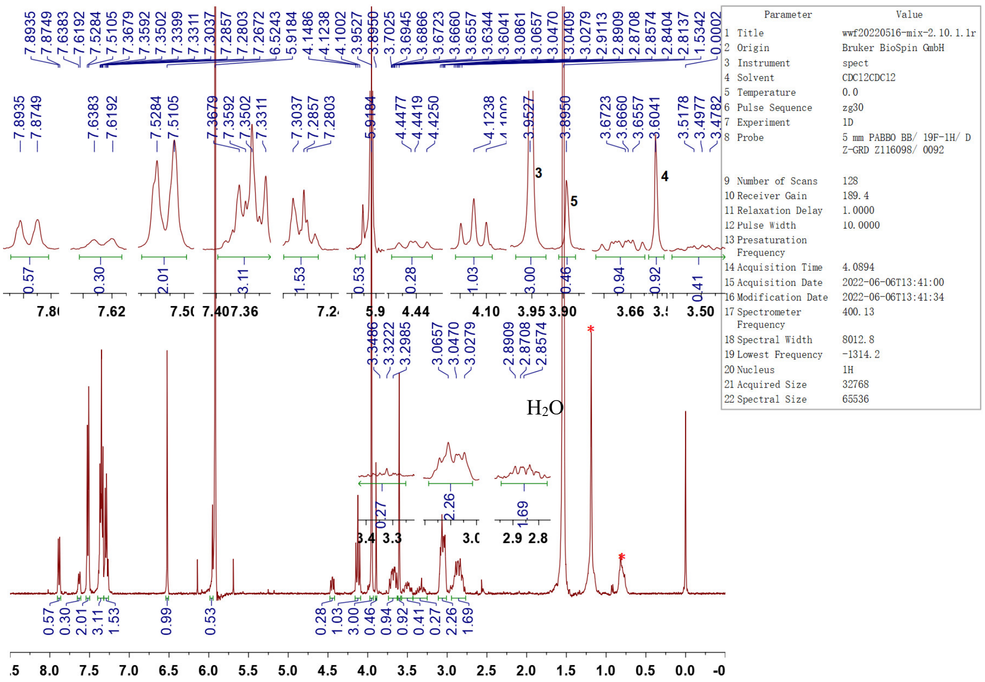

Supplementary Fig. 53. ^{1}H NMR (400 MHz, $C_2D_2Cl_4$) of the mixture containing 3, 4 and 5 from the generation of 1^{2-}, subsequent stirring for 5 h and then treatment with TFA for 10 min. The ratio of **3**, **4** and **5** calculated from the integrals based on the methoxy group was 68.5:21.0:10.5. The symbol * represents the peak of petroleum ether.

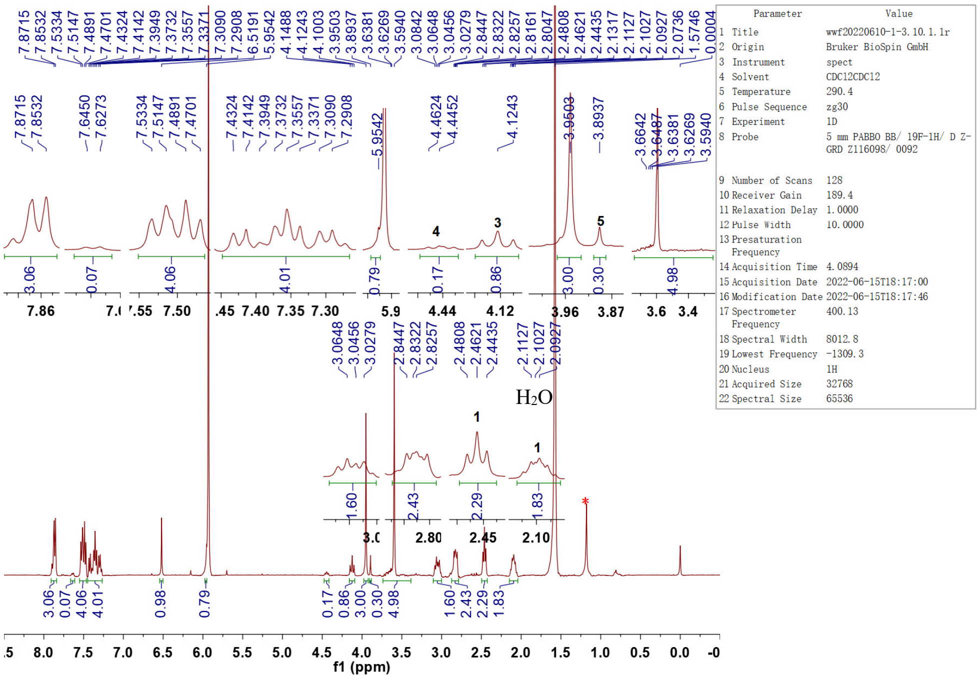

Supplementary Fig. 54. ^{1}H NMR (400 MHz, $C_2D_2Cl_4$) of the mixture containing 3, 4, 5 and recovered 1 from the generation of 1^{2-} followed by immediate treatment with TFA for 10 min. The ratio of **3**:**4**:**5**:**1** calculated from the integrals based on the methoxy group was 39.8:7.9:4.6:47.7. The symbol * represents the peak of petroleum ether.

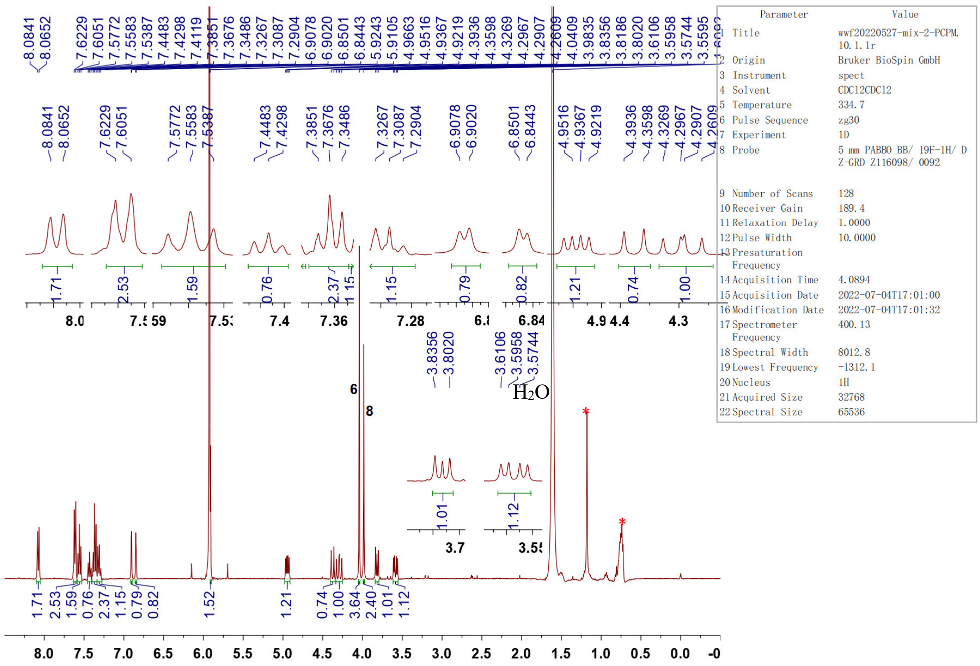

Supplementary Fig. 55. ^{1}H NMR (400 MHz, $C_2D_2Cl_4$) of a mixture containing 6 and 8 from the generation of 2^{2-}, subsequent stirring for 3 h and then treatment with TFA for 10 min. The ratio of **6**/**8** based on the integrals of the methoxy group was 60.3:39.7. The symbol * represents the peak of petroleum ether.

8. NMR Spectrum of the Reaction Mixture under Control Experiment

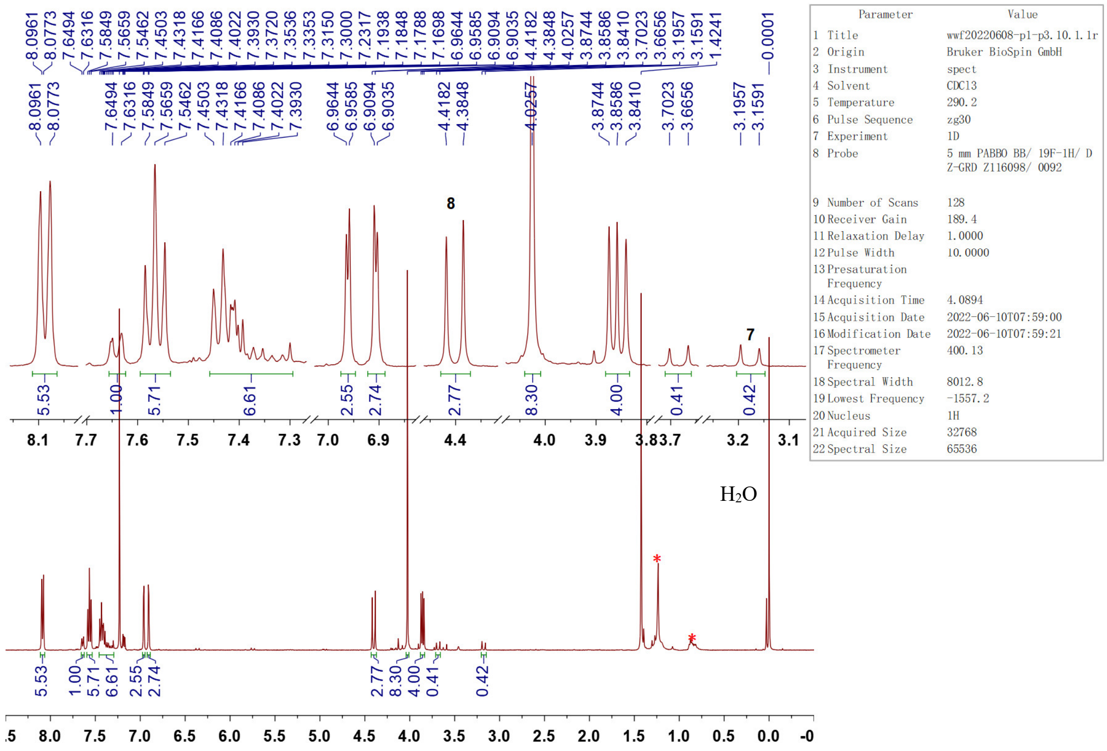

Supplementary Fig. 56. ^{1}H NMR (400 MHz, 1:1 $CS_2/CDCl_3$) of 7 and 8. The ratio of **7**/**8** based on the integrals of the methoxy group was 13.2: 86.8. The symbol * represents the peak of petroleum ether.

9. Single-Crystal X-Ray Crystallography of 3, 4, 7 and 8

Black block crystals of **3** were obtained by slow evaporation of a saturated solution in CS_2 at about 25 °C. Single-crystal X-ray diffraction data were obtained at 293(2) K and collected on a diffractometer (SuperNova, Agilent Technologies) equipped with a CCD area detector using graphite-monochromated CuKα radiation (λ = 1.54184 Å) in the scan range 6.914° < 2θ < 139.83°. Using Olex2, the structure was solved with the ShelXT[4] structure solution program using Intrinsic Phasing and refined with the ShelXL[5] refinement package using Least Squares Minimisation. Crystallographic data have been deposited in the Cambridge Crystallographic Data Centre as deposition number CCDC 2235544.

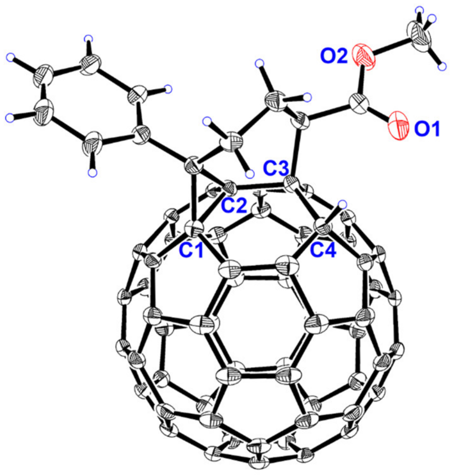

Supplementary Fig. 57. Oak Ridge thermal ellipsoid plot (ORTEP) drawing of compound 3 (CCDC 2235544) with thermal ellipsoids at the 20% probability level. The CS_2 molecule is omitted for clarity.

Datablock: 1

Bond precision:	C-C = 0.0070 A		Wavelength=1.54184
Cell:	a=14.4274(6)	b=9.9907(4)	c=27.5502(11)
	alpha=90	beta=90	gamma=90
Temperature:	293 K		

	Calculated	Reported
Volume	3971.1(3)	3971.1(3)
Space group	P n m a	P n m a
Hall group	-P 2ac 2n	-P 2ac 2n
Moiety formula	2(C36 H7 O), C S2	C S2, 0.5(C144 H28 O4)
Sum formula	C73 H14 O2 S2	C73 H14 O2 S2
Mr	986.96	986.96
Dx,g cm-3	1.651	1.651
Z	4	4
Mu (mm-1)	1.724	1.724
F000	2000.0	2000.0
F000'	2008.11	
h,k,lmax	17,12,33	17,11,33
Nref	3994	3920
Tmin,Tmax	0.830,0.902	0.830,0.902
Tmin'	0.813	

Correction method= # Reported T Limits: Tmin=0.830 Tmax=0.902
AbsCorr = MULTI-SCAN

Data completeness= 0.981 Theta(max)= 69.970

R(reflections)= 0.0804(3018) wR2(reflections)= 0.2435(3920)

S = 1.091 Npar= 684

The following ALERTS were generated. Each ALERT has the format
test-name_ALERT_alert-type_alert-level.
Click on the hyperlinks for more details of the test.

Alert level A

PLAT088_ALERT_3_A Poor Data / Parameter Ratio .... 5.73 Note

Author Response: This is caused by the fullerene disorder.

Alert level C

PLAT042_ALERT_1_C Calc. and Reported MoietyFormula Strings Differ Please Check
PLAT220_ALERT_2_C NonSolvent Resd 1 C Ueq(max)/Ueq(min) Range 3.2 Ratio
PLAT244_ALERT_4_C Low 'Solvent' Ueq as Compared to Neighbors of C1S Check
PLAT260_ALERT_2_C Large Average Ueq of Residue Including S1S 0.142 Check
PLAT340_ALERT_3_C Low Bond Precision on C-C Bonds .... 0.007 Ang.
PLAT906_ALERT_3_C Large K Value in the Analysis of Variance ...... 11.178 Check
PLAT906_ALERT_3_C Large K Value in the Analysis of Variance ...... 2.443 Check
PLAT911_ALERT_3_C Missing FCF Refl Between Thmin & STh/L= 0.600 10 Report
PLAT921_ALERT_1_C R1 in the CIF and FCF Differ by .... -0.0013 Check
PLAT922_ALERT_1_C wR2 in the CIF and FCF Differ by .... -0.0024 Check
PLAT923_ALERT_1_C S Values in the CIF and FCF Differ by .... -0.012 Check

Black block crystals of **4** were obtained by slow evaporation of decapyrrylcorannulene (abbreviated as DPC) in toluene at about 25 °C.[6] Single-crystal X-ray diffraction data were obtained at 106(4) K and collected on a diffractometer (SuperNova, Agilent Technologies) equipped with a CCD area detector using graphite-monochromated CuKα radiation (λ = 1.54184 Å) in the scan range 6.808° < 2θ < 158.876°. Using Olex2, the structure was solved with the ShelXT[4] structure solution program using Intrinsic Phasing and refined with the ShelXL[5] refinement package using Least Squares Minimisation. Crystallographic data have been deposited in the Cambridge Crystallographic Data Centre as deposition number CCDC 2263924.

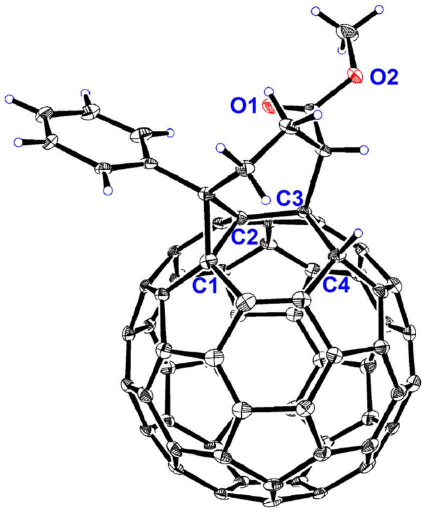

Supplementary Fig. 58. Oak Ridge thermal ellipsoid plot (ORTEP) drawing of compound 4 (CCDC 2263924) with thermal ellipsoids at the 20% probability level. The toluene and decapyrrylcorannulene molecules are omitted for clarity.

Datablock: wwf_bp2

Bond precision:	C-C = 0.0035 A		Wavelength=1.54184
Cell:	a=18.3791(2)	b=32.0444(5)	c=22.1646(3)
	alpha=90	beta=90.953(1)	gamma=90
Temperature:	106 K		

	Calculated	Reported
Volume	13052.0(3)	13052.0(3)
Space group	P 21/n	P 1 21/n 1
Hall group	-P 2yn	-P 2yn
Moiety formula	C72 H14 O2, 2(C60 H40 N10), C7 H8	C7 H8, C72 H14 O2, 2(C60 H40 N10)
Sum formula	C199 H102 N20 O2	C199 H102 N20 O2
Mr	2805.01	2805.00
Dx,g cm-3	1.428	1.427
Z	4	4
Mu (mm-1)	0.675	0.675
F000	5808.0	5808.0
F000'	5824.20	
h,k,lmax	23,40,28	23,40,27
Nref	28348	27418
Tmin,Tmax	0.968,0.997	0.968,0.997
Tmin'	0.935	

Correction method= # Reported T Limits: Tmin=0.968 Tmax=0.997
AbsCorr = MULTI-SCAN

Data completeness= 0.967 Theta(max)= 79.438

R(reflections)= 0.0586(17073) wR2(reflections)= 0.1580(27418)

S = 1.021 Npar= 2723

The following ALERTS were generated. Each ALERT has the format
test-name_ALERT_alert-type_alert-level.
Click on the hyperlinks for more details of the test.

Alert level C

```
PLAT042_ALERT_1_C Calc. and Reported MoietyFormula Strings  Differ     Please Check
PLAT601_ALERT_2_C Unit Cell Contains Solvent Accessible VOIDS of .        47 Ang**3
PLAT906_ALERT_3_C Large K Value in the Analysis of Variance ......     10.963 Check
PLAT906_ALERT_3_C Large K Value in the Analysis of Variance ......      2.546 Check
PLAT910_ALERT_3_C Missing # of FCF Reflection(s) Below Theta(Min).         6 Note
PLAT911_ALERT_3_C Missing FCF Refl Between Thmin & STh/L=     0.600       14 Report
```

Black block crystals of **7** were obtained by slow evaporation of a saturated solution containing product **7** in CS_2 mixed with a saturated solution of decapyrrylcorannulene (abbreviated as DPC) in toluene at about 0 °C. Single-crystal X-ray diffraction data were obtained at 100.00(10) K and collected on a diffractometer (SuperNova, Agilent Technologies) equipped with a CCD area detector using graphite-monochromated CuKα radiation (λ = 1.54184 Å) in the scan range 7.022° < 2θ < 146.172°. Using Olex2, the structure was solved with the ShelXT[4] structure solution program using Direct Methods and refined with the ShelXL[5] refinement package using Least Squares Minimisation. Crystallographic data have been deposited in the Cambridge Crystallographic Data Centre as deposition number CCDC 2235545.

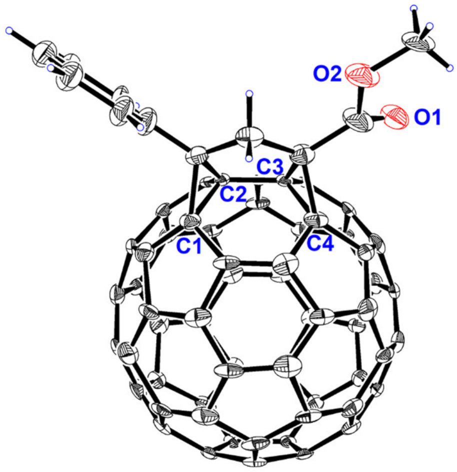

Supplementary Fig. 59. Oak Ridge thermal ellipsoid plot (ORTEP) drawing of compound 7 (CCDC 2235545) with thermal ellipsoids at the 10% probability level. The decapyrrylcorannulene molecule is omitted for clarity.

Datablock: 7

Bond precision:	C-C = 0.0058 A		Wavelength=1.54184
Cell:	a=15.3096(5)	b=16.3131(8)	c=18.9640(7)
	alpha=98.478(4)	beta=94.317(3)	gamma=105.922(3)
Temperature:	100 K		

	Calculated	Reported
Volume	4471.3(3)	4471.3(3)
Space group	P -1	P -1
Hall group	-P 1	-P 1
Moiety formula	C71 H10 O2, C60 H40 N10 [+ solvent]	1(C71 H10 O2), C60 H40 N10
Sum formula	C131 H50 N10 O2 [+ solvent]	C131 H50 N10 O2
Mr	1795.81	1795.81
Dx,g cm-3	1.334	1.334
Z	2	2
Mu (mm-1)	0.633	0.633
F000	1844.0	1844.0
F000'	1849.24	
h,k,lmax	18,20,23	18,20,23
Nref	17907	17377
Tmin,Tmax	0.892,0.945	0.892,0.945
Tmin'	0.892	

Correction method= # Reported T Limits: Tmin=0.892 Tmax=0.945
AbsCorr = MULTI-SCAN

Data completeness= 0.970 Theta(max)= 73.086

R(reflections)= 0.0804(10056) wR2(reflections)= 0.2365(17377)

S = 1.004 Npar= 1948

The following ALERTS were generated. Each ALERT has the format
test-name_ALERT_alert-type_alert-level.
Click on the hyperlinks for more details of the test.

Alert level C

```
PLAT088_ALERT_3_C Poor Data / Parameter Ratio ....................       8.92 Note
PLAT220_ALERT_2_C NonSolvent   Resd 1  C   Ueq(max)/Ueq(min) Range        3.4 Ratio
PLAT234_ALERT_4_C Large Hirshfeld Difference O2A      --C71A     .       0.18 Ang.
PLAT234_ALERT_4_C Large Hirshfeld Difference C67A     --C68A     .       0.16 Ang.
PLAT340_ALERT_3_C Low Bond Precision on  C-C Bonds ...............    0.00578 Ang.
PLAT906_ALERT_3_C Large K Value in the Analysis of Variance ......      5.242 Check
PLAT911_ALERT_3_C Missing FCF Refl Between Thmin & STh/L=     0.600        19 Report
PLAT977_ALERT_2_C Check Negative Difference Density on H65B      .      -0.37 eA-3
```

Black block crystals of **8** were obtained by slow diffusion of hexane into a saturated solution in CS_2 at about 25 °C. Single-crystal X-ray diffraction data were obtained at 100.15 K and collected on a diffractometer (SuperNova, Agilent Technologies) equipped with a CCD area detector using graphite-monochromated CuKα radiation (λ = 1.54184 Å) in the scan range 8.536° < 2θ < 133.18°. Using Olex2, the structure was solved with the ShelXT[4] structure solution program using Intrinsic Phasing and refined with the ShelXL[5] refinement package using Least Squares Minimisation. Crystallographic data have been deposited in the Cambridge Crystallographic Data Centre as deposition number CCDC 2235547.

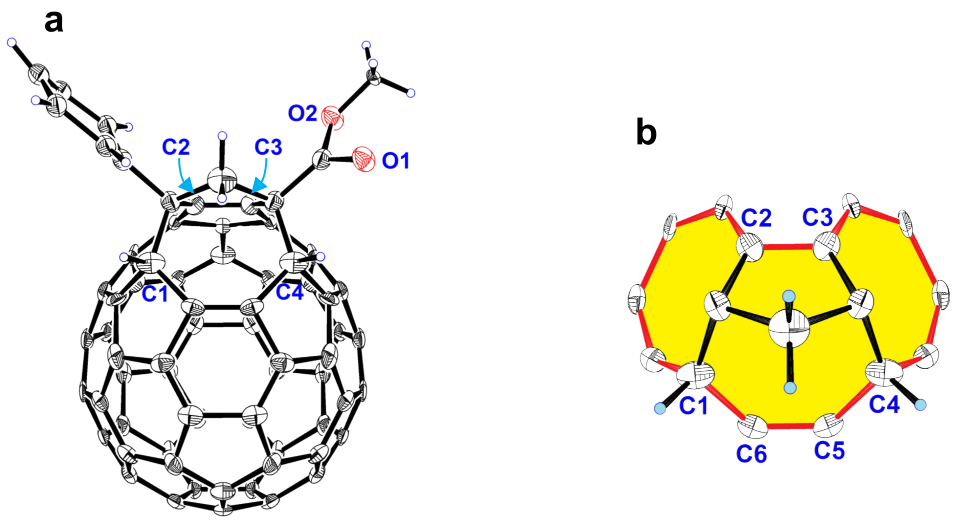

Supplementary Fig. 60. Oak Ridge thermal ellipsoid plot (ORTEP) drawing of compound 8 (CCDC 2235547) and view of 8 in the vicinity of the addend. a ORTEP drawing of compound **8** with thermal ellipsoids at the 20% probability level. **b** View of **8** in the vicinity of the addend.

Datablock: wwf-0929_auto

Bond precision: C-C = 0.0160 A Wavelength=1.54184

Cell:	a=10.360(3)	b=19.356(4)	c=17.377(4)
	alpha=90	beta=90	gamma=90
Temperature:	100 K		

	Calculated	Reported
Volume	3484.6(15)	3484.5(15)
Space group	P b c m	P b c m
Hall group	-P 2c 2b	-P 2c 2b
Moiety formula	C71 H12 O2	C71 H12 O2
Sum formula	C71 H12 O2	C71 H12 O2
Mr	896.81	896.81
Dx,g cm-3	1.709	1.709
Z	4	4
Mu (mm-1)	0.803	0.803
F000	1816.0	1816.0
F000'	1821.30	
h,k,lmax	12,23,20	12,23,20
Nref	3192	3177
Tmin,Tmax	0.926,0.961	
Tmin'	0.923	

Correction method= Not given

Data completeness= 0.995 Theta(max)= 66.590

R(reflections)= 0.1181(887) wR2(reflections)= 0.3467(3177)

S = 0.947 Npar= 347

The following ALERTS were generated. Each ALERT has the format
test-name_ALERT_alert-type_alert-level.
Click on the hyperlinks for more details of the test.

Alert level A

PLAT026_ALERT_3_A Ratio Observed / Unique Reflections (too) Low .. 28% Check

Author Response: This is due to the weak diffraction density at the high angle region.

Alert level B

RINTA01_ALERT_3_B The value of Rint is greater than 0.18
Rint given 0.242

Author Response: This is due to the weak diffraction density at the high angle region.

PLAT020_ALERT_3_B The Value of Rint is Greater Than 0.12 .... 0.242 Report

Author Response: This is due to the weak diffraction density at the high angle region.

PLAT340_ALERT_3_B Low Bond Precision on C-C Bonds .... 0.01602 Ang.

Author Response: The low C-C bond precision is cased by the weak diffraction density at the high angle region.

10. Calculated Partial Natural Bond Orbital Charge Distributions of IV-1 and IV-2

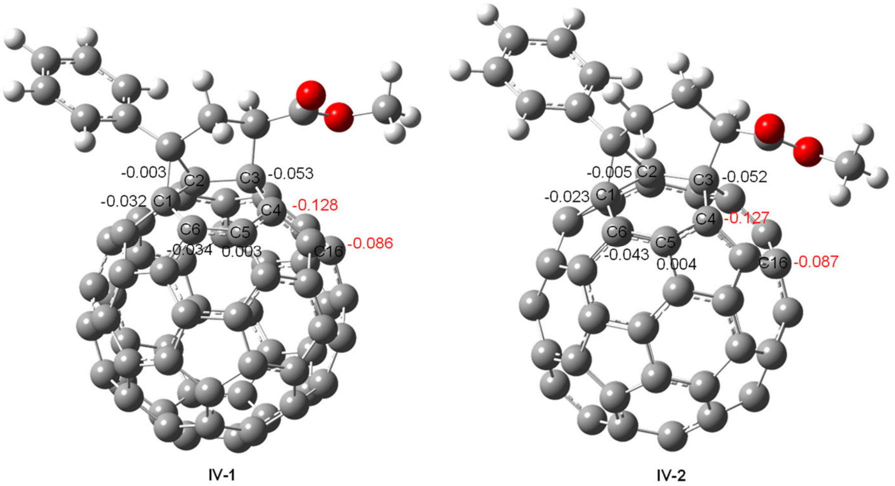

Supplementary Fig. 61. Calculated partial natural bond orbital (NBO) charge distributions of IV (IV-1 and IV-2) at the B3LYP/6-31G(d) level.

11. Calculated Energies for Optimised 3, 4, 3'', 6, 6' and 6''

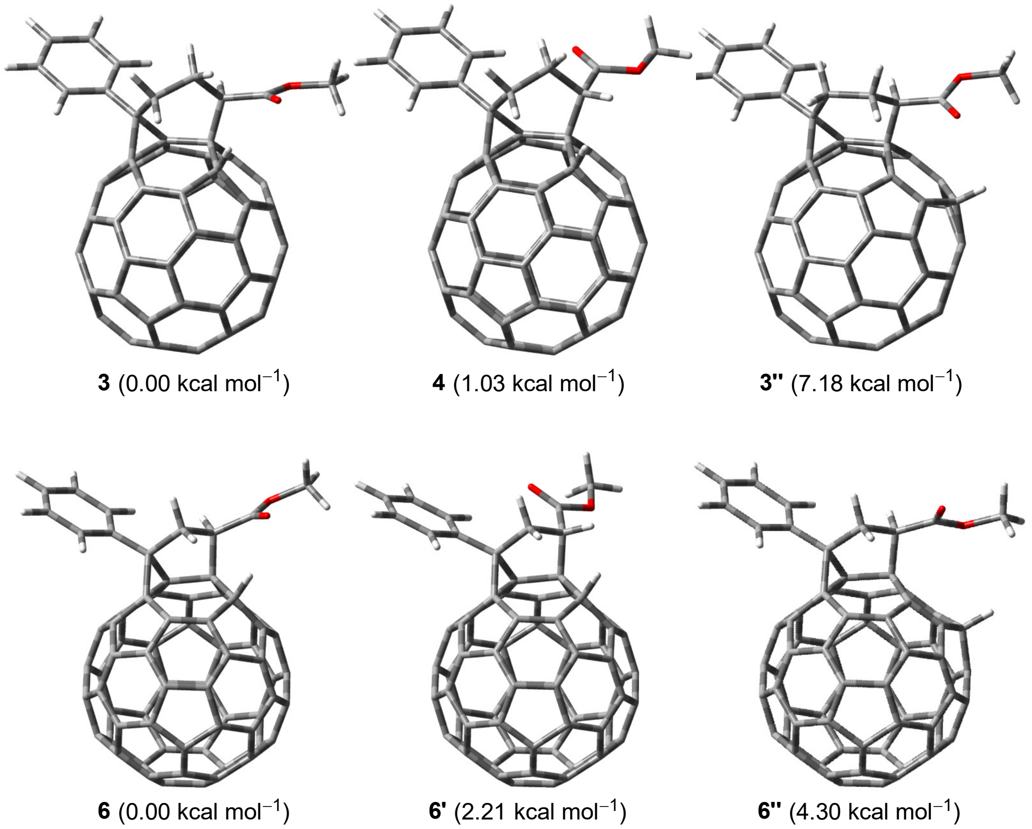

Supplementary Fig. 62. Relative energies for optimised 3, 4, 3'', 6, 6' and 6'' at the B3LYP/6-31G(d) level.

The Boltzmann distribution is a probability distribution that gives the probability of being in a certain quantum state at a certain energy and temperature.[7-9] It is defined as follows:

$$p_i = \frac{1}{Q} e^{-\varepsilon_i/kT} = \frac{e^{-\varepsilon_i/kT}}{\sum_{j=1}^{M} e^{-\varepsilon_j/kT}} \quad \text{eq (S1)}$$

p_i is the probability of state i, ε_i is the energy of state i, k is the Boltzmann constant, T is the temperature of the system, and M is the total number of quantum states that the system can reach. Here, for the sake of conciseness and beauty, the parentheses around kT are omitted. The normalised denominator Q (written by some authors as Z) is the sum for all quantum states in the system; this part is also known as the canonical partition function. The sum of the probabilities of all quantum states is 1.

The Boltzmann distribution indicates that low-energy quantum states always have a higher probability of being occupied by particles than high-energy quantum states. It also allows us to quantitatively compare the relationship between the two quantum state probability distributions. Probability ratio of state i and j:

$$\frac{p_i}{p_j} = e^{(\varepsilon_j - \varepsilon_i)/kT} \quad \text{eq (S2)}$$

p_i is the probability of quantum state i, p_j is the probability of quantum state j, and ε_i and ε_j are the energies of states i and j, respectively. In general, the yield ratios of the stereoisomers are calculated according to eq (S2) at the B3LYP/6-31G(d) level.

$$\frac{\mathbf{3}}{\mathbf{4}} = e^{(1.03*4.184*1000\ \text{J/mol}\ /\ (1.38*10^{-23}\ \text{J/K}\ *298\ \text{K}*6.02*10^{23}\ /\text{mol}))} = 5.7$$

$$\frac{\mathbf{6}}{\mathbf{6'}} = e^{(2.21*4.184*1000\ \text{J/mol}\ /\ (1.38*10^{-23}\ \text{J/K}\ *298\ \text{K}*6.02*10^{23}\ /\text{mol}))} = 41.9$$

The yield ratio of **3**/**4** was calculated to be 5.7:1, which is consistent with the experimental result. Meanwhile, the yield ratio of **6**/**6'** was calculated to be 41.9:1. Compound **6'** could not be identified probably due to its extremely small amount under our experimental conditions.

12. Calculated Partial Natural Bond Orbital Charge Distributions of VII

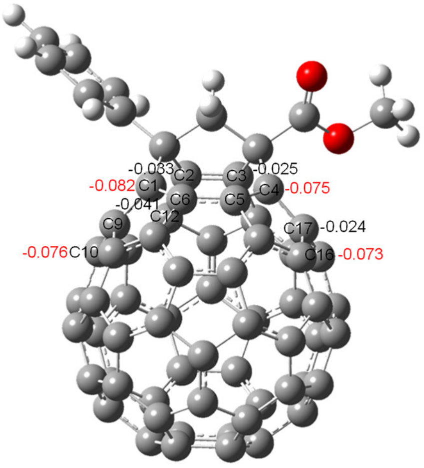

Supplementary Fig. 63. Calculated partial natural bond orbital (NBO) charge distributions of VII at the B3LYP/6-31G(d) level.

13. Calculated Energies for Optimised VIII−XI

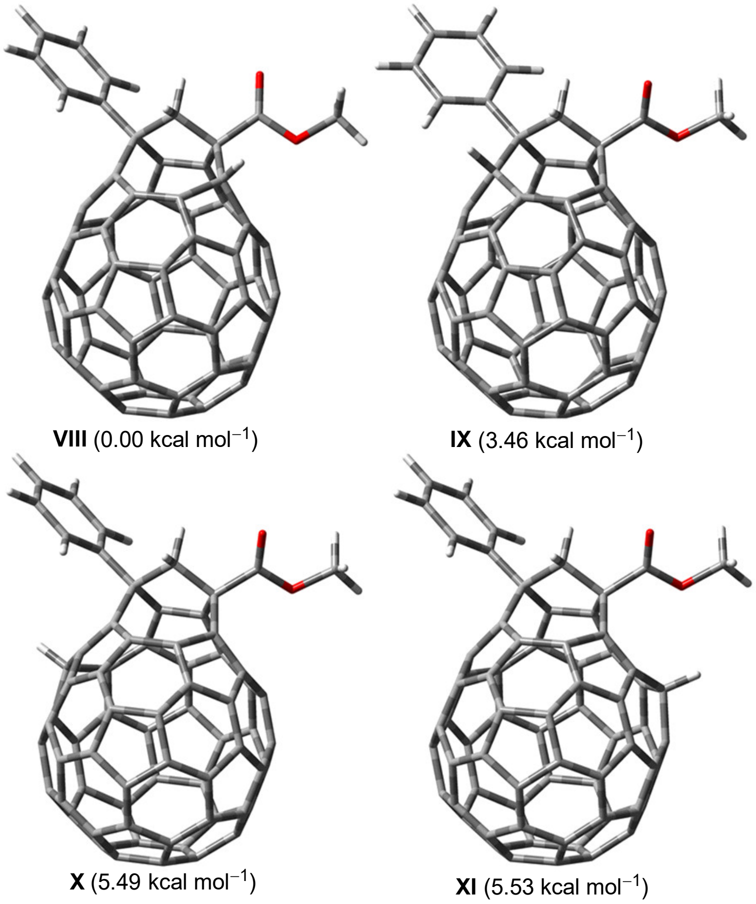

Supplementary Fig. 64. Relative energies for optimised VIII−XI at the B3LYP/6-31G(d) level.

14. Calculated Partial Natural Bond Orbital Charge Distributions of VIII

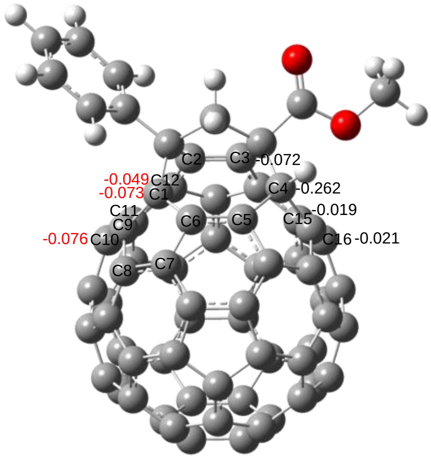

Supplementary Fig. 65. Calculated partial natural bond orbital (NBO) charge distributions of VIII at the B3LYP/6-31G(d) level.

15. Calculated Energies for Optimised 8, 8' and 8''

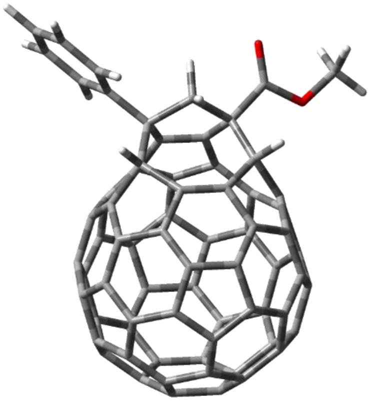

8 (0.00 kcal mol^{-1})

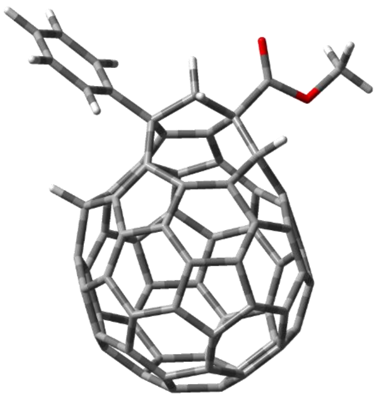

8' (7.26 kcal mol^{-1})

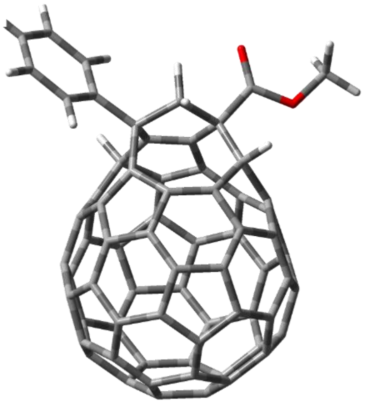

8'' (16.38 kcal mol^{-1})

Supplementary Fig. 66. Relative energies for optimised 8, 8' and 8'' at the B3LYP/6-31G(d) level.

16. Characteristic Potential Values and Energy Levels of 1–8

Supplementary Table 1. Characteristic potential values and energy levels of 1–8.

Compd	E_{red}^{onset} (V)[a]	λ_{onset}[b] (nm)	$E_{g,opt}$[c] (eV)	E_{LUMO}[d] (eV)	E_{HOMO}[e] (eV)
**1**	–1.080	726	1.708	–3.720	–5.428
**2**	–1.075	725	1.710	–3.725	–5.435
**3**	–1.097	731	1.696	–3.703	–5.399
**4**	–1.101	733	1.692	–3.699	–5.391
**5**	–1.124	720	1.722	–3.676	–5.398
**6**	–1.026	724	1.713	–3.774	–5.487
**7**	–0.842	728	1.703	–3.958	–5.661
**8**	–0.822	723	1.715	–3.978	–5.693

[a]Versus ferrocene/ferrocenium. Experimental conditions: 1.0 mM compound and 0.1 M tetra-*n*-butylammonium perchlorate in anhydrous 1,2-$C_6H_4Cl_2$; reference electrode: saturated calomel electrode; working electrode: Pt disc; auxiliary electrode: Pt wire; step potential: 4 mV; pulse amplitude: 50 mV; pulse duration: 0.2 s; pulse period: 0.5 s. [b]Attained from UV-vis spectra. [c]$E_{g,opt} = 1240/\lambda_{onset}$.[10,11] [d]Estimated using the following equation: LUMO level = $-(4.8 + E_{red}^{onset})$ eV.[11,12] [e]HOMO level = (LUMO − $E_{g,opt}$) eV.[11,13] LUMO: lowest unoccupied molecular orbital. HOMO: highest occupied molecular orbital.

17. Device Fabrication and Characterisation

The FTO glass substrate was cleaned using detergent, deionised water, acetone, and isopropanol for 15 min and then dried under vacuum at 60 °C overnight. After ultraviolet-ozone treatment for 15 min, the NiO_x precursor solution prepared by the sol-gel method (0.1 mmol $Ni(CH_3COO)_2 \cdot 4H_2O$ was dissolved in 10 mL ethanol and 60 μL ethanolamine, and the solution was stirred at 70 °C overnight) was spin-coated onto the FTO substrate under ambient atmosphere at 4000 rpm for 30 s and then annealed at 280 °C for 1 h. In the following step, the FTO substrate with the NiO_x film was transferred to a nitrogen-filled glovebox to fabricate the $MAPbI_3$ perovskite layer. A perovskite precursor solution composed of PbI_2 (1.2 M) and MAI (1.2 M) in DMF and DMSO (7:3 v/v) was dropped onto the FTO/NiO_x substrate by spin-coating at 3500 rpm for 30 s. After that, 200 μL of chlorobenzene was drop-coated onto the perovskite layer during the last 20 s of the spin-coating process, and the devices were annealed on a hot plate at 100 °C for 10 min. Compound **3** (0.15 wt%) with PCBM (20 mg/mL in chlorobenzene) was spin-coated onto the $MAPbI_3$ perovskite film at 2000 rpm for 30 s to form the electron transport layer (ETL). Then, a cathode buffer layer of bathocuproine (BCP, 0.5 mg/mL) in a solution of isopropanol (IPA) was spin-coated on ETL. Finally, the Ag electrode (80 nm) was thermally evaporated onto the PCBM layer under a pressure of about 10^{-6} Torr. For space charge limited current (SCLC) devices, the FTO glass substrate was replaced with an ITO glass substrate. The active area of the devices was defined as 0.1 cm^2 by the shadow mask.

The current density-voltage *(J-V)* characterizations were performed with a Keithley 2400 source meter under simulated AM 1.5 irradiation (100 mW cm^{-2}) with a standard xenon-lamp-based solar simulator (SAN-EI·XES-50S2). The simulator illumination intensity was calibrated with a monocrystalline silicon reference cell (Newport P/N 91150 V, with KG-5 visible color filter) calibrated by the National Renewable Energy Laboratory (NREL).

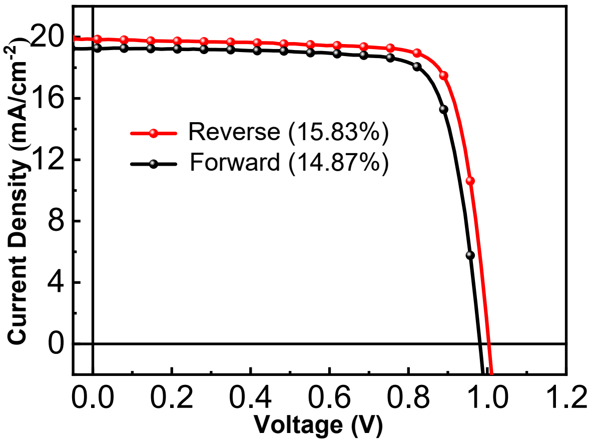

Supplementary Fig. 67. *J–V* curves of the device without 3 in different scan directions. Hysteresis index: 6.06%.

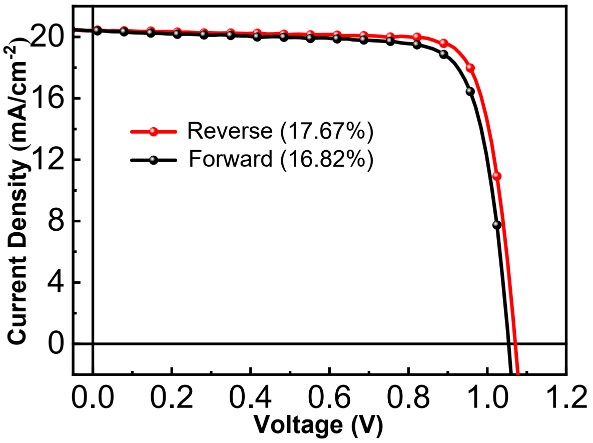

Supplementary Fig. 68. *J–V* curves of the device with 3 in different scan directions. Hysteresis index: 4.81%.

The electron mobilities (μ_e) of PSCs with structures of ITO/$CH_3NH_3PbI_3$/PCBM/Ag and ITO/$CH_3NH_3PbI_3$/PCBM:**3**/Ag were estimated by the SCLC method based on the Mott–Gurney equation:[14,15]

$$J_d = \frac{9\varepsilon\varepsilon_0}{8L^3} V^2 \mu_e \qquad \text{eq (S3)}$$

where J_d is the current density, μ_e is the electron mobility, ε_0 is the vacuum permittivity, ε represents the relative dielectric constant of $CH_3NH_3PbI_3$ and is 28.8, and L is the thickness of the $CH_3NH_3PbI_3$ film (409 nm, measured *via* a field-emission scanning electron microscope). According to equation (S3), the estimated μ_e of the reference device and the device incorporated with **3** were 7.16×10^{-4} cm^2 V^{-1} S^{-1} and 1.40×10^{-3} cm^2 V^{-1} S^{-1}, respectively.

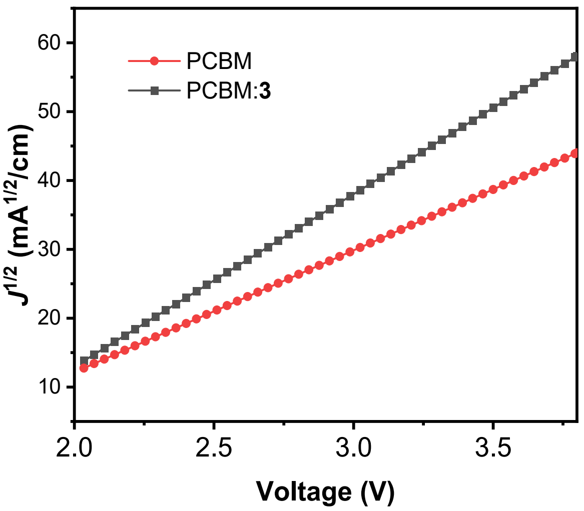

Supplementary Fig. 69. Dark $J^{1/2}$–V curves of electron-only devices with the structures of ITO/$CH_3NH_3PbI_3$/PCBM/Ag and ITO/$CH_3NH_3PbI_3$/PCBM:3/Ag. ITO: indium tin oxide. PCBM: [6,6]-phenyl-C_{61}-butyric acid methyl ester.

18. References

1. Hummelen, J. C., Knight, B. W., LePeq, F., Wudl, F., Yao, J. & Wilkins, C. L. Preparation and characterization of fulleroid and methanofullerene derivatives. *J. Org. Chem.* **60**, 532–538 (1995).
2. Mayorova, J. Y., Nikitenko, S. L., Troshin, P. A., Peregudova, S. M., Peregudov, A. S., Kaplunov, M. G. & Lyubovskaya, R. N. Synthesis and investigation of fullerene-based acceptor materials. *Mendeleev Commun.* **17**, 175–177 (2007).
3. Gaussian 09 (RevisionB.01), M. J. Frisch, et al., Gaussian Inc., Wallingford, CT (2010).
4. Sheldrick, G. M. *SHELXT* – Integrated space-group and crystalstructure determination. *Acta Cryst. A71*, 3–8 (2015).
5. Sheldrick, G. M. Crystal structure refinement with *SHELXL*. *Acta Cryst. C71*, 3–8

(2015).

6. Xu, Y.-Y., Tian, H.-R., Li, S.-H., Chen, Z.-C., Yao, Y.-R., Wang, S.-S., Zhang, X., Zhu, Z.-Z., Deng, S.-L., Zhang, Q., Yang, S., Xie, S.-Y., Huang, R.-B. & Zheng, L.-S. Flexible decapyrrylcorannulene hosts. *Nat. Commun.* **10**, 485 (2019).
7. McQuarrie, A, *Statistical Mechanics* (University Science Books, California, 2000).
8. Gao, X., Gallicchio & E. Roitberg, A. E. The generalized Boltzmann distribution is the only distribution in which the Gibbs-Shannon entropy equals the thermodynamic entropy. *J. Chem. Phys.* **151**, 034113 (2019).
9. Huang, X.-Y., Xie, P.-P., Zou, L.-M., Zheng, C. & You, S.-L. Asymmetric dearomatization of indoles with azodicarboxylates via cascade electrophilic amination/Aza-Prins cyclization/phenonium-like rearrangement. *J. Am. Chem. Soc.* **145**, 11745–11753 (2023).
10. Wang, M., Hu, X., Liu, P., Li, W., Gong, X., Huang, F. & Cao, Y. Donor–acceptor conjugated polymer based on naphtho[1,2-*c*:5,6-*c*]bis[1,2,5]thiadiazole for high-performance polymer solar cells. *J. Am. Chem. Soc.* **133**, 9638–9641 (2011).
11. Li, B., Zhen, J., Wan, Y., Lei, X., Liu, Q., Liu, Y., Jia, L., Wu, X., Zeng, H., Zhang, W., Wang, G.-W., Chen, M. & Yang, S. Anchoring fullerene onto perovskite film via grafting pyridine toward enhanced electron transport in high-efficiency solar cells. *ACS Appl. Mater. Interfaces* **10**, 32471–32482 (2018).
12. Wong, W.-Y., Wang, X.-Z., He, Z., Djurii, A. B., Yip, C.-T., Cheung, K.-Y., Wang, H., Mak, C. S. K. & Chan, W.-K. Metallated conjugated polymers as a new avenue towards high-efficiency polymer solar cells. *Nat. Mater.* **6**, 521–527 (2007).
13. Li, N. & Brabec, C. J. Air-processed polymer tandem solar cells with power conversion efficiency exceeding 10%. *Energy Environ. Sci.* **8**, 2902–2909 (2015).
14. Li, B., Zhen, J., Wan, Y., Lei, X., Jia, L., Wu, X., Zeng, H., Chen, M., Wang, G.-W. & Yang, S. Steering the electron transport properties of pyridine-functionalized fullerene derivatives in inverted perovskite solar cells: the nitrogen site matters. *J. Mater. Chem. A* **8**, 3872–3881 (2020).
15. Wang, N., Zhao, K., Ding, T., Liu, W., Ahmed, A. S., Wang, Z., Tian, M., Sun, X. W. & Zhang, Q. Improving interfacial charge recombination in planar heterojunction perovskite photovoltaics with small molecule as electron transport layer. *Adv. Energy Mater.* **7**, 1700522 (2017).
